# Decline in CD4 T lymphocytes with monotherapy bridging strategy for non-adherent adolescents living with HIV infection: Results of the IMPAACT P1094 randomized trial

**DOI:** 10.1371/journal.pone.0178075

**Published:** 2017-06-12

**Authors:** Allison L. Agwu, Meredith G. Warshaw, Elizabeth J. McFarland, George K. Siberry, Ann J. Melvin, Andrew A. Wiznia, Lee Fairlie, Sandra Boyd, Paul Harding, Hans M. L. Spiegel, Elaine J. Abrams, Vincent J. Carey, P1094 Study Team

**Affiliations:** 1 Johns Hopkins School of Medicine, Departments of Pediatrics and Internal Medicine, Division of Infectious Diseases, Baltimore, Maryland, United States of America; 2 Center for Biostatistics in AIDS Research, Harvard School of Public Health, Boston, Massachusetts, United States of America; 3 Department of Pediatrics, University of Colorado School of Medicine and Children's Hospital Colorado, Pediatric Infectious Diseases, Aurora, Colorado, United States of America; 4 Maternal Pediatric Infectious Disease Branch, *Eunice Kennedy Shriver* National Institute of Child Health and Human Development, Bethesda, Maryland, United States of America; 5 Seattle Children’s Hospital, Division of Pediatric Infectious Disease, Seattle, Washington, United States of America; 6 Pediatric HIV Services, Jacobi Medical Center/Family Based Services, Bronx, New York, United States of America; 7 Wits Reproductive Health & HIV Research Institute, University of the Witwatersrand, Johannesburg, Republic of South Africa; 8 St. Jude Children's Research Hospital, Memphis, Tennessee, United States of America; 9 Kelly Government Solutions, Contractor to Division of AIDS, PMPRB/Prevention Sciences Program, Division of AIDS, NIAID, NIH, Rockville, Maryland, United States of America; 10 ICAP at Columbia, Mailman School of Public Health and College of Physicians & Surgeons, Columbia University, New York, New York, United States of America; Azienda Ospedaliera Universitaria di Perugia, ITALY

## Abstract

**Introduction:**

Management of persistently non-adherent youth living with HIV (YLHIV) with virologic failure (VF) on combination antiretroviral therapy (cART) remains challenging. One strategy has been using 3TC/ FTC monotherapy (3TC/FTC), which in the presence of the M184V resistance mutation, does not suppress viral replication nor select for additional drug resistance mutations, and reduces viral fitness with limited side effects. P1094 compared the immunologic outcome of continuing failing cART vs. switching to 3TC/FTC as a “bridging strategy” to subsequent suppressive cART for non-adherent YLHIV with pre-existing M184V resistance.

**Materials & methods:**

Participants with documented nonadherence, M184V mutation, CD4^+^ T cell count ≥100 cells/mm^3^ and VF (HIV-1 plasma RNA ≥400 copies/mL (2.6 log_10_ HIV-1 RNA) were enrolled and randomized to continue failing cART vs. switch to 3TC/FTC. The primary endpoint (time to ≥30% CD4^+^ T cell decline or development of CDC class C events) at 28-weeks were assessed by Kaplan-Meier (K-M) curves in an intent-to-treat analysis.

**Results:**

Thirty-three perinatally acquired YLHIV participants (16 continuing cART and 17 3TC/FTC) enrolled in the study. The median age, entry CD4^+^ T cell count, and viral load were 15 years (Inter-quartile range (IQR) 14–20), 472 cells/mm^3^ (IQR 384–651), and 4.0 log_10_HIV-1 RNA copies/ml (IQR 3.2–4.5), respectively. Five participants, all in the 3TC/FTC arm, reached the primary endpoint for absolute CD4^+^ T cell decline (p = 0.02, exact log-rank test comparing monotherapy to cART). The Kaplan-Meier estimate of probability of primary endpoint on 3TC/FTC at 28 weeks was 0.41 (standard error 0.14). There were no CDC class C events or deaths and no statistically significant difference in frequencies of adverse events between the arms.

**Conclusions:**

Non-adherent participants randomized to 3TC/FTC were more likely than those maintained on failing cART to experience a confirmed decline in CD4+ count of ≥30%. Although this study suffers from limitations of small sample size and premature discontinuation, the randomized comparison to continuing failing cART indicates that 3TC/FTC provides inferior protection from immunologic deterioration for non-adherent youth with M184V resistance. Better alternatives to 3TC/FTC such as ART with higher barriers to resistance and novel adherence and treatment strategies for nonadherent youth are urgently needed.

**Trial registration:**

Clinical Trials.gov NCT01338025

## Introduction

Durable virologic suppression using combination antiretroviral therapy (cART) is most reliably achieved for patients with excellent adherence, with small amounts of nonadherence conferring risk of emergent resistance and viral load breakthrough [1, 2]. However, it is estimated that up to 40% of pediatric and adolescent patients who initiate a new regimen fail on that regimen within the first 1–2 years [3–5], primarily due to nonadherence. This suboptimal adherence, with viremia in the presence of continued drug pressure at suboptimal levels, results in development of resistant quasispecies and viral escape. Nonadherence with resultant viremia affects 30–40% of children and youth with perinatally acquired HIV infection (PA-HIV) on cART[6–8], and is likely due to multiple causes (treatment fatigue, pill burden, desire for normalcy, depression, transition of the responsibility of medication administration from the caregiver to the child) that may function independently or in concert[9].

Antiretroviral management in the setting of poor adherence in children and youth living with HIV is increasingly challenging for providers. A balance must be struck between the need to continue cART to maintain immunologic integrity and the desire to simplify the therapeutic regimen, foster optimal adherence, and minimize development of resistance, while awaiting for youth to mature and develop better adherence. To improve adherence, multiple strategies have been tested (e.g., cell phone reminders, counseling, MEMS caps, G-tube placement, directly observed therapy, financial incentives), yet no gold standard has emerged [9, 10]. However, during the time providers are working to improve adherence, nonadherence frequently continues with the attendant risk of accumulation of ARV drug resistance and reduction of future treatment options. The question of what to do with the cART while working on adherence remains a centrally important one in the management of persistently nonadherent children and youth living with PA-HIV in need of therapy and there are no consensus guidelines about how this should be managed. Switching to a new regimen in the setting of nonadherence, particularly, when nonadherence is unrelated to side effects, may lead to development of resistance to the new regimen. Discontinuation of cART is generally avoided in patients who meet treatment criteria, in light of data from the SMART study that highlights the increased morbidity risk secondary to HIV-related and non-related clinical events with treatment discontinuation[11]. Bridging strategies, without resistance consequences, could provide time for intensive efforts to improve adherence, which might improve the chance that these patients would succeed on a subsequent cART regimen. One such strategy is lamivudine (3TC) or emtricitabine (FTC) monotherapy. The rationale for this strategy is as follows: 1) 3TC or FTC are components of the nucleoside/nucleotide reverse transcriptase inhibitor (NRTI) backbone of most first line cART regimens, 2) 3TC and FTC have minimal side effects, 3) the M184V resistance mutation, resulting in high-level drug resistance to both 3TC and FTC, develops rapidly in the setting of nonadherence and is present in many treatment-experienced children and adolescents with PA-HIV [7, 12, 13], 4) the M184V resistant mutation reduces viral fitness, and has been associated with a reduction in viral load of approximately 0.5 log_10_ c/mL[14], and 5) it does not confer significant cross-resistance to other NRTIs and increases susceptibility of HIV to other antiretroviral (ARV) drugs [e.g., tenofovir disproxil fumarate (TDF), zidovudine (AZT)][15]. Once the M184V mutation develops, no additional mutations occur with further use of 3TC or FTC monotherapy.

This strategy has been shown to be superior to discontinuing all antiretroviral therapy in adults[16]. Observational experience of PI interruption and continuation of dual-NRTI for children and youth with persistent viremia due to nonadherence, toxicity, and limited treatment options showed unchanged viremia, but relative safety of NRTI simplification with sustained CD4%>15, although significant declines did occur[17]. At the time the study was developed, 3TC/FTC monotherapy was listed as a potential option in the U.S. pediatric antiretroviral treatment guidelines for patients with persistent viremia with NRTI resistance[18]; and was and still is employed in settings where access to second and third line antiretroviral agents and/or persistent nonadherence has led to limited treatment options[19–22]. The primary objective of the P1094 study was to compare 3TC/FTC monotherapy to continuing failing cART in patients with persistent nonadherence.

## Materials & methods

International Maternal Pediatric Adolescent AIDS Clinical Trials (IMPAACT) P1094 was a multi-center (domestic and international), open-label, Phase IV randomized, controlled, comparative trial to evaluate continuation of non-suppressive cART (Arm A) vs. 3TC/FTC monotherapy (Arm B) for 28 weeks (in both arms) (NCT01338025).[23] All of the participating sites are part of the IMPAACT network of clinical research sites. Participants were (perinatally or non perinatally) HIV-infected children, adolescents, and young adults ≥8 to <25 years old, on a non-NNRTI (non-nucleoside reverse transcriptase inhibitor) regimen for at least 6 months, with documented nonadherence (e.g., self-report or pharmacy report, despite documented interventions to improve nonadherence), M184V resistance mutation at or prior to screening on standard, clinically available genotyping assays, absolute CD4+ T cell count ≥100 cells/mm^3^, and VF (defined as HIV-1 plasma RNA ≥400 copies/mL on at least two occasions including screening at least 2 months after initiating the regimen and within 6 months of study entry). When slow enrolment was observed, a site survey was conducted and in response, the following changes were made to the eligibility criteria (March 7 2012): absolute CD4+ T cell count was lowered from 250 to 100 cells/mm^3^ and the time demonstrating failure from 6 months to 2 months.

Main exclusion criteria included hepatitis B infection, active malignancy within the prior two years, active opportunistic or other infection; ≥ 1 CDC class C event within 12 months of entry, or viral load >250,000 copies/mL at screening. Participants were enrolled between May 2011 and January 2013 and randomized 1:1 to the two treatment groups using a dynamic permuted block system with institutional balancing (maximum institutional imbalance of 2), stratified by CD4+ T cell count (<400 cells/mm^3^ versus ≥400 cells/mm^3^). Participants were enrolled online and randomized at time of enrollment via computer algorithm at the Data Management Center.

For participants assigned to Arm A, the protocol required that the participant continue their current ART regimen, which was prescribed and monitored by the participant’s provider. Participants assigned to the monotherapy arm (Arm B) received either 3TC or FTC (the choice of 3TC or FTC was left to the provider) and discontinued other ART. Sites were expected to provide and document adherence support measures (per their standard of care) during the study. The target sample size of 344 was chosen to provide 80% power to detect a difference of 0.15 between study arms in proportions of participants with immunologic deterioration using a log-rank test with a 2-sided Type I error rate of 0.05. Due to poor accrual, the final sample size was 32 eligible participants. The antiretroviral agents were not provided by the study. The study was approved by the Institutional review boards (IRBs) of all participating clinical research sites (CRS) (Bronx-Lebanon CRS, New York, United States; Chiang Mai University Pediatrics-Obstetrics CRS, Thailand; Children's National Med. Ctr. Washington DC NICHD CRS, United States; Duke University Medical Center CRS, Durham, North Carolina, United States; Hosp. General de Agudos Buenos Aires Argentina NICHD CRS; Hosp. Geral De Nova Igaucu Brazil NICHD CRS; Inst de Infectologia Emilio Ribas, Sao Paulo Brazil NICHD CRS; and Johns Hopkins Univ. Baltimore NICHD CRS, United States. All participating sites received ethics approval prior to study initiation at their site. Approvals were received from April 13, 2011 to March 7, 2012. Participants and their parent/guardian, where relevant, signed written informed consent, which was documented in the participant’s chart and/or study log as directed by their local IRB requirements. A data safety monitoring board (DSMB) monitored the study conduct throughout the study.

### Study measurements

Study measurements included lymphocyte subsets at each study visit (0, 4, 12, 20, 28 weeks), HIV RNA testing (0, 4, 12, 28 weeks), and safety monitoring labs including hematology (0, 4, 12, 20, 28 weeks and chemistries (0, 12, 28 weeks). Plasma and peripheral blood mononuclear cells (PBMCs) for inflammatory and immune activation markers were collected at 0, 12, and 28 weeks. Self-reported adherence was also assessed at each study visit by collection of 3-day recall and evaluation of interventions employed to enhance adherence. Batched resistance testing was planned as part of the protocol, however, this was not performed when the study ended early.

### Immunology assays

Plasma and Ficoll-Hypaque density gradient isolated peripheral blood mononuclear cells (PBMCs) were cryopreserved within 8 hours of blood draw. Plasma markers of inflammation and immune activation were determined with ELISA kits, using duplicate samples on the same plate per subject, for D-Dimer (Imuclone/Sekisui Diagnostics, Lexington, MA), sVCAM-1 (Millipore, Billerica, MA), and soluble CD4 (sCD14), interleukin-6 (IL-6), and high sensitivity C-reactive protein (hsCRP) (R&D Systems, Minneapolis, MN). These markers were selected based on correlation with morbidity and mortality in studies of HIV-1 infected adults[23–27]. Multi-parameter flow cytometric analysis (LSRII, Becton Dickinson, Franklin Lakes, NJ; FlowJo Version 9.6.1, Treestar, Ashland, OR) of cryopreserved PBMC stained with CD3-FITC and CD4-Qdot605 (Invitrogen/Life technologies, Grand Island, NY), CD8-AF700 and HLA-DR-APC (BD Biosciences, San Jose CA), CD38-PE, CD95-Pacific Blue, CD31-PE-CY7, CD27-APC-CY7, CD45RO-PE-CY5 (Biolegend, San Diego, CA), and LIVE/DEAD Fixable Aqua Dead Cell Stain (Invitrogen/Life technologies, Grand Island, NY) was performed. The percent of naive (CD27^+^ CD45RO^-^ CD95^-^) and activated (CD38^+^ HLA-DR^+^) cells within total CD3^+^/CD4^+^ and CD3^+^/CD8^+^ viable lymphocytes was determined. In addition, the percentage of recent thymic emigrants (RTE) (CD31^+^ CD27^+^ CD45RO^-^ CD95^-^) among CD3^+^/CD4^+^ lymphocytes was determined[28].

### Statistical analysis

The primary endpoint was time to time to immunologic deterioration, defined as first ≥30% decline in absolute CD4+ T cell count from baseline or development of CDC class C events. Additional analyses were done on rates of adverse events or deaths, change in viral load, changed in biomarkers of inflammation and immune activation, and adherence at the end of follow-up; as well as baseline immune characteristics on the 3TC/FTC monotherapy arm. Kaplan-Meier curves and log-rank tests were used to compare the treatment arms with respect to time to immunologic failure.[29] The probability of avoiding immunologic deterioration by 28 weeks was estimated via the method of Kaplan and Meier, and the 95% confidence intervals (CIs) for this probability were computed for each arm. Linear mixed effects models were used to estimate, in an exploratory manner, rates of change in CD4+ T cell counts and viral load levels over time for the two arms.[30] Three-day recall of adherence was categorized as missed doses in the past 3 days (yes/no) at entry and 28 weeks. The proportion of participants at week 28 that were in the different categories of adherence, as measured by 3-day recall, was compared between the two study arms using Fisher’s exact test. Lastly, as an exploratory objective, changes in immune activation, inflammatory biomarkers, and LDL and HDL cholesterol were assessed for participants in the 3TC/FTC arm followed at domestic sites only, and compared between groups formed by presence or absence of immunologic deterioration by week 28 using Kruskal-Wallis tests. All analyses were intent-to-treat and included all eligible participants unless otherwise specified. All tests were done using SAS 9.4, StatXact.

## Results

**Fig 1:**
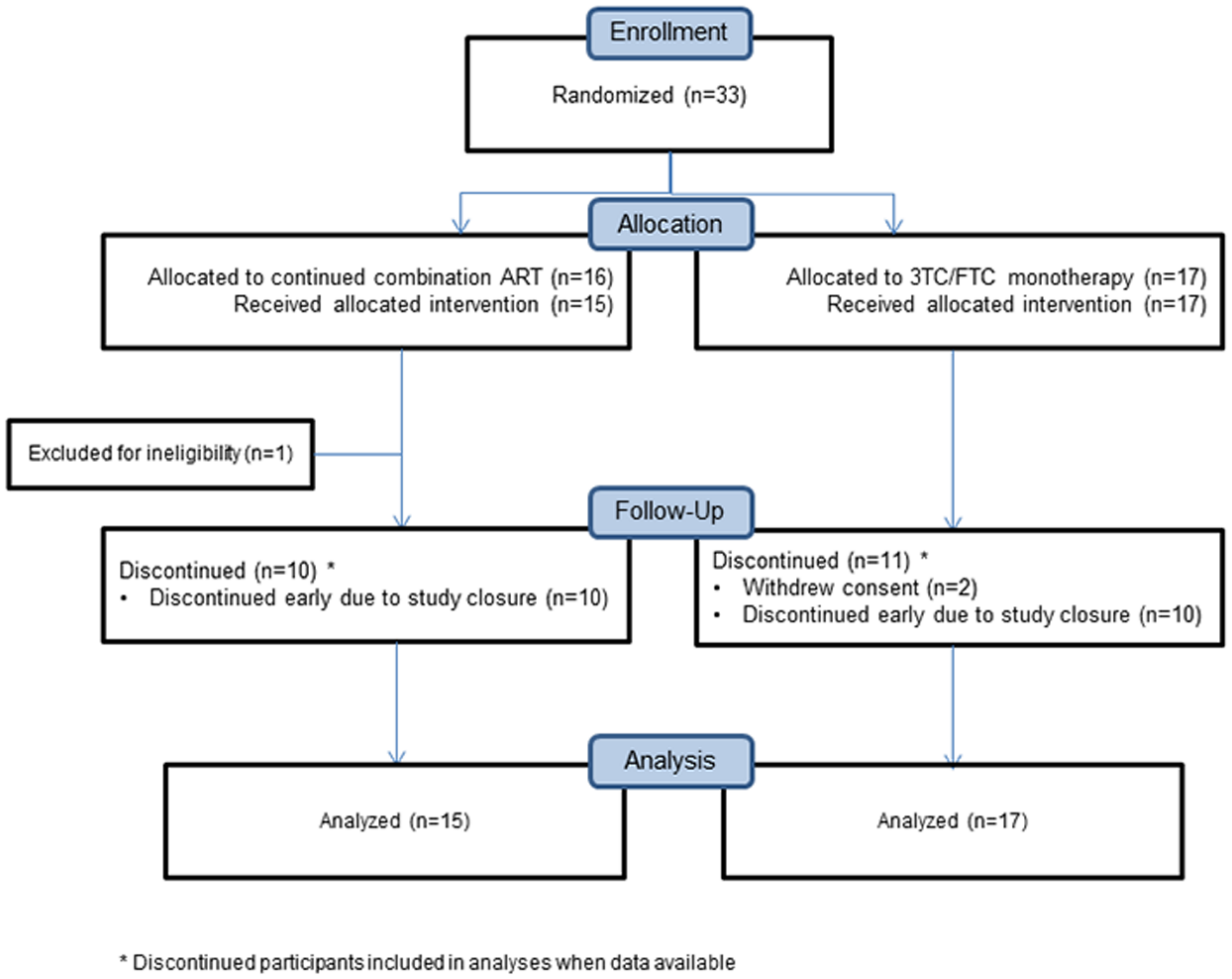
CONSORT diagram for P1094.

Enrollment occurred May 10, 2011 through January 16, 2013 and the study was discontinued in February 2013 due to slow accrual; all participants were off study by May 15, 2013. Thirty-three participants were enrolled, out of targeted enrollment of 344, of whom one was ineligible and did not have any post-entry visits (Fig 1). We present the results for the 32 perinatally HIV-infected participants enrolled prior to study closure (15 randomized to continuing cART and 17 to 3TC/FTC). While the study was open to both perinatally and non-perinatally HIV-infected individuals, only PHIV were enrolled. Participants were from diverse regions (Argentina n = 3, Brazil n = 5, Thailand n = 6, U.S. n = 18). The demographic and clinical characteristics of the participants are presented in Table 1. Participants were median age 15 years (Inter-quartile range (IQR) 14–20), 68% female, and 52% black. Median entry CD4+ T cell count and viral load were 472 cells/mm^3^ (IQR 384–651) and 4.0 log_10_HIV-1 RNA copies/ml (IQR 3.2–4.5), respectively. A median of 4 interventions (e.g., counseling, text reminders, directly observed therapy) had been used to address nonadherence prior to study. The median duration on study intervention of 27 weeks (IQR 12–34) was not different between the two treatment arms.

**Table 1:** Baseline characteristics of participants enrolled in P1094.

		Treatment		
Characteristic		cART (N = 15)	3TC/FTC (N = 17)	Total (N = 32)
Age at Entry (Years)	Median (Q1, Q3)	18 (14, 20)	15 (13, 20)	15.0 (13.5, 20.0)
	Min, Max	11, 24	10, 21	10, 24
	10–14	5 (33%)	6 (35%)	11 (34%)
	15–17	2 (13%)	5 (29%)	7 (22%)
	18–24	8 (53%)	6 (35%)	14 (44%)
Sex	Male	4 (27%)	7 (41%)	11 (34%)
	Female	11 (73%)	10 (59%)	21 (66%)
Race	Asian	2 (13%)	3 (18%)	5 (16%)
	Black or African American	9 (60%)	8 (47%)	17 (53%)
	White	4 (27%)	6 (35%)	10 (31%)
Ethnicity	Hispanic or Latino	6 (40%)	8 (47%)	14 (44%)
	Not Hispanic or Latino	8 (53%)	9 (53%)	17 (53%)
	Unknown	1 (7%)	0 (0%)	1 (3%)
Entry RNA log_10_(copies/ml)	Median (Q1, Q3)	4.2 (3.0, 4.8)	4.0 (3.2, 4.1)	4.0 (3.1, 4.5)
	Min, Max	2.2, 5.6	2.2, 4.9	2.2, 5.6
Entry CD4 (cells/mm^3^)	Median (Q1, Q3)	476 (361, 669)	444 (350, 654)	472 (356, 662)
	Min, Max	257, 848	160, 1191	160, 1191
# reasons used to determine sub-optimal adherence*	Median (Q1, Q3)	3 (2, 3)	2 (2, 3)	3 (2, 3)
	Min, Max	1, 6	1, 5	1, 6
# interventions used to address non-adherence^⊥^	Median (Q1, Q3)	4 (3, 7)	4 (3, 5)	4 (3, 6)
	Min, Max	1, 9	1, 9	1, 9

*Nonadherence: admission of incomplete adherence (2+ occasions taking <90% of meds)(78%), persistent viremia without plausible explanation (69%), agreement of 2 providers (59%), miss and doses in past 3 days (non-adherence questionnaire) (53%), pharmacy refill history (34%), pill counts (22%), drug levels (6%), other (3%)

^⊥^Interventions attempted: counseling (94%), frequent clinic visits (72%), reminders (63%), therapy (44%), peer support (38%), ADL triggers (38%), regimen modification/simplification (31%), rewards (31%), home visits (22%), direct observation (9%), G-tube (6%), other (9%)

### Immunologic deterioration

**Fig 2:**
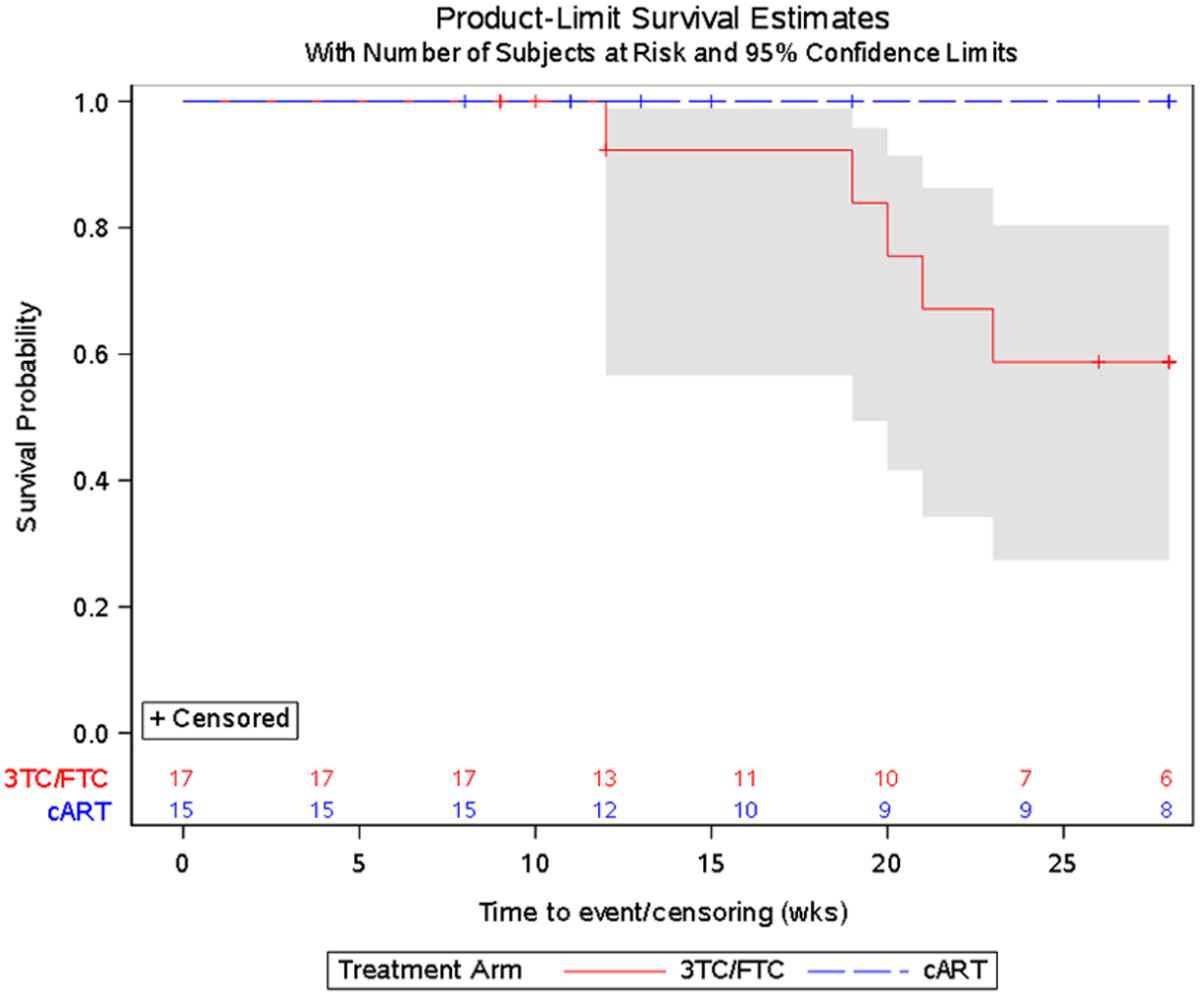
Probability of not experiencing immunologic failure.

Five participants, all in the 3TC/FTC monotherapy arm, reached the primary endpoint for decline in CD4+ T cell count (exact log-rank test p = 0.02, Fig 2). The Kaplan-Meier estimate of probability of immunologic failure at 28 weeks was 0.41 (standard error 0.14). No participants experienced CDC Class C events.

To test the null hypothesis of identical rates of change in CD4+ T cell counts over time on study, repeated measures modeling was used. The model (Table 2) included main effects of time on study and treatment assignment, the product of time on study and treatment assignment, adjustments for patient age and sex, and a random intercept to accommodate between-patient variability. The coefficient of the product term permits estimation of the interaction between study week and treatment arm, with participants on the 3TC/FTC arm estimated to lose 5.31 cells/mm^3^ per week (p = 0.03) while those on the cART arm were estimated to gain 3.82 cells/mm^3^ per week (p = 0.10). The median (IQR) change in CD4+ T cell count over the study period was 44 (-16, 142) for the cART arm and -70 (-329, -18) for the 3TC/FTC arm (Kruskal-Wallis test, p = 0.004).

**Table 2:** Random effects mixed model for CD4 decline.

	Treatment		95% Confidence Limits	
Effect*	Arm	Estimate	(Lower, Upper)	P
Intercept	_	922.5	(495.3, 1349.7)	<0.001
Age	_	-25.0	(-46.7, -3.3)	0.02
Female	_	-3.3	(-171.7, 165.0)	0.97
Week*Treatment	3TC/FTC	-5.3	(-9.9, -0.7)	0.03
Week*Treatment	HAART	3.8	(-0.7, 8.4)	0.10

*The week by treatment interaction terms represent the rates of change over time for each treatment

### Adverse events, deaths, change in viral load, and adherence

There were no deaths. There was only one grade 3 or higher adverse event reported; one participant on the cART arm had a grade 4 total bilirubin, attributable to receiving atazanavir. There were no significant differences in self-reported adherence between the two arms at baseline or during the study period. However, compared to baseline, at the end of the study period, fewer participants in both treatment arms reported missing doses in the prior 3 days, 47% at baseline vs. 33% at the end in the cART arm and 59% vs. 18% in the 3TC/FTC arm. Participants in the cART arm had a median decline in viral load of -3.78 (IQR -4.25 to -2.25) Iog_10_HIV-1 RNA copies vs. an increase of 3.87 (IQR -3.73 to 4.48) log_10_HIV-1 RNA copies/ml in the 3TC/FTC arm (p = 0.08), although the difference was not statistically significant. Four (67%) of the six participants on cART with viral load data for week 24/28 had decreases in log viral load of more than 0.5, while none of the six participants on 3TC/FTC did (p = 0.06, Fisher’s exact test.)

### Immunology and immune activation

Biomarkers of inflammation and immune activation were assessed for participants in the 3TC/FTC arm followed at domestic sites with available samples. Fourteen participants had biomarker data (sCD14, IL-6, hsCRP, sVCAM-1, and D-dimer) available at both baseline and Week 24/28 (9 in the 3TC/FTC arm and 5 in the cART arm). There were no significant differences between arms in the change from baseline for any of the markers (Table 3). As an exploratory analysis, baseline immune characteristics were examined among the participants in the 3TC/FTC monotherapy arm to determine whether there were correlations with subsequent immune deterioration. There were no differences in any of the baseline plasma markers for those that did (n = 7) or did not (n = 4) have a ≥30% decline in absolute CD4 + T cell count. There were also no differences in baseline frequency of activated CD4+ or CD8+ T cells nor the frequency of CD4+ T cells that were naïve or recent thymic emigrants (S1 and S2 Tables). Given the restricted sample size, only large differences could be detected.

**Table 3:** Immune activation and inflammation biomarker change from baseline to week 24/28.

Biomarker	Median change from baseline (IQR)		P-value*
	cART N = 5	3TC/FTC N = 9	
Soluble CD4 (ug/ml) ^⊥^	0.1 (-0.9, 0.3)	-0.1 (-0.1,0.1)	0.84
Interleukin-6 (pg/ml) ^⊥^	-1.2 (-2.8, 1.0)	-1.7 (-1.7,-.03)	0.74
hsCRP (ng/ml) ^⊥^	2.0 (0.5, 2.2)	0.1 (-.03, 0.5)	0.21
sVCAM-1 (ng/ml) ^⊥^	-40 (-148, 338)	-307 (-658, 329)	0.22
D-dimer (ng/ml) ^⊥^	-146 (-718, 31)	-28 (-40,44)	0.26

* Wilcoxon Test

^⊥^Plasma biomarkers of immune activation and inflammation determined by ELISA.

IQR, interquartile range; cART, combination antiretroviral treatment; 3TC, lamivudine; FTC, emtricitabine, hsCRP, high sensitivity C-reactive protein; sVCAM-1, soluble vascular cellular adhesion molecule-type 1; D-dimer

## Discussion

To our knowledge, this is the first and only randomized control trial comparing 3TC/FTC monotherapy to continuing cART in any population of individuals living with HIV. In this multi-site study of YLHIV with persistent virologic failure on cART, those who were randomized to 3TC/FTC monotherapy were significantly more likely to experience ≥30% decline in absolute CD4+ T cell count over 28 weeks compared with those maintained on failing cART. The change from baseline in markers of inflammation and immune activation did not differ between the two groups. Immune correlates at baseline were not different between those who did and did not experience immunologic decline in the 3TC/FTC arm, though this was performed on a limited sample size.

A prior study of adults with relatively robust baseline median CD4+ T cell counts of 570 (IQR 466–636) cells/mm^3^ compared 3TC monotherapy to discontinuation of cART and found that the monotherapy strategy was superior to cART discontinuation [16]. Specifically, participants with lower CD4+ T cell counts were more likely to have clinical deterioration than those at higher CD4+ T cell counts. We were unable to conduct an analysis of higher vs. lower CD4+ T cell counts at entry on clinical deterioration due to both a limited sample size and the majority of our participants having CD4 + T cell counts >350 cells/mm^3^. Others have shown that withdrawing the NRTIs as opposed to interrupting PIs or NNRTIs in a failing regimen may have the greatest impact on immune decline, a different approach than was used in our study [31].

It is possible that the use of the comparison cART group on PI-based regimens may have led to the dramatically different outcomes between the two study arms. Opravil et al studied 26 clinically stable, significantly pre-treated adults (median age 43 years; median CD4+ T cell count of 432 [IQR 378–540] cells/mm^3^) who were switched in an uncontrolled, one-armed study from a failing (VL> than 400 copies/mL) cART regimen to 3TC monotherapy[32]. The proportion of patients reaching the clinical endpoint of a ≥ 30% decline in CD4+ T cell count or a CD4+ T cell count <200 cells/mm^3^ at 24, 36, and 48 weeks was 36%, 57%, and 70%, respectively. When the investigators analyzed the participants stratified by the failing regimen at baseline (PI vs. reverse transcriptase inhibitor (RTI)-based cART (NNRTI or NRTI)), 81% of participants on PI-based cART vs. 40% of participants on RTI-based cART reached the clinical endpoint, despite similar baseline CD4+ T cell counts and other characteristics. The decline in absolute CD4+ T cell was not statistically different between the two groups, however, patients who were initially on PI-based cART had greater increases in the viral replication capacity, perhaps due to outgrowth of viral strains lacking PI resistance mutations detected on resistance testing in the absence of ART pressure. The authors concluded that 3TC monotherapy may be considered as a short-term strategy for “selected” patients failing RTI-based cART, but not PI-based regimens due to concern for increased viral fitness and greater likelihood of CD4+ T cell decline. Our study is unable to look at differences between PI vs. RTI-based cART as NNRTI-based regimens were excluded. Given the known lower barrier to resistance in NNRTI-based regimens and the possibility of rapid accumulation of resistance in the setting of continued nonadherence, we did not feel that it was ethical to randomize participants to continue NNRTI-based regimens in setting of continued nonadherence.

During our study, participants in the continuing cART arm had declines in their viral loads, while those in the 3TC/FTC arm had increases in their viral loads, though the difference between arms was not statistically significant. It is possible that participation in a clinical trial with close monitoring, frequent interactions, and incentives may have resulted in greater adherence over the study period. Indeed fewer participants in both arms reported missed doses at the end of the study compared to baseline, suggesting that the frequent visits and/or other study interventions with the site’s ongoing adherence work led to improvements in adherence even in these patients with a history of persistently poor adherence. Although there was no difference between the two arms in terms of adherence, it is possible that improved, though suboptimal adherence to a potentially more active regimen in the cART arm would result in better viral load responses[33].

Data from observational pediatric studies of 3TC/FTC monotherapy[19] have supported 3TC/FTC monotherapy as a strategy that can be used to bridge patients during periods of nonadherence[21, 22]. Country level guidelines, including those in South Africa and previously, the U.S., listed this strategy as one that could be employed[34, 35]. Based partly on the findings from P1094 which were presented in abstract form at the 2014 Pediatric Virology Meeting in Melbourne, Australia, the U.S. Department of Health and Human Services’ Guidelines, classified NRTI monotherapy regimens, including 3TC/FTC, as “not recommended as a treatment strategy for children failing non-suppressive ART”[36]. In other countries, particularly settings with limited antiretroviral options, such as South Africa, this strategy continues to be used although clinicians do have limited access to third line drugs[34] [21, 22].

The study findings are specific to 3TC/FTC monotherapy, and are not meant to be extrapolated to other monotherapy bridging or simplification strategies. There have been studies of other monotherapy strategies (e.g., PI monotherapy with lopinavir/ritonavir, darunavir/ritonavir, or atazanavir/ritonavir) that have demonstrated the ability to maintain immunologic integrity, however, these strategies have been employed in the setting of virologic suppression or simplification of multi-agent ART with simplification[37]. This study was not designed to compare 3TC/FTC monotherapy to other simplified strategies and therefore cannot provide evidence for how 3TC/FTC monotherapy would compare to those strategies.

The P1094 study has several important strengths. Specifically, it was a randomized controlled study conducted through a highly experienced network, with standardized comprehensive measurements, including immunology and immune activation. Further, the participants were from multiple sites in the U.S., South America, and Thailand. The participants had a wide range of CD4+ T cell counts and were failing on contemporary antiretroviral treatment regimens. The study findings must be interpreted with some important study limitations in mind. First, the early closure due to slow accrual resulted in a small sample size, leaving the study under-powered to make certain comparisons and conclusions. Further, ARV drug exposure (e.g., drug levels) were not collected to corroborate self-reported adherence. A relatively healthy population participated with median initial CD4+ T cell counts > 500 cells/mm^3^, therefore, we cannot generalize to those with lower baseline CD4+ T cell counts. However, one would anticipate, as was seen in the Castagna study[16], that such a strategy would be even more deleterious in that population with less CD4+ T cell reserve. Limited conclusions can be drawn regarding immune activation and inflammation as a limited proportion of the participants had available data. Due to the early closure, we do not have resistance data. As some clinicians may choose 3TC/FTC monotherapy with its decreased risk of accumulating resistance as a strategy for managing youth with adherence difficulties, the lack of resistance data limits our ability to analyze for differential acquisition of resistance mutations between the two study arms. Lastly, the participants in the study may not be representative of all PHIV youth and therefore our study findings may not be generalizable to all populations of PHIV children and youth.

The findings of this first randomized controlled trial to evaluate 3TC/FTC monotherapy vs. continuing failing cART, do not support the use of 3TC/FTC monotherapy as a strategy to address nonadherence in patients failing cART, particularly in settings such as the U.S., where there are other options available, even in the setting of drug resistance. Clinicians will need to continue to assess the specific barriers and options for their young patients who are failing cART and struggling with adherence in order to make the best care decisions with and for their patients; the poor immunologic outcomes observed with 3TC/FTC monotherapy should be factored into those decisions. In all settings, the focus needs to be on increasing the availability of ARV regimens that have high barriers to resistance, low pill burden, and are available in suitable formulations and durably sustainable delivery mechanisms for youth. High priorities include fixed dose combination formulations for second and third line regimens, long-acting, injectable, and other novel cART delivery systems, as well as innovative ways of delivering care and enhancing adherence, particularly for youth, given their challenges with medication adherence.

## Supporting information

S1 File CONSORT checklist. (DOC)

S2 File Protocol. (DOC)

S1 Table Baseline immune characteristics on 3TC/FTC monotherapy arm by immunologic deterioration. (DOCX)

S2 Table Baseline immune activation of 3TC/FTC monotherapy arm by immunologic deterioration. (DOCX)
