# Supplementary material for: Decline in CD4 T lymphocytes with monotherapy bridging strategy for non-adherent adolescents living with HIV infection: Results of the IMPAACT P1094 randomized trial
Source: PLoS One. 2017 Jun 12;12(6):e0178075. doi: 10.1371/journal.pone.0178075 (PMC5467803; doi:10.1371/journal.pone.0178075)
Supplement: S2 File — (DOC) [file pone.0178075.s002.doc]

**IMPAACT P1094**

**Evaluation of 3TC or FTC Monotherapy Compared to Continuing HAART as a Bridging antiretroviral Strategy in persistently non-adherent Children, Adolescents, and young adults who are failing haart and have the M184V Resistance Mutation**

**A Multicenter, Domestic and International Trial of the**

**International Maternal Pediatric Adolescent AIDS**

**Clinical Trials Group (IMPAACT)**

**Sponsored by:**

**The National Institute of Allergy and Infectious Diseases (NIAID)**

**and**

**The Eunice Kennedy Shriver National Institute of Child Health and Human Development (NICHD)**

**Non-IND Protocol**

**The IMPAACT HIV Treatment Scientific Committee Chair: Elaine Abrams, M.D.**

**Protocol Chair: Allison L. Agwu, M.D., Sc.M.**

**Protocol Co-Vice Chairs: Jonathan M. Ellen, M.D.**

**Ann J. Melvin, M.D., M.P.H.**

**NIAID Medical Officer: Hans M.L. Spiegel, M.D., DrMedSc, DTMH, FAAP**

**NICHD Medical Officer: George K. Siberry, M.D., M.P.H.**

**Clinical Trials Specialist: Stacey Hurst, M.P.H., C.P.H.**

**Version 2.0**

**FINAL**

**March 07, 2012**

All questions concerning this protocol should be sent via e-mail to [impaact.teamp1094@fstrf.org](mailto:impaact.teamp1094@fstrf.org). Remember to include the subject’s PID when applicable. The appropriate team member will respond to questions via e-mail with a "cc" to [impaact.teamp1094@fstrf.org](mailto:impaact.teamp1094@fstrf.org). A response should generally be received within 24 hours (Monday - Friday).

For protocol registration questions, e‑mail [protocol@tech-res.com](mailto:protocol@tech-res.com) or call 301-897-1707. Protocol registration material can be sent electronically to [epr@tech-res.com](mailto:epr@tech-res.com) or via fax at 1-800-418-3544 or 301-897-1701. For EAE questions, e-mail [DAIDSRSCSafetyOffice@tech-res.com](mailto:DAIDSRSCSafetyOffice@tech-res.com) or call 1-800-537-9979 or 1-301-897-1709 or fax 1-800-275-7619 or 301-897-1710. For randomization or enrollment questions, contact the Data Management Center at 716-834-0900 x7301 or by email at [sdac.random.desk@fstrf.org](mailto:sdac.random.desk@fstrf.org)

| Protocol Chair  Allison L. Agwu, M.D., Sc.M.  Assistant Professor  Divisions of Adult & Pediatric Infectious Diseases  Johns Hopkins University  200 North Wolfe Street Rm 3145  Baltimore, MD 21287  Phone: (410) 614-3917  FAX: 410-614-1491  E-mail: [ageorg10@jhmi.edu](mailto:ageorg10@jhmi.edu)  Protocol Co-Vice Chairs  Jonathan M. Ellen, M.D. Professor and Vice Chair, Department of Pediatrics  Johns Hopkins School of Medicine Director, Department of Pediatrics Johns Hopkins Bayview Medical Center Mason F. Lord, Center Tower  5200 Eastern Ave, Ste 4200  Baltimore, MD 21224 Phone: (410) 550-4115  FAX: (410) 550-4153  E-mail: [jellen@jhmi.edu](mailto:jellen@jhmi.edu) | Protocol Co-Vice Chairs (Cont.)  Ann J. Melvin, M.D., M.P.H.  Associate Professor  Seattle Children’s Hospital  Division of Pediatric Infectious Disease **R-5441**  4800 Sand Point Way, N.E.  Seattle, WA 98105-0371  Phone: (206) 987-2535  FAX: (206) **987**-3890  E-mail: [ann.melvin@seattlechildrens.org](mailto:ann.melvin@seattlechildrens.org)  Division of AIDS Medical Officer  **Hans M.L. Spiegel, M.D., DrMedSc, DTMH, FAAP, Contractor**  **Medical Officer, PMPRB/Prevention Sciences Program,**  **DAIDS, NIAID, NIH**  **6700A Rockledge Drive**  **Suite 200, Room 42A 123**  **Bethesda, MD 20817**  **Phone: (301) 896-4071**  **FAX: (301) 896-0315**  **E-mail:** [**hans.spiegel@nih.gov**](mailto:hans.spiegel@nih.gov)  **Henry M. Jackson Foundation**  **for the Advancement of Military Medicine** |
| --- | --- |

| NICHD Medical Officer  George K. Siberry, M.D., M.P.H.  Ped Adol Maternal AIDS Branch  Eunice Kennedy Shriver National Institute of Child Health and Human Development 6100 Executive Blvd., Room 4B11H Bethesda, M.D. 20892  Phone: (301) 496-7350  FAX: (301) 496-8678  E-mail: [siberryg@mail.nih.gov](mailto:siberryg@mail.nih.gov)  Clinical Trials Specialist  **Stacey Hurst, MPH, CPH**  **IMPAACT Operations Center**  **Social & Scientific Systems, Inc. 1009 Slater Road, Suite 120 Durham NC 27703**  **Phone: (919) 287-4350**  **E-mail:** [**shurst@s-3.com**](mailto:shurst@s-3.com)  Protocol Statisticians  Vincent J. Carey, Ph.D.  Senior Statistician, Pediatric Division  Statistical & Data Analysis Center  Harvard School of Public Health  Building 3-505C, Channing Laboratory  677 Huntington Avenue  Boston, MA  02115-5821  Phone: (617) 525-2265  FAX: (617) 731-1541  E-mail: [stvjc@channing.harvard.edu](mailto:stvjc@channing.harvard.edu) | Protocol Statisticians (Cont.)  Meredith Warshaw, M.A.  Harvard School of Public Health Pediatric Section 651 Huntington Avenue, FXB-547 Boston, MA 02115  Phone: (617) 432-2481  FAX: (617) 432-3163  E-mail: [mwarshaw@sdac.harvard.edu](mailto:mwarshaw@sdac.harvard.edu)  Protocol Data Manager  Janice Hodge, R.N., B.S.  Frontier Science & Technology  Research Foundation  4033 Maple Road  Amherst, NY 14226-1056  Phone: (716) 834-0900x7269  FAX: (716) 834-8675  E-mail: [hodge.janice@fstrf.org](mailto:hodge.janice@fstrf.org)  Protocol Pharmacist  Ruth Ebiasah, Pharm.D., M.S., R.Ph.  Pharmaceutical Affairs Branch  Division of AIDS  6700-B Rockledge Drive, Room 4223  NIAID, National Institutes of Health  U.S. Department of Health and Human Services  Courier Zip Code: 20817  Bethesda, MD  Phone: (301) 402-0128  FAX: (301) 402-1506  E-mail: [ebiasahrp@niaid.nih.gov](mailto:ebiasahrp@niaid.nih.gov) |
| --- | --- |

| Protocol Virologist  Susan H. Eshleman, M.D., Ph.D.  Professor, Department of Pathology  Johns Hopkins University School of Medicine  Ross Building 646  720 Rutland Avenue  Baltimore, MD 21205  Phone: (410) 614-4734  FAX: (410) 502-9244  E-mail: [seshlem@jhmi.edu](mailto:seshlem@jhmi.edu)  Protocol Immunologist  Elizabeth (Betsy) J. McFarland, M.D.  Professor of Pediatrics  University of Colorado Denver  Pediatric Infectious Diseases  The Children's Hospital  13132 E. 16th Avenue, B055  Aurora, CO 80045  Phone: (303) 724-3447  FAX: (720) 777-7295  E-mail: [betsy.mcfarland@ucdenver.edu](mailto:betsy.mcfarland@ucdenver.edu)    Field Representative  Sandra Boyd, M.S.N, P.N.P.  Pediatric Nurse Practitioner St. Jude Children's Research Hospital  262 Danny Thomas Place, MS 600  Memphis, TN 38105  Phone: (901) 595-5059  FAX: (901) 595-5068  E-mail: [sandra.boyd@stjude.org](mailto:sandra.boyd@stjude.org) | Laboratory Data Coordinators  Alex Benns, B.S. Frontier Science and Technology Research Foundation 4033 Maple Rd Amherst, NY 14226 Phone: (716) 834-0900 Ext. 7464 FAX: (716) 833-0655 E-mail: [benns@fstrf.org](mailto:benns.alex@fstrf.org)  **IMPAACT Central Laboratory Specialist**  **Rachana Kshatriya, M.S., M (ASCP)**  **University of North Carolina**  **Mary Ellen Jones Bldg., Room 811**  **116 Manning Dr.**  **Chapel Hill, NC 27599. CB# 7290**  **Phone : (919) 966-1224**  **E-mail :** [**rackash@med.unc.edu**](mailto:rackash@med.unc.edu)  Ethicist  **Liza Dawson, Ph.D.**  **Branch Chief**  **Research Ethics Team Division of AIDS**  **6700B Rockledge Drive Room 4128 Bethesda MD 20892 United States of America**  **Phone: (301) 496-6179**  **E-mail:** [**dawsonl@niaid.nih.gov**](mailto:dawsonl@niaid.nih.gov) |
| --- | --- |

| Investigators  Andrew A. Wiznia, M.D.  P.I./Director, Pediatric HIV Services  Jacobi Medical Center/Family Based Services  1400 Pelham Parkway South  ACP room 5c15  Bronx, NY 10461  Phone: (718) 918-4664  FAX: (718) 918-4699  E-mail: [andrew.wiznia@einstein.yu.edu](mailto:andrew.wiznia@einstein.yu.edu)  Cynthia Rand, Ph.D.  Professor of Medicine  Johns Hopkins University  Dept of Medicine– Pulmonary  Asthma Center 4B-72 4940 Eastern Avenue Baltimore, MD 21224  Phone: (410) 550-0545  E-mail: [crand@jhmi.edu](mailto:crand@jhmi.edu)  Jacob Abadi, M.D.  Medical Director Pediatric/Adolescent HIV Services, Jacobi Medical Center  1400 Pelham Parkway So.  Building 5; Room 5C-15  Bronx, NY 10461  Phone: 718-918-4461  FAX: 718-198-4699  E-mail: [jacob.abadi@nbhn.net](mailto:jacob.abadi@nbhn.net)  **Lee Fairlie, M.D.**  **Wits Reproductive Health & HIV Research Institute (WRHI)**  **University of the Witwatersrand Hillbrow Health Precinct Hugh Solomon Building Corner Esselen Street, and Klein Street Hillbrow 2001**  **Republic of South Africa**  **Phone: +27827809997**  **E-mail:** [**lfairlie@wrhi.co.za**](mailto:lfairlie@wrhi.co.za) | Laboratory Technologist  Patricia Anthony, B.S., C.L.S.  University of Southern California  Maternal-Child Virology Research Lab  1801 East Marengo Street, GLB 1G8  Los Angeles, CA 90033  Phone: (323) 226-4161  Fax: (323) 226-4168  E-mail: [patricia.anthony@usc.edu](mailto:patricia.anthony@usc.edu)  Westat Clinical Research Associate  Scott P. Watson, R.N., B.S.  Westat Inc.  1441 W. Montgomery Ave.  Rockville, MD 20850  Phone: (415) 494-5575  Fax: (415) 859-9029  E-mail: [scottwatson@westat.com](mailto:scottwatson@westat.com) |
| --- | --- |

| NIAID INTERNATIONAL SITES | | |
| --- | --- | --- |
| 8051: Harriet Shezi Children’s Clinic, CRS  Old Potch Road Harriet Shezi Children’s Clinic, Hospital Street Soweto Johannesburg Gauteng  1863 South Africa  CRS Leader: Harry Moulrie  Phone: 27-11-9388189  Email: [harrym@witsecho.org.za](mailto:harrym@witsecho.org.za)  CRS Coordinator: Hermien Gous  Phone: 27-11-9388189  Email: [hermieng@witsecho.org.za](mailto:hermieng@witsecho.org.za) | 8355: Bhumibol Adulyadej Hospital, CRS  Phaholyothin Road  Saimai Bangkok, Ratchathewi  10220 Thailand  CRS Leader: Pacharee Kantipong  Phone: 66-5-3711300 ext. 1784  Email: [pachareek@hotmail.com](mailto:pachareek@hotmail.com)  CRS Coordinator: Angkana Sophon  Phone: 66-5-3711300  Email: [angkanaphpt@yahoo.com](mailto:angkanaphpt@yahoo.com) | 12702: Molepolole Prevention/Treatment Trials CRS (Molepolole)  Scottish Livingstone Hospital Gabaorone-Molepolole Road  Molepolole, Botswana  CRS Leader: Aida Asmelash  Phone: 267-3902671 Ext. 2128  Email: [aasmelash@bhop.org.bw](mailto:aasmelash@bhop.org.bw)  CRS Coordinator: Evans Moko  Phone: 267-5921013  Email: [emoko@bhp.org.bw](mailto:emoko@bhp.org.bw) |
| 8052: Soweto IMPAACT, CRS  Old Potch Road PHRU, Chris Hani Baragwanath Hospital 12th Floor,  Johannesburg Gauteng, South Africa  CRS Leader: Avy Violari  Phone: 27-11-9899707  Email: [violari@mweb.co.za](mailto:violari@mweb.co.za)  CRS Coordinator: Nasreen Abrahams  Phone: 27-11-9899742  Email: [abrahamsn@phru.co.za](mailto:abrahamsn@phru.co.za) | 8356: Chonburi Hospital, CRS  69 M. 2 Sukumvith Road  T. Ban Suan, Muang  Chonburi  20000 Thailand  CRS Leader: Chureeratana  Phone: 66-3-8931395  Email: [c.bowon@gmail.com](mailto:c.bowon@gmail.com)  CRS Coordinator: Ladda Argadamnuv  Phone: 66-3-8931000  Email: [ladda.argad@gmail.com](mailto:ladda.argad@gmail.com) | 12901: Kilimanjaro Christian Medical, CRS  3010 Sokoine Street  IDC Research Offices  Moshi, Tanzania  United Republic  CRS Leader: John A. Crump  Phone: 255-75-4610945  Email: [crump017@mc.duke.edu](mailto:crump017@mc.duke.edu)  CRS Coordinator: Suzanne Fiorillo  Phone: 255-78-3659498  Email: [suzanne.fiorillo@duke.edu](mailto:suzanne.fiorillo@duke.edu) |
| 8251: Siriraj Hospital Mahidol University, CRS  Building of Obstetrics and Gynecology,  floor 6 Siriraj Hospital Mahidol University  2 Prannok Road, Bangkoknoi  Bangkok, Thailand  Ratchathewi  CRS Leader: Kulkanya Chokephaibulkit  Phone: 66-2-4180545  CRS Coordinator: Kaewfa Intalapaporn Phone: 66-8-1620925  Email: [sikch@mahidol.ac.th](mailto:sikch@mahidol.ac.th) | 8950: Stellenbosch University, CRS  Franci van Ziji Drive  Hospital Ward J8  Parow, Cape Town  7505 South Africa  CRS Leader: Mark F. Cotton  Phone: 27-21-9384219  Email: [mcot@sun.ac.za](mailto:mcot@sun.ac.za)  CRS Coordinator: Joan Coetzee  Phone: 27-21-9384157  Email: [joan@sun.ac.za](mailto:joan@sun.ac.za) | 20101: Chiang Mai University Pediatrics-Obstetrics, CRS  Chiang Mai University  Research Institute for Health Sciences  110 Intavaroros Road, Chiang Mai, 50200 Thailand  CRS Leader: Virat Sirisanthana  Phone: 66-8-66705053  Email: [vsirisan@rihes.org](mailto:vsirisan@rihes.org)  CRS Coordinator: Chintana Khamrong  Phone: 66-5-3945051 Ext. 446  Email: [chintanak@rihes-cmu.org](mailto:chintanak@rihes-cmu.org) |
| 8352: Chiang Rai Regional Hospital, CRS  1039 Sathanpayaban Road Muang District  Chiangrai  57000, Thailand  CRS Leader: Pacharee Kantipong  Phone: 66-5-3711300 ext. 1784  Email: [pachareek@hotmail.com](mailto:pachareek@hotmail.com)  CRS Coordinator: Angkana Sophon  Phone: 66-5-3711300  Email: [angkanaphpt@yahoo.com](mailto:angkanaphpt@yahoo.com) | 12001: University of North Carolina Lilongwe, CRS  Kamuzu Central Hospital/Tidziwe Centre Mzimba Road Lilongwe, Malawi  CRS Leader: Francis Martinson  Phone: 265-8838388  Email: [fmartinson@unclilongwe.org.mw](mailto:fmartinson@unclilongwe.org.mw)  CRS Coordinator: Mina C. Hosseinipour  Phone: 265-1758938  Email: [minach@med.unc.edu](mailto:minach@med.unc.edu) | 20201: Durban Peadiatric HIV, CRS  Center Sydney and Francois Roads  Durban, KwaZulu-Natal  4001 South Africa  CRS Leader: Raziya Bobat  Phone: 27-27-312604355  Email: [bobat@ukzn.ac.za](mailto:bobat@ukzn.ac.za)  CRS Coordinator: Enbavani Pillay  Phone: 27-31-2604729  Email: [pillaye1@ukzn.ac.za](mailto:pillaye1@ukzn.ac.za) |
| 8353: Phayao Provincial Hospital, CRS 269 M. 11 Phaholyothin Road  T. Tom, Muang Phayao  56000, Thailand  CRS Leader: Guttiga Halue  Phone: 66-3-8931395  Email: [c.bowon@gmail.com](mailto:c.bowon@gmail.com)  CRS Coordinator: Ladda Argadamnuv  Phone: 66-3-8931000 Email: [ladda.argad@gmail.com](mailto:ladda.argad@gmail.com) | 12201: Hospital Nossa Senhora da Conceicao CRS  Avenida Francisco Trein, 596  Ambulatorio de Infectologia  Porto alegre, Rio Grande do Sul  91350-200 Brazil  CRS Leader: Breno R. Santos  Phone: 55-51-33415316  Email: [Breno@ghc.com.br](mailto:Breno@ghc.com.br)  CRS Coordinator: Rita D. Lira  Phone: 55-513-3572603  Email: [rita.lira@globo.com](mailto:rita.lira@globo.com) | 30273: George Clinic, CRS  George Health Centre  3DHE 11  Off Commonwealth Road  Lusaka, Zambia  CRS Leader: Benjamin Chi  Phone: 260-11-966859179  Email: [Benjamin.chi@cidrz.org](mailto:Benjamin.chi@cidrz.org)  CRS Coordinator: Felistas Mbewe  Phone: 260-966828341  Email: [pillaye1@ukzn.ac.za](mailto:pillaye1@ukzn.ac.za) |
| NIAID INTERNATIONAL SITES | | |
| 8354: Prapokklao Hospital, CRS  Prapokklao Hospital  38 Leibnern Road  Muang District, Chantaburi  22000, Thailand  CRS Leader: Prapap Yuthavisuthi  Phone: 66-3-9324975  Email: [yuthavisuthi@gmail.com](mailto:yuthavisuthi@gmail.com)  CRS Coordinator: Ubon Chanasit  Phone: 66-3-9324975 Email: [ubolchan@gmail.com](mailto:ubolchan@gmail.com) | 12701: Gaborone Prevention/Treatment Trials, CRS  Princess Marina Hospital Plot 1836, North Ring Road Gaborone, Botswana  CRS Leader: Anthony Ogwu  Phone: 267-3975999  Email: [anthony.ogwu@gmail.com](mailto:anthony.ogwu@gmail.com)  CRS Coordinator: Tebogo Kakhu  Phone: 267-3930335  Email: [tkakhu@bhp.org.bw](mailto:tkakhu@bhp.org.bw) | 31441: BJ Medical College  Jai Prakash Narayan Road  Pune, Maharashtra  411001, India  CRS Leader: Nishi Suryavanshi  Phone: 91-20-26052149  Email: [nishi@jhumitpune.com](mailto:nishi@jhumitpune.com)  CRS Coordinator: Nishi Suryavanshi  Phone: 91-20-26052149  Email: [nishi@jhumitpune.com](mailto:nishi@jhumitpune.com) |
| 30293: Makerere University-JHU Research Collaboration  Upper Mulago Hill Road  MUJHU Research House  Kampala, Uganda  CRS Leader: Philippa Musoke  Phone: 256-41-541044  Email: [pmusoke@mujhu.org](mailto:pmusoke@mujhu.org)  CRS Coordinator: Carolyne Onyango  Phone: 256-414-541044  Email: [ccarolonyango@mujhu.org](mailto:ccarolonyango@mujhu.org) | 30303: St. Mary’s, CRS St. Mary’s Clinic  Ingwe Road  Chitungwiza, Zimbabwe  CRS Leader: Tsungai Chipato  Phone: 263-4-2915421  Email: [tchipato@zol.co.zw](mailto:tchipato@zol.co.zw)  CRS Coordinator: Francis Jaji Phone: 263-912268516  Email: [francis@uz-ucsf.co.zw](mailto:francis@uz-ucsf.co.zw) |  |
| 30300: CAPRISA Umlazi, CRS  1358 Mangosuthu Highway  Umlazi, KwaZulu-Natal  4066, South Africa  CRS Leader: Dhayendre Moodley  Phone: 27-31-2604685  Email: [moodleyd1@ukzn.ac.za](mailto:moodleyd1@ukzn.ac.za)  CRS Coordinator: Vani Chetty  Phone: 27-31-2604680  Email: [chettyv1@ukzn.ac.za](mailto:chettyv1@ukzn.ac.za) | 30306: Seke North CRS  Jenje Road  Makoni Primary Health Care Clinic  Chitungwiza, Zimbabwe  CRS Leader: Lynda Stranix-Chibanda  Phone: 263-4-704890  Email: [lynda@uz-ucsf.co.zw](mailto:lynda@uz-ucsf.co.zw)  CRS Coordinator: Suzen Maonera  Phone: 263-912288160  Email: [suzen@uz-ucsf.co.zw](mailto:suzen@uz-ucsf.co.zw) |  |
| 30301: College of Medicine, JHU, CRS  Queen Elizabeth Central Hospital  Chipatala Avenue  Blantyre, Malawi  CRS Leader: Newton Kumwenda  Phone: 265-1875129  Email: [nkumwenda@jhu.medcol.mw](mailto:nkumwenda@jhu.medcol.mw)  CRS Coordinator: Leslie Degnam  Phone: 265-888-208609  Email: [aldegnan@jhu.medcol.mw](mailto:aldegnan@jhu.medcol.mw) | 30313: UZ-Parirenyatwa CRS  Cnr Josiah Tongagara/Mazowe Street  Parirenyatwa Annex  Harare, Zimbabwe  CRS Leader: James G. Hakim  Phone: 263-912225489  Email: [jhakim@mweb.co.zw](mailto:jhakim@mweb.co.zw)  CRS Coordinator: Jimijika Batani  Phone: 263-912272818 Email: [jbatani@uz-ucsf.co.zw](mailto:jbatani@uz-ucsf.co.zw) |  |

| NICHD INTERNATIONAL SITES | | |
| --- | --- | --- |
| 5071: Inst of Pediatrics Fed Univ Rio de Janeiro  Av. Vrigadeiro Trompowsky S/No.  S/N, llha do Fundao  Rio de Janeiro  21941-590, Brazil  Primary Investigator: Ricardo Hugo Oliveira  Phone: 55-21-562-61-48/49  Email: rh_oliveira@yahoo.com.br  Study Coordinator: Maria Chermont  Phone: 55-21-562-61-48/49  Email: macher@rio.com.br | 5074: Universidade de Sao Paulo  Av. Bandeirantes 3900  Ribeirao Preto, Sao Paulo  CEP 14049-900, Brazil  Primary Investigator: Marisa Mussi-Pinhata  Phone: 55-16-3602-2479  Email: mmmpinha@fmrp.usp.br  Study Coordinator: Marcia de Lima Issac  Phone: 55-16-3621-7692  Email: mdlisaac@hotmail.com | 5097: Hospital Geral De Nova Igaucu Brazil  Av. Henrique Duque Estrada Mayer  Alto da Posse_Nova Iguac, Rio de Janeiro  26030-380, Brazil  Primary Investigator: Jose Henrique  Phone: 55-21-2667-3022  Email: gisely.falco@gmail.com  Study Coordinator: Gisely Goncalves  Phone: 55-21-2667-6059  Email: luamsmarins@gmail.com |
| 5072: Hospital dos Servidores Rio de Janeiro  Rua Sacadura Cabral, 178  Anexo IV, 4 Andar  CEP 20221-161, Brazil  Primary Investigator: Esau Custodio Joao  Phone: 55-21-535-0493  Email: esau@uninet.com.br  Study Coordinator: Leon Claude Sidi  Phone: 55-212-203-2013  Email: leon@diphse.com.br | 5075: Inst de Infectologia Emilio Ribas Sao Paulo Brazil  Av. Dr. Arnaldo  165-6o Andar-Sala 620  Sao Paula, Sao Paula  01246-900, Brazil  Primary Investigator: Marinella Della Negra  Phone: 55-11-3085-0295  Email: aacphiv@uol.com.br  Study Coordinator: Wladimir  Phone: 55-11-3085-0295  Email: aacphiv@uol.com.br | 5098: Hospital Santa Casa Porto Alegre Brazil  Mario Totta rua Prof Annes Dias 285  1 Andar, RS  90020-090, Brazil  Primary Investigator: Regis Kreitchmann  Phone: 55-51-3214-8008  Email: regis.kr@terra.com.br  Study Coordinator: Debora Coelho  Phone: 55-51-3331-1765  Email: deby.nh@terra.com.br |
| 5073: SOM Federal University Minas Gerais Brazil  Av. Alfredo Balena 90-4^o^ Andar  Belo Horizonte, Minas Gerais  CEP 30130-100, Brazil  Primary Investigator: Jorge Pinto  Phone: 55-31-3248-9822  Email: jpinto@medicina.ufmg.br  Study Coordinator: Fabiana Kakehasi  Phone: 55-31-3248-9822  Email: kakehasi@medicina.ufmg.br | 5082: Hospital General de Agudos Buenos Aires Argentina  Urquiza 609, Pabellon de Clinica, 2 Pisc  Buenos Aires, C1221ADC, Argentina  Primary Investigator: Marcelo Losso  Phone: 5411-4931-5252/4127-276  Email: mlosso@hivramos.org.ar  Study Coordinator: Silvina Ivalo  Phone: 5411-4931-5252/4127-276  Email: sivalo@hivramos.org.ar |  |

TABLE OF CONTENTS

[**Summary of Changes** 11](#__RefHeading___Toc315096441)

[List of Commonly Used Abbreviations and Definitions 15](#__RefHeading___Toc315096442)

[SCHEMA ………………………………………………………………………………..17](#__RefHeading___Toc315096443)

[1.0 INTRODUCTION 21](#__RefHeading___Toc315096444)

[1.1 Background ……………………………………………………………...21](#__RefHeading___Toc315096445)

[1.2 Rationale ……………………………………………………………...22](#__RefHeading___Toc315096446)

[2.0 STUDY OBJECTIVES 39](#__RefHeading___Toc315096447)

[2.1 Objectives for Step 1- Randomized Study (enrollment to **Week 28**) 39](#__RefHeading___Toc315096448)

[2.2 Objectives for Step 2 (Weeks **28** to **52**) 40](#__RefHeading___Toc315096449)

[3.0 STUDY DESIGN 41](#__RefHeading___Toc315096450)

[3.1 Screening Log 44](#__RefHeading___Toc315096451)

[4.0 SELECTION AND ENROLLMENT OF SUBJECTS 45](#__RefHeading___Toc315096452)

[4.1 Step 1 - Inclusion Criteria 45](#__RefHeading___Toc315096453)

[4.2 Step 1 - Exclusion Criteria 47](#__RefHeading___Toc315096454)

[4.3 Step 2 - Inclusion Criteria 49](#__RefHeading___Toc315096455)

[4.4 Concomitant Medication Guidelines 49](#__RefHeading___Toc315096456)

[4.5 Enrollment Procedures 50](#__RefHeading___Toc315096457)

[4.6 Co-enrollment Procedures 51](#__RefHeading___Toc315096458)

[5.0 STUDY TREATMENT 52](#__RefHeading___Toc315096459)

[5.1 Drug Regimens, Administration and Duration 52](#__RefHeading___Toc315096460)

[5.2 Drug Supply, Distribution and Pharmacy 53](#__RefHeading___Toc315096461)

[6.0 SUBJECT MANAGEMENT 53](#__RefHeading___Toc315096462)

[6.1 Toxicity Management 53](#__RefHeading___Toc315096463)

[6.2 Subject Management 54](#__RefHeading___Toc315096464)

[6.3 Criteria for Stopping Randomized Treatment 55](#__RefHeading___Toc315096465)

[6.4 Criteria for Earlier (before **week 28**) Completion of Step 1 56](#__RefHeading___Toc315096466)

[6.5 Criteria for Study Discontinuation 56](#__RefHeading___Toc315096467)

[7.0 EXPEDITED ADVERSE EVENT REPORTING 56](#__RefHeading___Toc315096468)

[7.1 Adverse Event Reporting to DAIDS 56](#__RefHeading___Toc315096469)

[7.2 Reporting Requirements for this Study 56](#__RefHeading___Toc315096470)

[7.3 Grading Severity of Events 57](#__RefHeading___Toc315096471)

[7.4 Expedited AE Reporting Period 57](#__RefHeading___Toc315096472)

[8.0 STATISTICAL CONSIDERATIONS 57](#__RefHeading___Toc315096473)

[8.1 General Design Issues 57](#__RefHeading___Toc315096474)

[8.2 Outcome Measures 58](#__RefHeading___Toc315096475)

[8.3 Randomization and Stratification 59](#__RefHeading___Toc315096476)

[8.4 Sample Size and Accrual 59](#__RefHeading___Toc315096477)

[8.5 Monitoring ……………………………………………………………...60](#__RefHeading___Toc315096478)

[8.6 Analyses ……………………………………………………………...62](#__RefHeading___Toc315096479)

[9.0 HUMAN SUBJECTS 66](#__RefHeading___Toc315096480)

[9.1 Institutional Review Board and Informed Consent 66](#__RefHeading___Toc315096481)

[9.2 Subject Confidentiality 66](#__RefHeading___Toc315096482)

[9.3 Study Discontinuation 67](#__RefHeading___Toc315096484)

[10.0 PUBLICATION OF RESEARCH FINDINGS 67](#__RefHeading___Toc315096485)

[11.0 BIOHAZARD CONTAINMENT 68](#__RefHeading___Toc315096486)

[12.0 REFERENCES 69](#__RefHeading___Toc315096487)

APPENDICES

1. SCHEDULE OF EVALUATIONS
2. CDC CATEGORY C HIV CLASSIFICATION SYSTEM (FOR CHILDREN <13 YEARS OF AGE)
3. CDC CATEGORY C HIV CLASSIFICATION SYSTEM (FOR ADOLESCENTS AND YOUNG ADULTS ≥13 YEARS OF AGE)
4. BLOOD VOLUME COLLECTION IN SMALL CHILDREN
5. DAIDS SAMPLE INFORMED CONSENT TEMPLATE
6. FACT SHEET and TEMPLATE CONSENT FORM for Specimen Storage at Repositories funded by the National Institute of Child Health and Human Development (NICHD)- PARENT FACT SHEET
7. FACT SHEET and TEMPLATE CONSENT FORM for Specimen Storage at the Repository of the National Institute of Child Health and Human Development (NICHD)- YOUTH FACT SHEET

**Summary of Changes**

**P1094: Evaluation of 3TC or FTC Monotherapy Compared to Continuing HAART as a Bridging Antiretroviral Strategy in Persistently Non-Adherent Children, Adolescents, and Young Adults who are Failing HAART and have the M184V Resistance Mutation, Version 2.0, dated March 01, 2012.**

**All changes in this version appear in boldface type. Major changes are listed below. Editorial changes, including corrections of typographical errors and other changes required to update information not affecting regulatory issues or the Sample Informed Consent may also be included. Information from Letter of Amendment #1 (May 27, 2011), Clarification Memo #1 (February 1, 2011), and Clarification Memo #2 (July 27, 2011) are included with updates as appropriate based on other changes in this amendment.**

1. **The cover of the protocol is updated with the current scientific committee affiliation and team member information.**
2. **The team roster is updated with current team member information.**
3. **Throughout the protocol:**
   - **References to Week 8 are removed. (This study visit is removed from the Schedule of Evaluations.)**
   - **References to Week 18 are replaced with Week 20. (The Week 18 study visit is now the Week 20 study visit.)**
   - **References to Week 24 are replaced with Week 28 and references to the first 24 weeks of the study are replaced with 28 weeks. (The Week 24 study visit is now the Week 28 study visit.)**
   - **References to Week 36 are replaced with Week 40 and references to 36 weeks in relation to the study period are replaced with 40 weeks. (The Week 36 study visit is now the Week 40 study visit.)**
   - **References to Week 48 are replaced with Week 52 and references to 48 weeks in relation to the study period are replaced with 52 weeks. (The Week 48 study visit is now the Week 52 study visit.)**
4. **Schema (Design) and Section 3.0 (Study Design Diagram): The last sentence of the second paragraph under the flow chart has been updated for consistency with the study design.**
5. **Schema (Population) and Section 3.0: The phrase ‘and are persistently non-adherent’ has been added to the description of the study population.**
6. **Schema (Primary Objective) and Section 2.111: From Version 1.0 the primary objective read:**

**“To compare immunologic deterioration during a 24 week “bridging” treatment strategy of 3TC or FTC monotherapy vs. continuing HAART in HIV-infected children, adolescents, and young adults with virologic failure who are likely to be non-adherent to an optimized HAART regimen (due to problems related to adherence, tolerability, or toxicity). Indicators of immunologic deterioration will be a ≥ 30% decline in absolute CD4+ T cell count, or a drop in CD4+ T cell count to less than 200 cells/mm^3^, or development of CDC class C events.”**

**The primary objective is revised to delete the reference to a drop in CD4+ T cell count to less than 200 cells/mm^3^.**

1. **Schema (Step 2 Secondary Objective vi.), Section 2.216, and Section 8.616 have been updated for consistency and clarity.**
2. **Schema (Population Section), Section 3.0, and Section 4.13:**
   - **The definition for failure of the current drug regimen is revised to be a confirmed* (*two viral loads from two different draws) viral load ≥400 copies/mL at least 2 months after initiating the current regimen and at screening.**
3. **Section 1.23: Information to strengthen the justification for the enrollment of children who are younger than adolescents has been added to the second paragraph.**
4. **Section 1.262: The second sentence in the second paragraph is re-worded for clarity.**
5. **Section 1.27 is added to provide a summary of the study rationale.**
6. **Section 3.0: Number of evaluable subjects corrected for consistency with Section 8.52.**
7. **Section 3.1: The fifth sentence in the first paragraph is updated to provide additional examples of CD4 T+ cell count ranges.**
8. **Section 4.13: This inclusion criterion is revised to decrease the amount of time required for potential subjects to demonstrate failure on the current HAART regimen (2 months instead of 6 months)**.
9. **Section 4.14: This inclusion criterion is revised to lower the CD4+ T cell count threshold from 250 cells/mm^3^ to 100 cells/mm^3^.**
10. **Section 4.21: Additional language is added to this exclusion criterion to indicate that subjects with known active hepatitis B disease are also excluded.**
11. **Section 5.12: Since entry into Step 2 can occur prior to week 28 (up to week 34), the last sentence in the third paragraph is modified.**
12. **Section 6.2: Subsection *Step 1*, the parenthetical phrase in the last paragraph now reads “**(**any time after entry up to week 34)”.**
13. **Section 6.3:**
    - **The criteria for stopping randomized treatment is revised to delete the reference to CD4+ T cell count < 200 cells/mm^3^.**
    - **The criterion regarding a % decrease in CD4+ T cell count from step entry is updated to indicate that it is ≥ 30% (instead of > 30%) and to include information regarding confirmatory testing.**
    - **The term ‘stage’ has been replaced with ‘step’ for consistency with protocol terminology**
14. **Section 7.2: Rilpivirine has been added as a study agent which requires expedited reporting.**
15. **Section 8.21: From Version 1.0, the primary outcome measures section read:**

**“Immunologic deterioration will be declared for a subject if any one of the following conditions is observed within the first 24 weeks:**

- **≥ 30% decline in absolute CD4+ T cell count,**
- **or a drop in CD4+ T cell count to less than 200 cells/mm^3^, or development of CDC class C events.”**

**The primary outcome measures section is revised to delete the reference to a drop in CD4+ T cell count to less than 200 cells/mm^3^.**

1. **Accrual information in section 8.4 has been updated.**
2. **The paragraph on accrual monitoring in section 8.5 has been updated.**
3. **Section 8.61: The Primary Objective (immunologic failure) is revised to delete the reference to a drop in CD4+ T cell count to less than 200 cells/mm^3^.**
4. **Appendix I (Schedule of Evaluations):**
   - **The visit windows are increased.**

- **The column entitled, “*Change in treatment regimen not occurring at Step 2 entry*” is changed to “*Change in treatment regimen occurring before week 28*”.**
- **Information has been added to the boxes for Week 40 and Week 52 to clarify the timing of these visits for subjects who enter Step 2 before week 28.**
- **To ensure that HIV phenotyping with replication capacity is collected in subjects who discontinue treatment prior to week 28, HIV phenotyping with replication capacity [6 mL] is added under the “*Change in treatment regimen occurring before week 28*” column. The total blood volume for this visit is also revised accordingly. This evaluation also includes a footnote (#20) that states that HIV phenotyping with replication capacity should not be repeated if it was collected within the past 14 days. To accommodate this new footnote, the footnotes are renumbered accordingly.**
- **Under the Week 52 column, the superscript ‘11’ associated with 6 mL (for *HIV phenotyping with replication capacity*) is deleted since it is in excess.**
- **Footnote #8: Information regarding the stopping criterion for viral load is added.**
- **Footnote #9: Information has been added to indicate that genotyping should be performed to document presence of the M184V mutation at screening for U.S. sites, if not done previously, with a note to clarify that the domestic sites will have to pay for the screening genotype.**
- **Footnote #14: The reference to a drop in CD4+ T cell count to less than 200 cells/mm^3^ is deleted. Information for the stopping criterion for a drop in CD4+ T cell count is added.**
- **Footnotes #15 and #16 are updated to update laboratory shipping and processing information.**
- **The last sentence in footnote #17 is modified as follows: “*Entry into Step 2 can occur any time after entry up to week 34.*”**
- **Footnote #18: Study procedures for subjects who discontinue study-assigned treatment early are clarified.**

1. **Appendix IV: This section is updated to reflect the most recent National Institutes of Health (NIH) guidelines for blood collection in small children.**
2. **Appendix V (Sample Informed Consent Form):**

- **Changes to the visit schedule have been made throughout the Sample Informed Consent Form - (Week 8 is deleted; Week 18 is now Week 20; Week 24 is now Week 28, Week 36 is now Week 40; Week 48 is now Week 52).**
- **The section, *WHAT DO I/DOES MY CHILD HAVE TO DO IF I AM HE/SHE IS IN THIS STUDY?*, is updated to indicate that subjects will come to the clinic for 10 follow-up visits (instead of 11).**
- **In the section, *WHAT DO I/DOES MY CHILD HAVE TO DO IF I AM HE/SHE IS IN THIS STUDY?*, in the schedule overview table, the column entitled “*Change in treatment regimen not occurring at Step 2 entry*” is revised to read as follows: “*Change in treatment regimen occurring before week 28*”.**
- **In the section, Step 2 *Entry*, the following phrase is deleted from the first paragraph, “*between week 24 and week 30*” and replaced with “*any time after entry up to week 34*”. In addition, some wording of the paragraph was modified to improve readability. Therefore, the revised paragraph reads as follows: “*You/your child will enter Step 2 (any time after entry up to week 34) once blood test results are available to determine if your/your child’s HIV has changed. The test results will help you and your/your child’s doctor decide if you/your child should change treatment regimens. At this Step 2 entry visit, the following will be done:”***
- **To account for the potential blood volume increase at the *Change in Treatment Visit*, the statement regarding blood volume is modified and reads as follows:**

**“You/your child may have about 7 teaspoons (30.7 mL – 36.7 mL) of blood drawn to:”**

- ***WHAT ARE THE RISKS OF THE STUDY?* Section, Monotherapy Subsection: the following sentence has been added, ‘It is also possible that by discontinuing HAART you will increase the risk of transmission of HIV to a non-infected sexual partner.”**
- ***WHAT ABOUT CONFIDENTIALITY?* Section: Information about ClinicalTrials.gov has been added.**
- ***WHAT ABOUT CONFIDENTIALITY?* Section, 4^th^ paragraph: The end of this paragraph states, “…under the following circumstances.” To clarify, the list of circumstances, if applicable, is to be provided by sites to indicate the conditions under which voluntary disclosure will be made per site standards.**

# List of Commonly Used Abbreviations and Definitions

| 3TC | Lamivudine |
| --- | --- |
|  |  |
| ABC | Abacavir |
| ADI | AIDS-defining illness |
| AE | Adverse event |
| AIDS | Acquired immune deficiency syndrome |
| ALT | Alanine transaminase |
| ARV | antiretroviral |
| ART | Antiretroviral therapy |
| AST | aspartate aminotransferase |
| ATV | Atazanavir |
| AZT | Zidovudine |
|  |  |
| CCR5 | Chemokine receptor type 5 |
| CDC | Centers for Disease Control and Prevention |
| CFR | Code of Federal Regulations |
| CI | Confidence interval |
| CRF | Case report form |
|  |  |
| d4T | Stavudine |
| DAERS | DAIDS Adverse Experience Reporting System |
| DAIDS | Division of AIDS |
| DSMB | Data and Safety Monitoring Board |
| DHHS | Department of Health and Human Services |
| DMC | Data Management Center |
| DRV | Darunavir |
|  |  |
| EAE | Expedited adverse events |
| EC | Ethics committee |
| EFV | Efavirenz |
|  |  |
| FDA | Food and Drug Administration |
| FTC | emtricitabine |
|  |  |
| GALT | Gut-associated lymphoid tissue |
| G-CSF | Granulocyte colony-stimulating factor |
| GM-CSF | Granulocyte-macrophage colony-stimulating factor |
|  |  |
| HAART | Highly Active Antiretroviral Therapy |
| HDL | High-density lipoprotein |
| HHS | Health and Human Services |
| HIV | Human Immunodeficiency Virus |
|  |  |
| ICAAC | Interscience Conference on Antimicrobial Agents and Chemotherapy |
| ICF | Informed consent form |
| IL-2 | Interleukin-2 |
| IMPAACT | International Maternal Pediatric Adolescent AIDS Clinical Trials Group |
| INH | Isoniazid |
| IQR | interquartile range |
| IRB | Institutional review board |
| IUD | Intrauterine device |
|  |  |
| LAR | legally authorized representative |
| LDL | Low-density lipoprotein |
| LPS | lipopolysaccharide |
|  |  |
| NIAID | National Institute of Allergy and Infectious Diseases |
| NICHD | The Eunice Kennedy Shriver National Institute of Child Health and Human Development |
| NIH | National Institutes of Health |
| NNRTI | Non-Nucleoside reverse transcriptase inhibitor |
| NRTI | Nucleoside reverse transcriptase inhibitor |
|  |  |
| OHRP | Office for Human Research Protections, DHHS |
|  |  |
| PI | Protease inhibitor |
| PRO | Protocol Registration Office |
| PTI | Partial treatment interruption |
|  |  |
| RCT | Randomized clinical trial |
| RE | Regulatory entity |
| RSC | Regulatory Support Center |
| RTV | Ritonavir |
| RT-based | Reverse transcriptase based |
|  |  |
| SAE | Serious Adverse Event/Serious Adverse Experience |
| SDAC | Statistical and Data Analysis Center |
| SIP | Site implementation plan |
| SMART | Strategies for Management of Anti-retroviral Therapies |
| SUSAR | Suspected, Unexpected, Serious Adverse Reactions |
|  |  |
| TB | Tuberculosis |
| TDF | Tenofovir disoproxil fumarate |
| TI | Treatment interruption |
|  |  |
| US | United States |
|  |  |
| VL | Viral load |
|  |  |
| WHO | World Health Organization |

SCHEMA

Evaluation of 3TC or FTC Monotherapy Compared to Continuing HAART as a Bridging antiretroviral Strategy in persistently non-adherent Children, Adolescents, and young adults who are failing haart and have the M184V Resistance Mutation

DESIGN: Multi-center, Phase IV, randomized, controlled, comparative trial of 3TC or FTC monotherapy vs. continuing non-suppressive HAART for **28** weeks

Step 2: > Week **28**

Week 0: Subjects whose providers are not planning to stop their HAART in the setting of virologic failure are randomized to 3TC or FTC Bridging vs. Continuing Non-suppressive HAART. Subjects commit to at least 6 months on the randomized study regimen and only switch if they reach clinical/primary endpoints as defined in the protocol.

Week **28**: completion of randomized study. At this time, the primary outcomes will be compared between the two study arms. **Subjects should** continue to step 2.

Step 2 (Week >**28 to 52**): Subjects may: (a) continue randomized treatment, (b) begin a new HAART regimen , if the clinical team including the subject, and or caregiver (as appropriate) determine that it is clinically necessary or adherence is likely, or (c) discontinue study treatment, but remain on study follow-up. This decision will be made by the subject and their clinical team and not the study team.

Note: Beyond week **52**, follow-up of outcomes on new HAART regimen will be done through data collected in P1074 (highly encouraged co-enrollment).

*The decision will be made by the subject and their clinical team and not the study team.

Begin new

HAART

regimen*

Continue randomized treatment*

Discontinue therapy, but remain on

follow up*

END OF STUDY (assessment of objective for Step 2)

HIV-Infected Children, Adolescents, and Young Adults Failing HAART with the M184V Mutation and Persistently Non-Adherent

Arm A: Bridging Therapy

Continue Non-suppressive HAART (x**28** weeks) 172 subjects

Primary endpoint. Subsequent ART as determined by clinical care

Primary endpoint. Subsequent ART as determined by clinical care

Continue randomized treatment*

Arm B: Bridging Therapy

3TC or FTC Monotherapy

(x**28** weeks) 172 subjects

344 Subjects Randomized

Week **28**

Step 1: Week 0

Begin new

HAART regimen*

Week **52**

Discontinue therapy, but remain on

follow up*

SAMPLE SIZE: 344 subjects (172 subjects/arm).

POPULATION: HIV-infected subjects ≥8 to <25 years of age who have documentation of the M184V HIV resistance mutation and are failing their current HAART regimen **and are persistently non-adherent**. Failure of their current regimen is defined as a confirmed*** (** Two viral loads from two different draws*) viral load ≥400 copies/mL at least **2** months after initiating the current regimen and at screening.

Note: The current HAART regimen must include at least three non-NNRTI drugs from at least 2 classes (i.e., PI, NRTI, integrase inhibitor, CCR5 antagonist, fusion inhibitor), OR at least 3 NRTIs that constitute a HAART regimen (e.g., ABC/ZDV/3TC).

STRATIFICATION: Subjects will be stratified by baseline CD4+ T cell count (<400 cells/mm^3^ versus ≥400 cells/mm^3^)

REGIMEN: In Step 1, subjects will be randomized to continue their non-suppressive HAART regimen (Arm A) OR to receive 3TC or FTC monotherapy (Arm B); (the choice of 3TC or FTC will be left to the provider)

TREATMENT DURATION: In Step 1, subjects will receive 3TC or FTC monotherapy OR will continue their current non-suppressive HAART regimen for **28** weeks, followed by a follow-up period of 24 weeks (Step 2) when subjects may: (a) continue randomized treatment, (b) begin a new HAART regimen, if the clinical team including the subject, and or caregiver (as appropriate) determine that it is clinically necessary or adherence is likely, or (c) discontinue study treatment, but remain on study follow-up. This decision will be made by the subject and their clinical team and not the study team.

STUDY DURATION: **52** weeks: **28** weeks (Step 1-randomized study); 24 weeks (Step 2).

OBJECTIVES:

1. Objectives for Step 1- Randomized Study (Enrollment to Week **28**)

Primary Objective

1. To compare immunologic deterioration during a **28** week “bridging” treatment strategy of 3TC or FTC monotherapy vs. continuing HAART in HIV-infected children, adolescents, and young adults with virologic failure who are likely to be non-adherent to an optimized HAART regimen (due to problems related to adherence, tolerability, or toxicity). Indicators of immunologic deterioration will be a ≥ 30% decline in absolute CD4+ T cell count or development of CDC class C events.

Secondary Objectives

1. To compare the change in genotypic HIV drug resistance from baseline to week **28** in the two study arms.
2. To compare the slope of CD4+ T cell count and percent over **28** weeks between the two study arms.
3. To examine factors (e.g., demographic, immunologic, and virologic) associated with immunologic deterioration.
4. To compare the change in HIV viral load and CD4+ T cell count from baseline to **week 28** in the two study arms.
5. To compare changes in immune activation and cardiovascular risk factors (e.g., CD8+/CD38+/HLA-DR+T lymphocytes, hsCRP, LDL, and HDL cholesterol) from baseline to **week 28** in the two study arms.
6. To compare the rates of adverse events in the two study arms.
7. To compare adverse clinical outcomes (e.g., bacterial infections, hospitalizations, HIV-related and non-HIV-related morbidity) in the two study arms.
8. To compare adherence for subjects in the two study arms, as measured by 3-day recall.
9. To compare the change in phenotypic HIV drug resistance and HIV replication capacity from baseline to **week 28** in the two study arms in a subset of participants.
10. Objectives for Step 2 (Weeks **28** to **52**)

Secondary Objectives

1. To compare the proportion of subjects in the two originally assigned treatment arms who do not experience immunologic failure at **40** and **52** weeks and to identify factors (e.g., demographic, immunologic, and virologic) predictive of experiencing immunologic failure.
2. To compare CD4+ T cell count changes from **28** weeks to **40** and **52** weeks in subjects, in the two originally assigned treatment arms.
3. To compare the mean change in genotypic HIV drug resistance in subjects in the two originally assigned treatment arms.
4. To compare adherence to the study regimens at **40** and **52** weeks among subjects in the two originally assigned treatment arms.
5. To compare changes in immune activation and cardiovascular risk factors (e.g., CD8+/CD38+/HLA-DR+T lymphocytes, hsCRP, LDL, and HDL cholesterol) from baseline to **52** weeks in the two originally assigned treatment arms.
6. To compare the rate**s** of virologic suppression and CD4+ T cell count recovery among subjects in the two originally assigned treatment arms 24 weeks after **starting Step 2**.
7. To compare adherence to a new HAART regimen among subjects in the two originally assigned treatment arms.
8. INTRODUCTION
   1. Background

Currently, there is no clear consensus for managing virologic failure. The Department of Health and Human Services (DHHS) guidelines present options, including: (1) aggressively changing regimens for any repeated, detectable viremia (two consecutive viral load (VL) values >50 copies/ml (c/mL) after suppression to <50 c/mL), or (2) continuing regimens, allowing detectable viremia up to an arbitrary level of 1,000-5,000 c/mL on a given regimen, with the caveat that one should consider changing regimens sooner to avoid antiretroviral (ARV) drug resistance (1) . Both approaches are to be accompanied by intensive counseling on adherence and medication readiness. However, in the setting of non-adherence, both strategies may be associated with negative consequences. The first strategy can lead to development of ARV drug resistance on the next regimen due to continued non-adherence. The second strategy can lead to the accumulation of additional resistance mutations to the failed regimen, which could reduce future treatment options through cross-resistance to ARV drugs used in subsequent regimens. Generally, failure of non-nucleoside reverse transcriptase inhibitor (NNRTI)-based therapy due to non-adherence is associated with high rates of NNRTI resistance, while failure of protease inhibitor (PI)-based therapy due to non-adherence carries a much lower risk of PI resistance (2;3). In the setting of incomplete adherence and virologic failure despite adherence education and interventions, an optimal strategy would be one that effectively bridges the period between the cessation of the failing regimen of highly active ARV therapy (HAART) and initiation of a new HAART regimen. This would provide time for interventions to improve adherence to be effective while minimizing accumulation of additional drug resistance mutations. Lamivudine (3TC) or emtricitabine (FTC) monotherapy may be just such a bridging strategy, as there is evidence that 3TC or FTC monotherapy may slow immunologic and clinical progression and may reduce the risk of development of further resistance mutations, while providing time to improve adherence.

A survey of IMPAACT clinical sites completed in October 2009 suggests

that the majority of providers struggle with patients who are in need of HAART by guidelines, but have not had durable virologic suppression on HAART primarily due to non-adherence. The survey also suggested that the use of 3TC/FTC monotherapy as an approach to bridging patients from a failing HAART to a new regimen, though not widely used, is considered by many clinicians to be a potentially effective strategy and acceptable as an arm in a clinical trial. Given the compelling need for an effective bridging strategy, the limited evidence for the safety and efficacy of this bridging regimen, and the high level of acceptability of studying 3TC or FTC monotherapy as an effective alternative, we propose to conduct a randomized clinical trial (RCT) comparing use of 3TC or FTC monotherapy as a short-term bridging regimen vs. continuation of non-suppressive HAART in non-adherent subjects.

The proposed study may be especially relevant to the treatment of adolescents. Adolescence is characterized as a developmental period of limited abstract reasoning, limited future orientation, and limited experience with negative consequences of behaviors. These limitations may be greater in perinatally-infected adolescents because of the neurotropic effects of HIV. Differences in adolescent and adult behavior, and differences in provider attitudes towards these two patient groups, are likely to result in differences in the management of non-adherence in adolescent versus adult patients. In the case of adolescents, providers may find it more acceptable to delay start of a new HAART regimen until they are reassured that adherence has been improved. In contrast, adult providers may act more quickly to offer a new regimen and place responsibility for improving adherence on the patient.

- 1. Rationale
     1. Treatment Strategies

The optimal goals of antiretroviral therapy (ART) are to suppress viral replication, preserve immune function, prolong survival, and maintain quality of life. In the United States, many perinatally infected children and adolescents have been exposed to sequential ARV monotherapy and combination regimens that have resulted in incomplete viral suppression and selection for drug-resistant viral subpopulations. This resistance, coupled with a paucity of newer and more potent agents, severely compromises the clinician’s ability to design effective and tolerable regimens. A change in the ARV regimen is usually prompted by disease progression, toxicity, intolerance, or the availability of a better ARV combination. If ART has failed, a comprehensive evaluation of the child’s treatment that addresses adherence, previous ARV exposures, pharmacologic issues (pharmacokinetics, pharmacodynamics, drug interactions), and the results of genotypic/phenotypic resistance testing is essential. Intermittent “blips” of viremia are commonly observed among patients with long-term viral suppression; the implications of these episodes are still unknown. In contrast, persistent low-level viremia eventually will select for more drug resistance and may limit future treatment options (4). Although the theoretic goal of ART is to completely abolish viral replication in the host, we now know that viral evolution can continue even in those who are fully suppressed as defined by conventional viral load assays (5). Many patients are far from a “perfect” or near-perfect level of virologic control and face an uncertain future. Studies of the pathogenesis of drug-resistant HIV may shed more light on the concept of a “tolerable” level of viremia, one that would be host- and virus-specific and would result in little or no disease progression (6). Several strategies have been proposed to address treatment failure in ARV-experienced patients.

Full treatment interruptions offer the potential benefits of decreased toxicity and costs, a better quality of life, and theoretic boosting of HIV-specific immune responses (auto-vaccination) (7). However, discontinuing all therapy may result in a rapid increase in viral load and a rapid drop in CD4+ T cell levels, which may not return to baseline when treatment is reinstituted (8). In addition, recent data suggest that episodic ART, when compared with continuous ART, may be associated with increased short-term risk of HIV disease progression and death (SMART Study).

A strategy termed “partial treatment interruption” (PTI) consists of selective discontinuation of only one class of therapy (9). In a recent report of perinatally HIV-infected children and adolescents who had persistent detectable plasma viremia, and elected to stop the PI component of their regimen while maintaining treatment with two or more RTIs (typically zidovudine and lamivudine or stavudine and lamivudine combination); there was no CDC-defined disease progression or significant change in viral load in subjects treated for up to 96 weeks (10). This result suggests that simplification of therapy could provide a temporizing option for patients intolerant of or failing HAART. Furthermore, in a subgroup of these children, a longitudinal study of HIV-specific cellular immune responses showed that immediate to PTI, HIV-specific CD4+ T cell and CD8 T cell responses significantly increased, while activation levels remained unchanged. The authors concluded that HIV-specific cellular immune responses are boosted in children who have interrupted PI-based therapy (11). With respect to resistance, despite the absence of PI drug pressure, PI–associated mutations persisted over a long period of time. Deleterious NRTI mutations such as Q151M, K65R, or 69 Insertion Complex did not accumulate despite prolonged NRTI pressure however, this is a risk. The authors speculate that PI mutations may persist because of their linkage to NRTI mutations on the same viral genome (12).

In summary, simplification of therapy to a PTI regimen may provide a safe, effective and durable option in patients failing or intolerant of HAART. Moreover, as a temporizing measure, this therapeutic approach may serve as a bridging strategy that allows for medical stability until new drugs become available or educational/adherence interventions have been effective. The goal of the study is to inform providers who find themselves facing a common clinical scenario (patient in need of HAART, but with continued non-adherence and virologic non-suppression) on interim ARV management while waiting for a new, suppressive HAART regimen to be feasible.

- - 1. Justification for 3TC or FTC Monotherapy

3TC or the closely related drug, FTC, is included in the dual nucleoside/nucleotide reverse transcriptase inhibitor (NRTI) backbones of all recommended first-line regimens (1). In non-adherent patients with suboptimal suppression on a 3TC- or FTC-containing regimen, the M184V mutation develops rapidly. No additional mutations occur with continuation of 3TC or FTC monotherapy. However, while this mutation confers high-level drug resistance to both 3TC and FTC, the presence of M184V reduces viral fitness, and has been associated with a reduction in viral load of approximately 0.5 log_10_ c/mL (13). M184V does not confer significant cross-resistance to other NRTIs and increases susceptibility of HIV to other ARV drugs [e.g., tenofovir disproxil fumarate (TDF), zidovudine (AZT), and stavudine (d4T)] (14). M184V-containing HIV variants typically fade from detection over a median of 20 weeks in the absence of drug pressure (15). Maintenance of the M184V mutation through on-going selective pressure from 3TC or FTC monotherapy, which occurs even in the setting of incomplete adherence to the drug, may help to keep viral fitness low. Therefore, there may be a benefit to utilizing 3TC or FTC in the setting of M184V resistance, to reduce the risk of selecting for additional drug resistance mutations and to limit viral replication during the bridge period between ending a failing HAART regimen and starting a new HAART regimen.

The potential success of this bridging strategy has been reported by Castagna et al. in a small, 48-week pilot study of adult patients with median CD4+ T cell counts of approximately 570 cells/mm^3^ who were failing HAART with the M184V mutation (29 patients/arm) and were randomized to either stop all ARV drugs (treatment interruption-TI) or to continue once-daily 3TC (300 mg) alone (16). At 48 weeks, 20 of 29 patients in the TI group (69%; 95% CI 51-83%) and 12 of 29 in the 3TC group (41%; 95% CI 26-59%) had discontinued the study due to immunological or clinical failure (*P* = 0.018). Immunologic failure was defined as a confirmed CD4+ T cell count <350 cells/mm^3^ or a new Centers for Disease Control and Prevention (CDC) class B or C event. Of note, the mean decline in CD4+ T cell count and percentage, viral rebound, and recovery of HIV replication capacity were all significantly less in the 3TC group (13;16). Additionally, patients in the 3TC group had lower viral load increases (+0.57 log _10_ HIV RNA) than patients in the TI group (+1.1 log_10_ HIV RNA). Only patients in the TI group (6/29, 20.7%) experienced Grade 3-4 clinical adverse events that were possibly related to HIV infection (*P* = 0.02). In patients who prematurely discontinued the study drug and subsequently re-initiated HAART, the 24-week virological and immunological responses after resumption in patients were similar to those of patients who had not been on 3TC.

Opravil et al presented limited data at the Interscience Conference on Antimicrobial Agents and Chemotherapy (ICAAC) 2009 and subsequently published on 26 clinically stable, significantly pre-treated adult (median age 43 years) patients (median CD4+ T cell count of 432 (IQR 378-540) cells/mm^3^) who were switched in an uncontrolled, one-armed study from a failing HAART regimen to 3TC monotherapy at 300 mg daily (17;18). Failure was defined as having a viral load greater than 400 copies/mL. The proportion of patients reaching the clinical endpoint of a ≥ 30% decline in CD4+ T cell count or a CD4+ T cell count <200 cells/mm^3^ at 24, 36, and 48 weeks was 36%, 57%, and 70%, respectively. None of the patients had any 3TC-related toxicity.

The investigators analyzed the subjects by the failing regimen at baseline (PI vs. RT-based HAART). The subjects in the two groups had similar baseline characteristics, including CD4+ T cell and reported that 81% of subjects on PI-based HAART vs. 40% of subjects on RT-based HAART reached the clinical endpoint. The decline in absolute CD4+ T cell was not statistically different between the two groups (overlapping confidence intervals), however, patients who were initially on PI-based HAART had higher increases in the viral replication capacity, presumably due to loss of pressure on viral strains with PI mutations. The authors conclude that 3TC monotherapy may be considered as a short-term strategy for “selected” patients failing RT-based HAART, but not PI-based regimens due to concern for increased viral fitness and greater likelihood of CD4+ T cell decline. The limitations of this study that were acknowledged by the authors included: the lack of a comparative group, the selective inclusion criteria, and the small sample size. The strengths, on the other hand, are a detailed virologic characterization of the isolates. Other limitations include not being a randomized study, no assessment of adherence, and no explanation why the patients were being considered for monotherapy (e.g., lack of available treatment options). It is unclear that the investigators had sufficient data to support the conclusion about RT vs. PI-based failing regimen and the impact of transitioning to 3TC monotherapy. The study population that was included was older (median age 43 years), pre-treated (median of 5 prior regimens), had advanced immunosuppression [30% with prior AIDS-defining illness (ADI)], and likely had extensive antiretroviral mutations all aspects that may be different than the population we propose to study. It is feasible that the younger, less immunologically suppressed population, failing primarily due to non-adherence may have a different outcome with 3TC monotherapy, particularly when compared to continuing failing HAART in the setting of non-adherence.

While these findings may suggest that there may not be a benefit to switching patients who are being successfully treated with HAART to 3TC monotherapy, there may be short-term clinical and/or immunological and/or virological benefit to 3TC monotherapy compared to continuation of a failing HAART regimen in select patients who are failing HAART primarily due to non-adherence. This is the premise of the proposed study.

- - 1. Importance of Adherence to Success of ARV regimens and Challenges of Incomplete Adherence

Greater than 95% adherence is important for durable virologic suppression and success of HAART (19;20). It is estimated that 20% of treatment-naïve patients and nearly 40% of treatment-experienced patients who initiate a new regimen fail that regimen within the first 1-2 years (21), primarily due to non-adherence. In the setting of suboptimal adherence, viremia occurs in the presence of continued drug pressure at suboptimal levels, resulting in development of resistant strains and viral escape.

*Challenges of Incomplete Adherence.*

Given the chronic nature of HIV disease, medication fatigue and decreased adherence to ARV regimens are pervasive problems. **For younger children, the caregiver is usually responsible for administering medications and when non-adherence is present, assessing the caregiver is essential to evaluating and addressing non-adherence.**  In older, school-aged, HIV-infected children, **caregivers** may begin to relinquish the responsibility of medication administration to the children and children may start to rebel against taking medication, resulting in incomplete adherence (22). For young HIV-infected individuals the root cause of non-adherence may be multifactorial, but include, not feeling sick, depression (19;23;24), non-disclosure of status, not knowing their own HIV status, being reminded of their HIV status by the medication, wanting to be normal, interference with daily life, simply forgetting, pill burden, side effects, palatability, and inability to swallow pills (25), in addition to chaotic lifestyles and psychosocial problems. These causes may be different depending on the patient’s mode of HIV acquisition, age, as well as who is responsible for administering the medication. It is critically important to identify the cause of the non-adherence as improvement depends on the initial source of the adherence problem. Self-reported adherence has been proven to correlate with virologic suppression (26). Greater self-efficacy and outcome expectancy have been shown to be significantly correlated with adherence (27). **Although the mechanisms of non-adherence might be different for younger children, specifically the role of the caregiver, the results of non-adherence (e.g., development of resistance, reduced treatment options) for all patients is the same. Research into treatment strategies to address treatment needs in the setting of perpetual non-adherence is important for HIV-infected patients of all ages.**

Several studies have highlighted markedly lower adherence rates in youth as compared to adults on similar ARV regimens (28;29). Higher risk of non-adherence and failure is an unfortunate consequence of periods of transition, as in the transition from early childhood to school age and during adolescence and young adulthood, or periods where more responsibility is being placed on the young individual for taking their medications as well as managing other aspects of their lives. In fact, in a recent study by Khan, et al., age >13 years was significantly associated with a low likelihood of achieving an undetectable viral load (26). Overall, children and adolescents have lower levels of adherence than adults on similar regimens (30;31). In PACTG 381, 41% of adolescents failed their first HAART regimen within the first 24 weeks of therapy (29). Khan et al. reported that 40% of children and adolescents were never or rarely adherent to their regimens based on 3 day recall (26).

Up to 80% of individuals who have failed prior regimens have some resistance with the resultant consequences for decreased response to subsequent regimens as well as transmission of resistant variants (32). Many patients who are adolescents or young adults, particularly those with behaviorally-acquired infection, have relatively few ARV drug resistance mutations, even though the majority develop the M184V mutation(2). However, these patients are at risk for accumulation of resistance mutations with continued non-adherence. Discontinuation of HAART is generally avoided in patients who meet treatment criteria, in light of data from the SMART study that highlights the increased morbidity risk secondary to HIV-related and non-related clinical events with treatment discontinuation; this increased morbidity risk may be related to rebound of inflammation after HAART discontinuation or longer time periods at lower CD4+ T cell counts (33). Alternative strategies that can lower HIV viral load and preserve CD4+ T cell counts are needed as an interim step in patients who need ARV treatment, but are unable to adhere to a HAART regimen. Bridging strategies would provide time for intensive efforts to improve adherence, which might improve the chance that these patients would succeed on a subsequent HAART regimen. If HIV variants with lower viral fitness could be maintained in such patients, use of a non-suppressive bridging regimen could still lower viral loads, decrease immune activation, and slow immunologic deterioration. In addition, the combination of a lower virologic burden and reduced viral fitness might reduce the risk of sexual transmission in this population that often continues to engage in high-risk behaviors (32).

*Simplification is likely to be advantageous to this population.*

Since the advent of HAART, regimens have become increasingly simplified. Potent once daily regimens (e.g., Atripla) are now available. Simplified regimens (e.g., once daily treatment regimens) have been associated with good response to therapy (34) when compared to more complex regimens. While this has been critically important to reducing pill burden, the low barrier to resistance of particularly the NNRTI-based regimen, in the setting of continued issues with non-adherence, increases the risk of developing resistance with non-adherence (2) and compromising future options. PI-based once daily regimens have a higher barrier to resistance, but trade off with higher pill burden and often more side effects. Nonetheless, the likelihood of adherence for the younger population certainly improves with lower pill burden, less frequent dosing, and reduced side effects. Monotherapy with 3TC/FTC may be advantageous as it provides all of those positive features and may preserve future treatment options by not incurring additional resistant mutations, while providing stabilization of CD4+ T cell, and time to address factors that may result in improved adherence. Because the patient’s current failing regimen is likely more complex (e.g., at least three medications taken once or twice daily), they may be taking suboptimal amounts of the regimen vs. if they were prescribed a simpler regimen that they may be more likely to adhere to.

Incomplete adherence with 3TC/FTC monotherapy may be sufficient to maintain the mutation without being harmful, while incomplete adherence with HAART is unlikely to have the same effect. Even with PI based therapy, the decreased viral fitness is not without the cost of resistance as well as greater likelihood of toxicity than 3TC/FTC.

Inherent to this discussion is that young individuals, over time, will become more ready to adhere to their medications with time, life experience, growth in both cognitive and social capacity, and that they will have improved self efficacy. Bridging may allow time for this to occur.

*Importance of measuring adherence.*

As adherence is essential to the success or failure of any regimen, assessing the impact of a bridging regimen on adherence is important to understanding the comparative effectiveness of both treatment strategies in the real world. It is possible that 3TC/FTC monotherapy will have a decreased pill burden and therefore those assigned to that regimen may have more ease and therefore better adherence. Without a good measure of adherence, the outcomes of such a study will not be fully explainable. Specifically, outcome is impacted by both efficacy of the regimen and adherence. The same apparent outcome may be attained for a regimen with high adherence and lower efficacy as for one with low adherence and high efficacy. Therefore, interpretation of the results of the study must be interpreted in the context of a reasonable measure of adherence. For the outcomes of the next optimal HAART regimen, the impact of a bridging strategy on adherence is also important. It should be noted that there may be heterogeneity of non-adherence in the eligible population since inclusion is based on provider perception, however, this heterogeneity should be balanced through randomization. We, thus, are measuring adherence as a part of a secondary aim to explore whether it is a predictor of outcome. In short, the assessment of adherence is not crucial to answering the primary study question, but is necessary to fully explain the study results.

There is, unfortunately, no gold standard for measuring adherence. Three day recall has been selected to measure adherence in this protocol as it has been shown to correlate with viral suppression (Van Dyke et al. Pediatrics 2002), can be employed in a standard way across all sites without the need for a psychologist or other trained personnel, is an existing adherence assessment and module that is standardly used in IMPAACT protocols, and is cost-effective. Given the limitations of more sophisticated measures, the team is very concerned about having sites spend significant amount of energy collecting adherence measures that may ultimately prove to be of marginal use. As the study is not distributing medications, creating variability in the known denominator of pills dispensed, there is no consistently available objective mechanism that can be applied to all of the sites to assess adherence; (e.g. pharmacy refills, pill count). Additionally, other measures such as serum drug levels or levels of drugs in hair are expensive and may not be cost-effective given the rationale for measuring adherence in this study.

*Rationale for not including an adherence intervention in the study.* Improving adherence is not a study objective. The goal of this study is to examine the effectiveness of two bridging strategies that can be used by clinicians for patients in need of HAART, while they work to improve adherence using the existing standard of care at their site. Further, there is no gold standard, consistently reliable adherence intervention. To reiterate, the goal of this protocol is to examine what can be done medically (from a treatment intervention perspective) while the clinical team continues to work on their optimized best practice adherence mechanisms.

- - 1. Acceptability and Feasibility of a RCT that Includes a 3TC or FTC Monotherapy Arm in Adolescents

An October 2009 survey of 75 domestic and international IMPAACT providers about management of non-adherent patients in need of treatment by DHHS guidelines suggests that providers continue to struggle with management of these patients, of 57 (76%) responded; 100% reported having these challenging patients in their practices. Sixty percent of respondents reported that they typically continue the failing regimen; only 5% reported using 3TC or FTC monotherapy as a bridging strategy for up to 12 months or until clinical or immunologic deterioration. The strategies that were considered most acceptable for managing these patients included: continuing the failing regimen (80%), discontinuing the failing regimen (60%), and use of 3TC or FTC monotherapy (40%). When asked, 50% of respondents stated that it would not be inappropriate to include a 3TC or FTC monotherapy arm in a clinical trial as a bridging strategy. Ninety-six percent indicated that they would be willing to enroll patients in a clinical trial of bridging therapy. This evidence from within the IMPAACT network suggests that while 3TC or FTC monotherapy is considered to be a potentially viable management strategy, the lack of evidence from a RCT may be inhibiting its use, establishing the need for such information and equipoise. Finally, a significant proportion of respondents felt that six months was an appropriate amount of time to use a bridging strategy.

There is prior evidence, through observational studies, supporting lower burden regimens, (e.g., dual-NRTI regimens) in children/adolescents/young adults (section 1.21). Current pediatric guidelines mention low burden/suboptimal regimens as potential strategies, but there are no well designed studies examining their efficacy, particularly in comparison to continuing a failing HAART regimen (20).

- - 1. Rationale for investigation of inflammation and immune activation

The primary goal of this study is to compare two antiretroviral treatments, both expected to be associated with viremia, used as a bridging strategy. Ongoing viremia in untreated HIV is associated with chronic immune activation and inflammation that are believed to contribute to HIV disease progression and possibly to other complications of HIV such as cardiovascular disease and dementia (35-37). Although less well studied, it would be expected that partially suppressive ART regimens will be associated with immune activation and inflammation. Determining whether the bridging strategies differ with respect to immune activation markers associated with disease progression or biomarkers of cardiovascular disease may be valuable in assessing which strategy will have the least harmful effects. Thus, a secondary objective of this study will be to investigate laboratory markers that are indicators of immune activation and inflammation. In addition the regimens will be compared with respect to the amount of microbial translocation, this being one of the factors that drives immune activation. The specific markers to be evaluated include:

1. Immune activation: flow cytometric assessment of CD38, HLA-DR, Ki67, CD95, apoptosis, and plasma levels of IL-6, TNF-alpha, IFN-alpha.
2. Cardiovascular risk: D-Dimer, hsCRP, MCP-1, sVCAM-1, sICAM-1, E-selectin, fibrinogen, and P-selectin, total, HDL, and LDL cholesterol, triglycerides
3. Microbial translocation: plasma LPS, 16srDNA, soluble CD14.

*Immune activation*

Chronic immune activation is a major contributor to CD4+ T cell decline (35;38). It will be valuable to determine whether there is a difference in immune activation between the arms. If one arm is associated with lower CD4+ T cell counts but not with higher viral load, finding a correlation with increased immune activation may provide insight into the mechanism underlying the difference. A major difference between the arms in this study is that many of the subjects who randomize to continue HAART will be receiving a protease inhibitor (PI). There are several studies that suggest that treatment with or without a PI could result in differences in activation and/or CD4+ T cell count. A study that examined the effect of discontinuation of protease inhibitors with continued NRTI treatment in a small number of adults (n=17) found no difference over 12 wk in levels of CD38/HLA-DR expressing CD8 T cells (39). However, in seven patients who had loss of PI mutations and reversion to wild type virus, increases in CD38 expression were observed, without increases in the magnitude of plasma viremia. Our study will be able to examine correlation of changes in viral genotype and/or replication capacity vs. markers of activation. Several studies have demonstrated that in addition to the direct anti-viral activity of PI’s, most of the drugs in the class also inhibit apoptosis demonstrated in vitro (40;41). The mechanism of the effect is not related to immune activation (40). Some authors have suggested this may be a mechanism leading to higher CD4+ T cell count recovery observed in studies comparing PI-based vs. NNRTI-based HAART (42). The current study offers an opportunity to compare frequencies of apoptotic T cells between the mono-therapy and the multi-drug therapy arms.

A major contributor to immune activation in HIV-1 infection is believed to be a compromised gut-associated lymphoid tissue (GALT) allowing microbial translocation (43). Circulating lipopolysaccharide (LPS) and 16SrDNA, markers of bacterial components entering the blood from the gut, are elevated in chronic HIV infection, are reduced with antiretroviral therapy, and remain elevated in patients with failure to recover CD4+ T cell counts after antiretroviral therapy (44). Elevated soluble CD14 (sCD14), evidence of the monocyte activation, is also observed and has been associated with a risk for HIV-associated dementia (45). It is not known whether different ART will be associated with differences in microbial translocation.

*Cardiovascular risk*

The selection of markers to be evaluated is based on a number of studies in adults demonstrating increased risk of adverse outcomes associated with the markers. Subset analysis of participants in the SMART (Strategies for Management of Anti-Retroviral Therapy) trial found IL-6, D-dimer, and hsCRP at baseline and at latest value were strongly associated with all-cause mortality (46). Elevated IL-6 and hsCRP was also found in the SMART study among participants receiving abacavir (ABC); ABC was also associated with a higher risk of cardiovascular disease events (47). A recent study using a large patient data registry found both HIV and elevated CRP/hsCRP independently associated with the risk of acute myocardial infarction (48). Endothelial dysfunction is a precursor to atherosclerosis and plasma markers of endothelial dysfunction have been shown to be associated with risk of development of coronary heart disease. A number of well established markers of increased atherosclerotic risk are soluble intercellular adhesion molecule 1 (sICAM-1), soluble vascular cell adhesion molecule 1 (sVCAM-1), monocyte chemoattractant protein-1 (MCP-1), E-selectin, and P-selectin. These markers have been found to be elevated in adults with HIV compared to controls (49;50). In addition, fibrinogen, P-selectin, sICAM-1, E-selectin were elevated in children with HIV compared to uninfected controls and were independent of abnormal lipid parameters (51). Finally, cholesterol and triglycerides are well established as risk factors for cardiovascular disease.

- - 1. Ethics of Studying 3TC/FTC in this population

Because the 3TC/FTC bridging regimen provides less-intensive ARV therapy than HAART-based alternatives, it may appear inappropriate to randomize participants to this study arm. However, proper subject selection should largely alleviate this ethical concern. This study is designed only to enroll subjects whose clinicians have determined that non-adherence represents an obstacle to fully effective therapy. For non-adherent patients, more HAART is not clearly better, due to the risk of developing new resistance mutations and losing future treatment options. This consideration, which is particularly salient in resource-limited settings where subsequent-line HAART options may be fewer, calls for research on appropriate treatment strategies for non-adherent patients. Since it is unlikely that any ART strategy can be fully effective in this population, research should aim to promote subject welfare while acknowledging that there are drawbacks to any approach.

Monotherapy bridging may balance the advantages and disadvantages of more- and less-intensive approaches: monotherapy reduces the risks of resistance mutations, relative to HAART, while possibly still providing protection against disease progression. Bridging strategies are particularly promising for HIV-infected adolescents, among whom short-term non-adherence is common but long-term improvements in adherence are possible. This population is likely to benefit from an approach to ART which manages disease progression in the short term and preserves long-term treatment options.

- - - 1. *Equipoise among treatment strategies*

To protect human subjects, IRBs/ECs and other reviewers must be satisfied that the study intervention compares favorably to the available alternatives. Analyzed under the clinical equipoise requirement, it should be uncertain whether the risk-benefit profile of the study intervention is superior to those of the control intervention and any other accepted standard of care. In this study, reviewers will note that monotherapy bridging diverges from the strategies endorsed by DHHS guidelines for the general population, as well as those preferred by some clinicians. However, deviating from recommended standards of care is generally acceptable as long as equipoise remains.

For the subject population which will be enrolled in this study, no optimal strategy exists. DHHS recommends switching HAART regimens or continuing failed HAART for patients with virologic failure. Among HAART-adherent patients experiencing virologic failure, monotherapy bridging would not stand in equipoise because such patients are well-positioned to benefit fully from either switching HAART regimens or continuing their regimen with close viral monitoring. Among non-adherent patients, however, these standard strategies carry lesser benefits and greater risks. As a result, they may not be superior to short-term monotherapy, especially for adolescents who are expected to remain non-adherent temporarily but may improve their adherence in the future.

Aside from the weaknesses of the existing strategies, several factors favor monotherapy bridging from a clinical standpoint. By maintaining the M184V mutation, monotherapy may slow disease progression despite being less active otherwise. Therefore, compared to continuing failed HAART in the context of non-adherence, monotherapy may be no less clinically effective. However, determinations of equipoise should account for all the clinical benefits which might cause a subject or provider to favor one intervention over another. Even if subjects in this study are slightly more likely to reach CD4+ T cell and virologic endpoints on monotherapy than on continued failed HAART, subjects may experience additional benefits that would favor monotherapy bridging over alternative strategies. They will likely retain more options for future therapy by avoiding additional new resistance mutations. Additionally, subjects may experience fewer side effects, which would promote health and possibly improve adherence. Based on these additional benefits, monotherapy bridging stands in clear equipoise with continuing failed HAART, the control intervention in this study.

The comparison to switching HAART regimens is distinct because switching HAART regimens would clearly represent the optimal regimen for some subjects with virologic failure on HAART. However, a subject’s enrollment on this study will reflect his or her clinician’s judgment that the use of a second- (or later) line HAART regimen would be inappropriate at this time, because of persistent difficulty with adherence. Following this judgment, a subjects is not worse off for having enrolled on the study—if changing regimens is not clinically appropriate, then the alternatives to bridging are continuing failed HAART or full treatment interruption, which appears suboptimal following the SMART trial. Additionally, the strategy of monotherapy bridging does not deny the importance of switching subjects off failed HAART—on the contrary, it suggests a realistic approach to obtaining maximal benefits from new regimens. This research serves an acute need for clinical care in some resource-limited settings, where secondary regimens must be used conservatively due to limited access.

- - - 1. *Safety and trial design*

Monotherapy bridging must be distinguished from treatment interruption strategies, which would suspend all ART for some period before initiating a new HAART regimen. Compared to continuing non-suppressive HAART or switching HAART regimens prior to improving adherence, treatment interruption would provide the relative advantage of avoiding further mutations which would compromise future treatment options. However, this benefit likely does not outweigh the risks associated with treatment interruption. Monotherapy bridging, on the other hand, offers the prospect of a significantly better risk-benefit profile.

Nonetheless, close clinical monitoring is essential in studies which remove some subjects from a generally well-established regimen. During the relatively short **randomized (28 week) bridging period with an additional observational 24 week** bridging period, subjects will be regularly evaluated for CD4+ T cell count, viral load and clinical events. Additionally, the decision to remain on the study intervention, at **28** and **40** weeks as well as throughout the initial **28** week bridging period, will be subject to the clinical judgment of the subject’s physician. Informed consent from subjects or their parents, as well as pediatric assent as appropriate, will ensure that the unique risk-benefit profile of monotherapy bridging is acceptable to those enrolled on the study.

- - - 1. *Additional human subject protections*

The bridging regimen on this study is tailored to the short-term needs of young subjects who have failed HAART due to non-adherence. For this population, improving adherence is an essential long-term goal: they must improve adherence before they can obtain the full benefits of a new HAART regimen. Accordingly, this study requires that each site will provide its most effective adherence counseling intervention to subjects in each study arm. These interventions represent each site’s best attempt to improve subjects ARV adherence during the study period. Although the study does not directly test these interventions, providing these interventions is critical to promoting the welfare of subjects on the trial.

Developing effective behavioral strategies to improve adherence among young subjects is an important long-term goal for the HIV treatment community. There remains significant room for improvement among these techniques, and this goal should not be ignored in favor of research on biomedical interventions. Thus, this study is intended to complement, not replace, behavioral research on methods to improve adherence among children, adolescents, and young adults. At the same time, clinicians’ experience suggests that there is likely no “magic bullet” available in this area. Non-adherence represents such an intractable problem in HIV treatment that clinicians cannot ignore the special therapeutic needs of non-adherent patients. This study addresses these special therapeutic needs.

Reviewers should note, however, that this study excludes subjects whose non-adherence results from structural barriers such as lack of resources (e.g., insurance, consistent transportation, money for co-pays). Whereas non-adherence due to developmental immaturity may be inevitable in many HIV-infected youth, non-adherence due to structural barriers raises unique ethical issues and cannot be addressed solely through clinical strategies. For these subjects, ARV strategies are just a small part of the appropriate societal response to disease- and adherence-related problems. This study focuses instead on those subjects who would benefit from interventions available solely within the clinical sphere.

- - - 1. *Regulatory Ethic Considerations*

Under 45 CFR 46 subpart D, IRBs should analyze pediatric research according to the levels of study-related risk and benefit involved. It might appear that subjects on this study are exposed to significant risk on either study regimen because neither strategy is optimal: on either arm, subjects may experience disease progression or drug resistance. However, determinations of study-related risk and benefit should measure the *difference* between study participation and standard clinical care outside the study. In this study, one trial arm (continuing failed HAART) represents the standard clinical response among subjects who are unready for new HAART. The other trial arm tests a regimen (3TC/FTC monotherapy bridging) which stands in equipoise because its overall risk-benefit profile is believed to compare favorably. Therefore, the choice of study regimens is not expected to create risks or benefits differing significantly from standard clinical care, in which no optimal strategy exists. Moreover, study testing does not involve any additional procedures which are incommensurate with standard clinical testing. Therefore, the risks and benefits of this study overall are not expected to differ significantly from standard clinical care. Finally, children and adolescents are an appropriate target population in this study because they are particularly well positioned to benefit from improved bridging approaches.

**1.27 Summary of rationale**

**Many HIV-infected pediatric and adolescent patients meet the criteria to receive and are in need of antiretroviral therapy.  However, the need to maintain a high level of adherence in order to avoid viremia, development of resistance, immune deterioration, as well as secondary transmission is often unattainable for a substantial proportion of patients.  With a significant number of these patients having adherence challenges, despite intervention, there are no optimal alternative treatment strategies (e.g., continued HAART in the setting of documented nonadherence and failure or discontinue HAART).  Both of these currently employed strategies can prove to be deleterious.  Bridging treatment strategies, such as 3TC/FTC monotherapy, may afford these patients and their providers time to work on adherence and other barriers to successful treatment while maintaining a stable immune status.  The use of 3TC/FTC monotherapy as a strategy does not have resistance consequences, even when patients continue to exhibit poor adherence as is often the case in the population of focus, and may still support the immune system while working on adherence.**

1. STUDY OBJECTIVES
   1. Objectives for Step 1- Randomized Study (enrollment to **Week 28**)
      1. Primary Objective
         1. To compare immunologic deterioration during a **28** week “bridging” treatment strategy of 3TC or FTC monotherapy vs. continuing HAART in HIV-infected children, adolescents, and young adults with virologic failure who are likely to be non-adherent to an optimized HAART regimen (due to problems related to adherence, tolerability, or toxicity). Indicators of immunologic deterioration will be a ≥ 30% decline in absolute CD4+ T cell count or development of CDC class C events.
      2. Secondary Objectives
         1. To compare the change in genotypic HIV drug resistance from baseline to **week 28** in the two study arms.
         2. To compare the slope of CD4+ T cell count and percent over **28** weeks between the two study arms.
         3. To examine factors (e.g., demographic, immunologic, and virologic) associated with immunologic deterioration.
         4. To compare the change in HIV viral load and CD4+ T cell count from baseline to **week 28** in the two study arms.
         5. To compare changes in immune activation and cardiovascular risk factors (e.g., CD8+/CD38+/HLA-DR+T lymphocytes, hsCRP, LDL, and HDL cholesterol) from baseline to **week 28** in the two study arms.
         6. To compare the rates of adverse events in the two study arms.
         7. To compare adverse clinical outcomes (e.g., bacterial infections, hospitalizations, HIV-related and non-HIV-related morbidity) in the two study arms.
         8. To compare adherence for subjects in the two study arms, as measured by 3-day recall.
         9. To compare the change in phenotypic HIV drug resistance and HIV replication capacity from baseline to **week 28** in the two study arms in a subset of participants.
   2. Objectives for Step 2 (Weeks **28** to **52**)
      1. Secondary Objectives
         1. To compare the proportion of subjects in the two originally assigned treatment arms who do not experience immunologic failure at **40** and **52** weeks and to identify factors (e.g., demographic, immunologic, and virologic) predictive of experiencing immunologic failure.
         2. To compare CD4+ T cell count changes from **28** weeks to **40** and **52** weeks in subjects, in the two originally assigned treatment arms.
         3. To compare the mean change in genotypic HIV drug resistance in subjects in the two originally assigned treatment arms.
         4. To compare adherence to the study regimens at **40** and **52** weeks among subjects in the two originally assigned treatment arms.
         5. To compare changes in immune activation and cardiovascular risk factors (e.g., CD8+/CD38+/HLA-DR+T lymphocytes, hsCRP, LDL, and HDL cholesterol) from baseline to **52** weeks in the two originally assigned treatment arms.
         6. To compare the rate**s** of virologic suppression and CD4+ T cell count recovery among subjects in the two originally assigned treatment arms 24 weeks after **starting Step 2**.
         7. To compare adherence to a new HAART regimen among subjects in the two originally assigned treatment arms.
2. STUDY DESIGN

IMPAACT P1094 is a multi-center (domestic and international), open-label, Phase IV randomized, controlled, comparative trial to evaluate continuation of non-suppressive HAART (Arm A) vs. 3TC or FTC monotherapy (Arm B) for **28** weeks (in both arms), in HIV-1 infected school-aged children, adolescents, and young adults ages ≥8 to <25 years of age who have documentation of the M184V mutation and are failing their current HAART regimen **and are persistently non-adherent**. Failure of their current regimen is defined as a confirmed* (*two viral loads from two different draws) viral load ≥400 copies/mL at least **2** months after initiating the current regimen and at screening. The study will span **52** weeks, the initial **28**-week randomized study period will be followed by a 24-week follow-up period. The target accrual is 344 subjects (172 subjects/arm) with the goal of having **310** evaluable subjects (to account for an estimated 10% attrition). Enrollment will be stratified by CD4+ T cell count ≥/<400 cells/mm^3^ to allow subsequent analysis of the impact of baseline CD4+ T cell count on response to the bridging strategy.

Figure 1. P1094 Study Design Diagram

Step 2: > Week **28**

Week 0: Subjects whose providers are not planning to stop their HAART in the setting of virologic failure are randomized to 3TC or FTC Bridging vs. Continuing Non-suppressive HAART. Subjects commit to at least 6 months on the randomized study regimen and only switch if they reach clinical/primary endpoints as defined in the protocol.

Week **28**: completion of randomized study. At this time, the primary outcomes will be compared between the two study arms. **Subjects should** continue to step 2.

Step 2 (Week >**28 to 52**): Subjects may: (a) continue randomized treatment, (b) begin a new HAART regimen , if the clinical team including the subject, and or caregiver (as appropriate) determine that it is clinically necessary or adherence is likely, or (c) discontinue study treatment, but remain on study follow-up. This decision will be made by the subject and their clinical team and not the study team.

Note: Beyond week **52**, follow-up of outcomes on new HAART regimen will be done through data collected in P1074 (highly encouraged co-enrollment).

*The decision will be made by the subject and their clinical team and not the study team.

Begin new

HAART

regimen*

Continue randomized treatment*

Discontinue therapy, but remain on

follow up*

END OF STUDY (assessment of objective for Step 2)

HIV-Infected Children, Adolescents, and Young Adults Failing HAART with the M184V Mutation and Persistently Non-Adherent

Arm A: Bridging Therapy

Continue Non-suppressive HAART (x**28** weeks) 172 subjects

Primary endpoint. Subsequent ART as determined by clinical care

Primary endpoint. Subsequent ART as determined by clinical care

Continue randomized treatment*

Arm B: Bridging Therapy

3TC or FTC Monotherapy

(x**28** weeks) 172 subjects

344 Subjects Randomized

Week **28**

Step 1: Week 0

Begin new

HAART regimen*

Week **52**

Discontinue therapy, but remain on

follow up*

It is expected that subjects that are eligible for this protocol are those who have continued to be non-adherent despite counseling and interventions by the sites.  Providers are expected to provide justification for their judgment of non-adherence and their unsuitability for a new regimen, based on, but not limited to at least one of the following: subject/caregiver admission of incomplete adherence, persistent viremia despite reportedly taking the drug regimen and without other plausible explanation (e.g., drug interactions), pill counts inconsistent with reported pill intake, pharmacy prescription refill history consistent with incomplete adherence, drug levels (if measured) inconsistent with report of medication intake, or agreement of incomplete adherence by two separate providers (one who is not the subject’s primary provider).  The mechanism may be site-specific and will be collected at study entry.  It is also expected that sites continue to employ their standard best-practice interventions to enhance adherence for all subjects on study.

Subjects failing non-NNRTI-based HAART will be eligible. For subjects assigned to Arm A, the protocol requires that the subject continue their current HAART regimen, which is being prescribed and monitored by the subject’s provider. The exception is when subjects are on regimens that include a NNRTI. Current HAART that includes a NNRTI is allowed as long as the regimen also includes at least 3 drugs from at least 2 other classes. In such cases, the NNRTI will be discontinued and the remainder of the regimen will be continued as the on-study HAART regimen. The exception to the 2 class requirement would be acceptable 3-NRTI regimens (e.g., AZT/3TC/ABC).

NNRTI drugs are not being allowed as a component of the continuing failing HAART arm due to the concern for accumulation of additional NNRTI resistance mutations that would compromise second generation NNRTIs (e.g., etravirine).

All subjects assigned to the monotherapy arm (Arm B) will receive either 3TC or FTC (the choice of 3TC or FTC will be left to the provider). This decision will be likely determined by the location (domestic vs. international), the ease of acquiring each drug, and the ability to take pills or not. The total daily dosing for 3TC and FTC should be based on the recommendations by the U.S. Food and Drug Administration (FDA).

The **52** week study consists of two sequential periods:

- Step 1, the randomized study period of **28** weeks during which subjects are randomized to one of two study-assigned treatment arms, and
- Step 2, the 24-week follow-up period following the initial **28**-week period.

Subjects continue the treatment during Step 1 unless they meet the study endpoints. During the subsequent 24 weeks (Step 2), subjects who have not yet met study endpoints and are still determined by the clinical site team including subject and/or parent/guardian (as appropriate) to not be likely adherent to a new/optimal HAART regimen over the ensuing 24 weeks, may be continued on the study assigned therapy for an additional 24 weeks. During this follow-up period, subjects will continue on Step 2, and may: (a) continue randomized treatment, (b) begin a new HAART regimen, if the clinical team including the subject, and or caregiver (as appropriate) determine that it is clinically necessary or adherence is likely, or (c) discontinue study treatment, but remain on study follow-up. This decision will be made by the subject and their clinical team and not the study team. For all subjects who are not continuing study assigned treatment, the subsequent regimen and reasons for switching should be provided to the P1094 protocol team.

Co-enrollment in P1074 will be offered to each subject (at domestic sites) in P1094 to passively collect longitudinal outcomes, principally, response to subsequent HAART regimens. See section 4.6.

- 1. Screening Log

Subjects who are eligible for enrollment to P1094 will be identified through the site’s standard screening procedures. In order to assess the relevance of the tested strategies to the population of eligible subjects, the process of enrollment to P1094 requires that site investigators provide aggregate information on all subjects who met study criteria but were not enrolled on study. Analogous to what is done in P1025 (another IMPAACT study), a log of all subjects who are eligible for P1094 (whether or not they enroll or participate in the study) should be maintained. The screening log will collect limited grouped data on eligible subjects. The specific information to be collected includes CD4 T+ cell count range (e.g., **100-150; 150-200**; 200-250; 250-300; 300-350), viral load category (400-1,000; 1,000- <10,000; 10,000-100,000 copies/mL), treatment regimen and reason failing, age category (e.g., 8-12; 13-19; 20-24 years), gender, race/ethnicity, and reason for not enrolling (e.g., subject refusal and why*; provider preference, subject unavailable), if applicable. These data are being collected to determine if there are differences between subjects that were enrolled and all potential/eligible subjects. The site will be required to submit grouped summary data for each calendar quarter (i.e., the total number of subjects in each category of each characteristic collected in the log) to the IMPAACT Data Management Center.

***Subject refusal examples include time, distance, concerns about confidentiality, mistrust in research, participation in a conflicting protocol, concerns about specimen collection.

*Need for waiver of consent.* The team believes that P1094, similar to P1025, does not require a waiver of consent for screening and maintaining the screening log, but the final determination rests with the local IRB at each site.

Refer to Appendix I, Schedule of Evaluations, for specific study requirements.

1. SELECTION AND ENROLLMENT OF SUBJECTS
   1. Step 1 - Inclusion Criteria
   2. Age ≥ 8 to < 25 years of age, at study entry.
   3. Documentation of HIV-1 infection defined as positive results from two samples collected at different time points. The same method may be used at both time points. All samples tested must be whole blood, serum or plasma. Results documented in the clinical record from past testing may be used to satisfy the criteria for documentation of HIV-1 infection.

Acceptable tests when subjects are diagnosed at ≤ 18 months of age

The first test may be any of the following:

- One HIV DNA PCR
- One HIV RNA PCR (quantitative >5,000 copies/mL or qualitative)
- One HIV culture (prior to August 2009)
- One total HIV nucleic acid

If the first test is positive, a second sample must be collected and tested using any of the tests listed above (except for qualitative RNA assays) in a laboratory participating in an appropriate external quality assurance program and either CAP/CLIA approved (for US laboratories) or DAIDS-approved (for international laboratories).

Acceptable tests when subjects are diagnosed at > 18 months of age

The first test may be any of the following:

- Two rapid antibody tests from different manufacturers or based on different principles and epitopes
- One rapid antibody test AND one [enzyme immunoassay (EIA) OR Western blot (WB) OR immunofluorescence OR chemiluminescence]
- One EIA AND one [WB OR immunofluorescence OR chemiluminescence]
- One HIV DNA PCR
- One HIV RNA PCR (quantitative >5,000 copies/mL or qualitative)
- One HIV culture (prior to August 2009)
- One total HIV nucleic acid

If the first test is positive, a second sample must be collected and tested using any of the tests listed above (except for qualitative RNA assays) in a laboratory participating in an appropriate external quality assurance program and either CAP/CLIA approved (for US laboratories) or DAIDS-approved (for international laboratories).

- 1. **Treatment experienced patients must demonstrate failure**^†^ **on the current HAART regimen for ≥ 2 months.  These patients must have been on ARVs for at least a total of 6 months prior to entry.  Thus, if the failing regimen is the first ARV regimen, then the patient must have been on that initial regimen for a minimum of 6 months total.**

Note: The current HAART regimen must include at least three non-NNRTI drugs from at least 2 classes (i.e., PI, NRTI, integrase inhibitor, CCR5 antagonist, fusion inhibitor), OR at least 3 NRTIs that constitute a HAART regimen (e.g., ABC/ZDV/3TC).

^†^Virologic failure is defined as a confirmed* (*two viral loads from two different draws) viral load **≥**400 copies/mL at least **2** months after initiating the current regimen **and at screening**. The viral load results must be within 6 months of study entry.

- 1. CD4+ T cell count ≥**100** cells/mm^3^ (confirmed on at least two occasions within 6 months of study entry, including the screening value).
  2. Documentation of the M184V mutation on genotypic testing at any time prior to study entry.
  3. In the best judgment* of the clinical site team, concerns about the subject’s ability to adhere makes it unsuitable to initiate a new optimal HAART regimen for at least 6 months.

*Determination may be made (and documented) by mechanisms that may include but are not limited to the following: patient/caregiver admission of incomplete adherence, persistent viremia despite reportedly taking the drug regimen and without other plausible explanation (e.g., drug interactions), pill counts inconsistent with reported pill intake, pharmacy prescription refill history consistent with incomplete adherence, drug levels (if measured) inconsistent with report of medication intake, or agreement of incomplete adherence by two separate providers (one who is not the patient’s primary provider). The mechanism may be site-specific and will be collected at study entry.

- 1. Subject has not become adherent despite site’s adherence interventions*

*Adherence interventions that may have been employed include, but are not limited to: counseling, frequent clinic visits, reminders (watches, beepers, cell phones, telephone calls, other), directly observed therapy (e.g., hospitalization, outreach worker), g-tube placement, home visits, individual or group therapy, medication changes (e.g., regimen simplification), rewards/incentives (e.g., money, cell phone minutes).

- 1. Female subjects of reproductive potential (having reached menses, and not having reached menopause or not having undergone hysterectomy, bilateral oophorectomy, or tubal ligation) who engage in sexual activity that could lead to pregnancy must agree to avoid pregnancy during the entire **52** week trial and to consistently and appropriately use at least two of the following contraception methods: condoms, diaphragm or cervical cap with spermicide, IUD, hormonal-based contraception. A list of acceptable methods can be found at the FDA Birth Control Guide (<http://www.fda.gov/womens>).

Note: “Female subjects of reproductive potential” is defined as females who have reached menarche and who have not been post-menopausal for at least 24 consecutive months (e.g., who have had menses within the preceding 24 months), and have not undergone a sterilization procedure (hysterectomy, bilateral oophorectomy or salpingotomy). If the female subject is not of reproductive potential, she is eligible without requiring contraception.

- 1. Parent/legal guardian or subject able and willing to provide signed informed consent when applicable.
  2. Step 1 - Exclusion Criteria
  3. Positive hepatitis B surface antigen **or known active hepatitis B infection**.
  4. Pregnant or breastfeeding.
  5. Active malignancy within the past 2 years.
  6. Current immunosuppressive therapy, including the equivalent of > 1 mg/kg/per day or >20 mg total daily dose of prednisone in the 2 weeks preceding screening. Subjects for whom long-term systemic corticosteroid therapy (> 2 weeks) is anticipated are excluded. [Note: non-steroidal anti-inflammatory agents and inhaled, nasal, and topical corticosteroids are not excluded as immunosuppressive therapy.]
  7. Prior immunization with an HIV-specific vaccine.
  8. ≥ 1 CDC class C event (see Appendix II and III) within the past 12 months.
  9. Renal disease (as defined by estimated creatinine clearance <50 mL/min/1.73m^2^ confirmed on two occasions within 3 months of screening).
  10. Active opportunistic infections, including active tuberculosis (TB).
  11. Current treatment for active systemic TB. If recent, infection must have completed treatment course. INH treatment for latent TB is allowed.
  12. Viral load >250,000 copies/mL at screening.
  13. Known ≥Grade 3 of any of the following laboratory toxicities within 30 days prior to study entry: neutrophil count, hemoglobin, platelets, AST, ALT, lipase, serum creatinine. Note: Subjects can be re-screened and enrolled if repeat value is < Grade 3 without signs or symptoms of related organ dysfunction.
  14. Known ≥Grade 4 laboratory toxicities within 30 days prior to study entry, except with approval of the study team.
  15. For subjects who are not currently taking 3TC or FTC: Documented prior intolerance or adverse effect reasonably attributed to 3TC or FTC that resulted in permanent discontinuation.
  16. Problems with non-adherence attributed to modifiable structural barriers, such as lack of resources (e.g., insurance, transportation)*.

*Sites need to refer subjects that have failed screening due to modifiable barriers to appropriate services to address their recognized barriers.

- 1. Step 2 - Inclusion Criteria
     1. Met requirements for completion of Step 1.
     2. Subject/guardian agree to continue participation in Step 2
     3. ViroSeq assay results have been received by site and reviewed by investigator.
  2. Concomitant Medication Guidelines
     1. Precautionary Medications

The following medications should be avoided, if possible, and alternative treatments sought. Please contact the protocol team at [impaact.teamp1094@fstrf.org](mailto:impaact.teamp1094@fstrf.org) if treatment with any of these medications is necessary for clinical care.

- - - 1. Concomitant use of complementary/alternative medicines is strongly discouraged while subjects are on study.
      2. Inhaled, intranasal and topical corticosteroids in subjects taking boosted PIs. Note: Inhaled and intranasal fluticasone are disallowed in subjects taking boosted PIs.
      3. Medications for which package insert of co-administered ARV drug(s) (other than 3TC and FTC) cautions against use with that ARV drug in the subject’s HAART regimen. Sites must refer to the most recent study drug’s package insert to access additional current information on prohibited and precautionary medications. To avoid drug interaction and adverse events, the manufacturer’s package inserts of the ARV and concomitant agent should always be referred to whenever a concomitant medication is initiated or dose changed.
    1. Disallowed Medications
       1. Immunomodulatory therapy, including IL-2, any interferon product, GM-CSF, or thalidomide. [Note: G-CSF and erythropoietin are allowed.]
       2. Inhaled and intranasal fluticasone in subjects taking ritonavir-boosted PIs.
       3. 3TC in combination with FTC.
       4. d4T in combination with ZDV.
       5. Medications contraindicated for co-administration with one of the ARV drugs in the subject’s HAART regimen, according to the package insert for the ARV drug(s). Sites must refer to the most recent study drug’s package insert to access additional current information on prohibited and precautionary medications. To avoid drug interaction and adverse events, the manufacturer’s package inserts of the ARV and concomitant agent should always be referred to whenever a concomitant medication is initiated or dose changed.
  1. Enrollment Procedures

Prior to implementation of this protocol, and any subsequent full version amendments, each site must have the protocol document and the consent form(s) approved, as appropriate, by their local Institutional Review Board (IRB)/Ethics Committee (EC) and any other applicable regulatory entity (RE). A Site Implementation Plan (SIP) is required from each site participating in the study. The plan must be submitted to the Protocol Team for review and approval before protocol registration can occur.

Upon receiving final approval, sites will submit all required protocol registration documents to the DAIDS Protocol Registration Office (DAIDS PRO) at the Regulatory Support Center (RSC). The DAIDS PRO will review the submitted protocol registration packet to ensure that all of the required documents have been received.

Site-specific informed consent forms (ICFs) *WILL* be reviewed and approved by the DAIDS PRO and sites will receive an Initial Registration Notification from the DAIDS PRO that indicates successful completion of the protocol registration process. A copy of the Initial Registration Notification should be retained in the site's regulatory files.

Upon receiving final IRB/EC and any other applicable RE approval(s) for an amendment, sites should implement the amendment immediately. Sites are required to submit an amendment registration packet to the DAIDS PRO at the RSC. The DAIDS PRO will review the submitted protocol registration packet to ensure that all the required documents have been received. Site-specific ICF(s) *WILL NOT* be reviewed and approved by the DAIDS PRO and sites will receive an Amendment Registration Notification when the DAIDS PRO receives a complete registration packet. A copy of the Amendment Registration Notification should be retained in the site's regulatory files.

For additional information on the protocol registration process and specific documents required for initial and amendment registrations, refer to the current version of the DAIDS Protocol Registration Manual.

- - 1. Screening Log

The process of enrollment to P1094 requires the site’s maintenance of a log of all subjects who are eligible for P1094 (whether or not they enroll or participate in the study). See section 3.1. The site will be required to submit grouped summary data for each calendar quarter (i.e., the total number of subjects in each category of each characteristic collected in the log) to the IMPAACT Data Management Center to be keyed.

- 1. Co-enrollment Procedures

Please contact the protocol team at [impact.teamp1094@fstrf.org](mailto:impact.teamp1094@fstrf.org) with questions related to co-enrollment.

- - - 1. Subjects enrolled in treatment studies such as P1066, “*A Phase I/II, Multicenter, Open-Label, Noncomparative Study of the International Maternal, Pediatric, Adolescent AIDS Clinical Trials (IMPAACT) Group to Evaluate the Safety, Tolerability, Pharmacokinetics, and Antiretroviral Activity of Raltegravir (Isentress™, MK-0518) in HIV-1 Infected Children and Adolescents*” and are NOT on study provided treatment are permitted to co-enroll in P1094.
      2. Subjects enrolled in P1094 who complete study **week 28** may enroll in treatment studies (such as P1066) at that time. In this case, both protocol teams must be notified.
      3. Co-enrollment in P1074, “*A Prospective Surveillance Study of Long-Term Outcomes in HIV-Infected Infants, Children and Adolescents*” is encouraged and should be offered to each eligible subject in P1094.
      4. All other co-enrollments in protocols require the permission of the protocol chairs of the main protocol and the co-enrollment protocols.

1. STUDY TREATMENT
   1. Drug Regimens, Administration and Duration
      1. Step 1- Randomized Study (Enrollment to **Week 28**)

Study subjects will be randomized at a 1:1 ratio to Arm A (current non-NNRTI HAART) OR Arm B (lamivudine (3TC) or emtricitabine (FTC) monotherapy) for **28** weeks.

- - - 1. Arm A (non-NNRTI HAART)

Study subjects randomized to this treatment arm must continue to receive the same HAART regimen that they were receiving before entry (Exception: Discontinue NNRTI in selected cases- see below and section 4.13). The study does not assign or direct which ARV agents are used.

There is no preferred or study-supplied HAART regimen for this protocol. The study, however, excludes some subjects who are on NNRTI-based HAART regimens (see section 4.13). The study does allow inclusion of subjects whose pre-entry regimens include an NNRTI but also include at least 3 other drugs (from 2 other drug classes or 3 NRTIs, such as ABC/ZDV/3TC, recognized as a regimen). In these cases, the study requires that the subject discontinue the NNRTI at entry and continue all other ARV drugs in the pre-entry regimen.

- - - 1. Arm B (Monotherapy)

Study subjects randomized to this treatment arm will receive either lamivudine (3TC) OR emtricitabine (FTC) (as selected by prescriber). Subjects in the 3TC/FTC monotherapy whose antiretroviral regimen includes NNRTI(s) should discontinue their NNRTIs a week prior to discontinuing other ARVs. This is essential to minimizing the risk of developing NNRTI mutations.

The total daily dose of 3TC or FTC should generally be within the FDA-approved total daily dose of that drug.  Use of a total daily dose outside of the FDA-approved range may be permitted but only with approval from the P1094 team.

Note: Specific dosing will be determined at the discretion of the authorized prescriber.

- - 1. Step 2 - (Weeks **28** to **52**)

After completion of the randomization study, study subjects will transition to Step 2 and either (a) continue randomized treatment, (b) begin new HAART regimen, if the clinical team including the subject, and or caregiver (as appropriate) determine that it is clinically necessary or adherence is likely, or (c) discontinue study treatment, but remain on study follow-up. This decision will be made by the subject and their clinical team and not the study team.

The protocol does not define what constitutes optimal new HAART for those subjects who are subsequently switched. The new regimen is defined by the provider in conjunction with the subjects. Likewise, secondary and subsequent regimens are not defined by this protocol, and should be determined at the discretion of the site clinicians.

Subjects must be registered to Step 2 through the Statistical and Data Management Center (SDAC) randomization system. Entry into Step 2 **can** occur **any time after entry up to week 34**.

- 1. Drug Supply, Distribution and Pharmacy

No ARV therapy will be provided through this study. All ARV medication will be determined and prescribed by the authorized prescriber and supplied locally. Note: Use of generic drugs on the FDA-approved/tentatively approved list is acceptable. For international sites, generic ARV agents that are deemed appropriate by the sites are acceptable.

1. SUBJECT MANAGEMENT
   1. Toxicity Management

The DAIDS Table for Grading the Severity of Adult and Pediatric Adverse Events, (DAIDS AE Grading Table), Version 1.0, December 2004, Clarification August 2009, must be used and is available on the RSC website at (<http://rsc.tech-res.com/safetyandpharmacovigilance/>).

Management of adverse experiences will be according to the best clinical practice and the judgment of the site investigator. Alternate explanations for clinical and laboratory abnormalities must be sought. Laboratory normals will be the institutional values. However, if a site does not have an age-specific normal range/value for a particular laboratory value, the site should use the latest edition of the Harriet Lane Handbook for normal ranges/values and document this for monitoring purposes. Abnormal clinical and laboratory findings should be followed until resolution to <Grade 2.

For all grade 3 toxicities, the site provider should stop, continue or replace the treatment regimen as indicated according to the best clinical judgment. The team should be notified of any grade 3 toxicity and the antiretroviral plan adopted by the investigator.

For all grade 4 toxicities, the site provider should replace any component of the treatment regimen to which the toxicity may be reasonably related. Ideally, the replacement should be another antiretroviral in the same class. If it is not possible to replace a medication, if the likelihood is extremely low that the medication is the cause of the toxicity, or if the provider feels it is not in the best interest of the subject, the provider may continue current medications or discontinue antiretroviral medications as best benefits the subject. The provider should notify the team of all such decisions.

- 1. Subject Management

Step 1

Subjects will be randomized at a 1:1 ratio to continue current HAART (Arm A) OR to receive FTC or 3TC monotherapy (Arm B).

Subjects in Arm A will immediately discontinue the NNRTI component of their HAART, if applicable, but will continue all other ARVs. They must have a regimen that includes at least three non-NNRTI drugs from at least 2 classes (i.e., PI, NRTI, integrase inhibitor, CCR5 antagonist, fusion inhibitor), OR at least 3 NRTIs that constitute a HAART regimen (e.g., ABC/ZDV/3TC).

Subjects in Arm B whose antiretroviral regimen includes NNRTI(s) should discontinue their NNRTIs a week prior to discontinuing other ARVs and begin 3TC OR FTC monotherapy as per section 3.0. This is essential to minimizing the risk of developing NNRTI mutations.

Subjects will continue on their randomized therapy through at least **week 28** unless they meet a stopping criterion or the subject (guardian) or provider feels it is not in the best interest of the subject.

Upon completion of Step 1 (**any time after entry up to week 34**), the subject will move to Step 2.

Step 2

Upon entry into Step 2, subjects on each arm will have 3 options, as determined by the clinical site team including subject and/or parent/guardian (as appropriate):

1. Continue randomized treatment
2. Begin new HAART regimen if the clinical team including the subject, and or caregiver (as appropriate) determine that it is clinically necessary or adherence is likely
3. Discontinue study treatment but remain on study follow-up

Subjects will continue on Step 2 on whichever arm and option they have chosen for 24 weeks unless they meet a stopping criterion or the subject (guardian) or provider feels it is not in the best interest of the subject.

It expected that sites continue to employ their standard best-practice interventions to enhance adherence for all subjects on study.

- 1. Criteria for Stopping Randomized Treatment

1. New CDC class C event (See Appendix II and III)
2. CD4+ T cell count decrease of **≥** 30 % from **step** entry (during either **step**) **and confirmed by repeat test within one to six weeks**
3. Viral load increase from **step** entry to > 500,000 copies/mL maintained at 2 successive visits at least 1 day apart
4. Development of drug resistance mutations (not present on prior genotypes) or changes in drug susceptibility that change the resistance pattern (predicted for genotypic testing, measured for phenotypic testing) from susceptible to resistant for one or more drugs in the regimen or in likely future regimens
5. Subject requires treatment with medications that are disallowed in Section 4.421 and Section 4.422.
6. Pregnant women may remain on study, but in the observation/follow-up phase
   1. must be prescribed an ARV regimen that does not contain efavirenz in the first trimester of pregnancy
   2. all subjects on FTC/3TC monotherapy must follow the local standard of care for the duration of pregnancy

Note: In the event that a subject becomes pregnant, sites are encouraged to register the subject’s pregnancy in the Antiretroviral Pregnancy Registry (<http://www.apregistry.com/reg.htm> (In US, Canada: 1-800-258-4263, international: 1-910-679-1598.)

- 1. Criteria for Earlier (before **week 28**) Completion of Step 1
- The subject (guardian) or provider believe that continuing on the treatment regimen is not in the best interest of the subject
- Drug toxicity as defined in Section 6.1
  1. Criteria for Study Discontinuation
- The subject or legal guardian refuses further treatment and/or follow-up evaluations.
- The investigator determines that further participation would be detrimental to the subject’s health or well-being.
- The subject fails to comply with the study requirements so as to cause harm to him/herself or seriously interfere with the validity of the study results.

1. EXPEDITED ADVERSE EVENT REPORTING
   1. Adverse Event Reporting to DAIDS

Requirements, definitions and methods for expedited reporting of Adverse Events (AEs) are outlined in Version 2.0 of the DAIDS EAE Manual, which is available on the RSC website at <http://rsc.tech-res.com/safetyandpharmacovigilance/>.

The DAERS internet-based reporting system must be used for expedited AE reporting to DAIDS. In the event of system outages or technical difficulties, expedited AEs may be submitted via the DAIDS EAE Form. For questions about DAERS, please contact DAIDS-ES at [DAIDS-ESSupport@niaid.nih.gov](mailto:DAIDS-ESSupport@niaid.nih.gov) or from within the DAERS application itself.

Sites where DAERS has not been implemented will submit expedited AEs by documenting the information on the current DAIDS EAE Form. This form is available on the RSC website: <http://rsc.tech-res.com/safetyandpharmacovigilance/>. For questions about EAE reporting, please contact the RSC ([DAIDSRSCSafetyOffice@tech-res.com](mailto:DAIDSRSCSafetyOffice@tech-res.com)).

- 1. Reporting Requirements for this Study
- The SUSAR Reporting Category, as defined in Version 2.0 of the DAIDS EAE Manual, will be used for this study.
- The study agents for which expedited reporting are required are: zidovudine, abacavir, lamivudine, emtricitabine, didanosine, ritonavir, lopinavir, atazanavir, darunavir, tipranavir, fosamprenavir, indinavir, nelfinavir, tenofovir, saquinavir, raltegravir, maraviroc, etravarine, efavirenz, nevirapine, **and rilpivirine**.
  1. Grading Severity of Events

The most current Division of AIDS Table for Grading the Severity of Adult and Pediatric Adverse Events (DAIDS AE Grading Table) is used and is available on the RSC website at <http://rsc.tech-res.com/safetyandpharmacovigilance/>.]

*For reporting of all adverse experiences the Investigator will determine the causality and relationship to study drug. However, in regards to subject safety and PK evaluations which will support the selection of a dose for a given cohort, the protocol team will also have input as to the causality and drug relation of specific adverse experiences.

- 1. Expedited AE Reporting Period
- The expedited AE reporting period for this study is the entire study duration for an individual subject (from study enrollment until study completion or discontinuation of the subject from study participation for any reason).
- After the protocol-defined AE reporting period, unless otherwise noted, only SUSARs as defined in Version 2.0 of the EAE Manual will be reported to DAIDS if the study staff become aware of the events on a passive basis (from publicly available information).

1. STATISTICAL CONSIDERATIONS
   1. General Design Issues

This Phase IV randomized trial is designed to compare the frequency of immunologic deterioration over **28** weeks of treatment of non- or partly non-adherent subjects randomized to one of two different strategies: a monotherapy bridge (3TC or FTC) or continuation of HAART. Secondary objectives include comparing changes in genotypic and phenotypic drug resistance, changes in HIV replication capacity from baseline to **week 28**, as well as differences in adherence, and adverse events (proportion with ≥Grade 3 adverse events) for subjects on the two study arms.

Although information is available on the efficacy of these treatments when properly implemented, the question of relative efficacy of the two strategies in non-compliant subjects is unknown. Secondary analysis of factors predictive of immunologic failure in the two arms will also provide helpful guidance in choosing a strategy for a given individual.

- 1. Outcome Measures
     1. Primary Outcome Measures

Immunologic deterioration will be declared for a subject if any one of the following conditions is observed within the first **28** weeks:

- ≥ 30% decline in absolute CD4+ T cell count, or
- development of CDC class C events.
  - 1. Secondary Outcome Measures

Secondary outcomes include:

- HIV drug resistance genotype and phenotype
- CD4% and CD4+ T cell count
- HIV-1 RNA levels
- Measures of immune activation including: CD8+/CD38+/HLA-DR+ T lymphocytes and hsCRP
- Cardiovascular risk factors including HDL and LDL cholesterol
- HIV replication capacity as measured by a change in the replication capacity measure
- New ≥ Grade 3 signs, symptoms and laboratory values
- Development of adverse clinical outcomes including new bacterial infections, hospitalizations, HIV-related and non-HIV-related morbidity
- Adherence as measured by 3-day recall
  1. Randomization and Stratification

Subjects will be randomized to receive 3TC or FTC monotherapy or continue their current HAART regimen for **28** weeks. Randomization will be 1:1, using a dynamic permuted block system with institutional balancing. Randomization will be stratified by CD4+ T cell count (<400 cells/mm^3^ versus ≥400 cells/mm^3^) to assure approximate balance between study arms with respect to distributions of initial immunologic condition; there will be no accrual limits for the CD4+ T cell count strata.

- 1. Sample Size and Accrual

Based on data from Castagna and Opravil, we expect that fewer than 50% of subjects will experience immunologic failure over **28** weeks. Sample size calculations were made using PASS 2002 (52). The sample size has been chosen to provide 80% power to detect a difference of 0.15 between study arms in proportions of subjects with immunologic failure using a log-rank test with a 2-sided Type I error rate of 0.05. Assuming a 10% rate of loss to follow-up and proportional hazards, a sample size of 344 subjects (172/arm) provides 80% power when the failure rates are 0.25 and 0.40, while providing acceptable power if loss to follow-up or failure rates turn out to be higher than expected (see Table 8.1; P1 denotes the proportion experiencing immunologic failure on the inferior arm, and P2 denotes the proportion experiencing immunologic failure on the better arm).

| Table 8.1: Power to detect a 0.15 difference in failure rates between study arms using Kaplan-Meier estimates when N=344 | | | |
| --- | --- | --- | --- |
| P1 | P2 | Loss to f/u | Power |
| 0.60 | 0.45 | 10% | 0.77 |
|  |  | 15% | 0.74 |
| 0.50 | 0.35 | 10% | 0.76 |
|  |  | 15% | 0.74 |
| 0.40 | 0.25 | 10% | 0.80 |
|  |  | 15% | 0.78 |

Hazard ratios for the failure probability configurations given above are, by row: 1.78, 1.60, and 1.53.

*Accrual*

The total estimated sample size to be accrued to the study is 344; 172/arm. Although it is an estimate, based on the October 2009 site survey of domestic and international sites, site investigators identified approximately 1100 patients that they felt would be eligible for a bridging protocol. **The protocol has been amended to diminish barriers to enrollment related to the baseline CD4 eligibility threshold.  This change is expected to yield a monthly enrollment rate of from 5-10 patients per month, implying accrual of the desired sample size in 3-6 years.**

- 1. Monitoring
     1. Routine Monitoring

Summaries of AEs (Grade 3 or 4) as well as study conduct (in terms of data completeness) will be reviewed on a regular basis by the protocol core team (Chairs, Medical Officers, Statistician, Data Manager and Clinical Trials Specialist). Summaries will be pooled across treatment arms. The study team will not review off-treatment or off-study rates.

Accrual to this study will be monitored by the IMPAACT leadership in accordance with standard operating procedures. In addition, the team will monitor feasibility quarterly, first based on site protocol registration and then on accrual. Initially, the team will monitor site protocol registration quarterly to ensure that an adequate number of sites have registered to complete the protocol. **Slow accrual within the first 8 months of protocol implementation led to an amendment to allow more individuals with low CD4 counts to enroll.  Once the amended version has been registered, accrual will be monitored. One year after 15 sites have registered to the amended protocol, if the sample size is not at least 50, the team will assess the reasons for continued slow accrual and will modify or terminate protocol as appropriate.**

**The** full protocol monitoring plan **contains** more specific details.

- - 1. Interim Analyses

This study will also be monitored by a NIAID-sponsored Data and Safety Monitoring Board (DSMB).  The DSMB will review information concerning accrual, characteristics of subjects, quality and completeness of data collection, retention, and adverse events at least every six months after the first subject is randomized.  Based on the 2-3 year accrual expectation for the study, it is anticipated that the study will undergo 4-6 reviews by the DSMB.  The first will occur within six months after the accrual of the first subject.  Since accumulated efficacy data will be limited at the time of the initial review, although summaries of the efficacy data will be presented, formal review will be limited to an evaluation of study safety and conduct. Data provided will include the number of enrollees, distribution across sites, summary of entry statistics, data completeness, adverse events and the number of subjects who are evaluable for the primary outcome measure of immunologic deterioration. The interim analysis schedule may be modified if needed based on actual accrual rates or if recommended by the DSMB.

One interim efficacy analysis of the primary treatment comparisons will be conducted when half the expected number of primary outcome measure have occurred. Assuming failure rates of 25% and 40% for the two study arms and a target number of evaluable subjects of 310, this would occur when 51 subjects have experienced treatment failure.

Repeated group sequential testing for differences between groups will be conducted. The Haybittle-Peto guideline requires a nominal significance level of <0.001 at the interim analysis in order for stopping to be considered. If one randomized arm is shown to be significantly inferior to the other arm using the Haybittle-Peto rule, then consideration may be given to recommending termination of the inferior arm or the study as a whole.

Simulation of the interim monitoring process has been performed to understand the capacity of the procedure to detect unexpected differences between abilities of subjects taking the study treatments to avoid immunologic deterioration. The probability of a significant result under the Haybittle-Peto rule applied at 51 total events is only 10% if the failure rates are 25% and 40%, but if the failure rates are 25% and 50%, the probability of a significant interim result is 38%; for rates 25% and 60% the probability of a significant interim result is 73%, and for rates 25% and 70% the probability of a significant interim result is 93%. Thus the interim monitoring plan formally protects, with high probability, subjects from enduring a 2.4-2.8 fold elevated risk of immunologic deterioration should one of the study regimens present such a risk. The proposed design with monitoring does not yield high probability of detecting a doubling of risk (the contrast of 30% vs. 60% is detected only with probability 51%, and we have seen that 25% vs. 50% is detected with probability 38%).

At the interim analysis, the DSMB will also consider the ability of the study to achieve its objectives.

- 1. Analyses

The primary assessments of all objectives will be conducted as intent-to-treat analyses. Primary analyses will average over baseline immunologic strata within arms, while additional exploratory analyses will consider the possibility of interaction between baseline immunologic stratum and treatment effects.

- - 1. Primary Objective (immunologic failure):

The primary objective of this study is to use **28** weeks of observation of randomized subjects to compare two strategies to treatment of non- or partly non-adherent individuals failing HAART with respect to their capacity to protect subjects from immunologic deterioration. The analytic framework will be time-to-event: Kaplan-Meier curves and large-sample tests of equivalent distributions of “deterioration-free time’’ will be used to compare the treatment arms with respect to protection from risk of immunologic failure, which is defined to occur whenever any one of the following conditions is observed: a ≥ 30% decline in absolute CD4+ T cell count or development of CDC class C events. The log-rank test will be used to test the null hypothesis of equivalent distributions of deterioration-free time on the two arms. The probability of avoiding immunologic deterioration to **28** weeks will be estimated via the method of Kaplan and Meier, and the 95% confidence intervals (CIs) for this probability will be computed for each arm using Greenwood’s formula. The primary assessment of this objective will be conducted as an intent-to-treat analysis.

- - 1. Step 1, Secondary Objective #1 (drug resistance)

The changes in genotypic HIV drug resistance will be compared between the two study arms by comparing the within-subject change in the pattern of reduced drug susceptibility that is predicted by genotypic testing from baseline to **week 28** in the two study arms. The statistical tests used for analysis of resistance will vary depending on the variable(s) compared (e.g., number of drugs with reduced susceptibility, type and number of mutations, level of susceptibility to 3TC).

- - 1. Step 1, Secondary Objective #2 (dynamics of immunologic deterioration)

The slopes for CD4+ T cell count decline in the two arms will be compared using mixed effects models allowing subject-specific departures from cohort-averaged trajectories in CD4+ T cell count and differential follow-up time in case of censoring due to drop out or termination of study treatment owing to observation of immunologic deterioration.

- - 1. Step 1, Secondary Objective #3 (predictors of immunologic failure)

Proportional hazards regression will be used to calculate hazard ratios and corresponding CIs for parameters of models that employ demographic and clinical variables (including intake CD4+ T cell count stratum, number of prior ARVs, and extent of prior resistance) for prediction of immunologic failure.

- - 1. Step 1, Secondary Objective #4 (change in viral load/CD4+ T cell count)

Within-subject changes in log_10_ HIV viral load and CD4+ T cell count from baseline to **week 28** will be compared between the two arms using 2‑sample t‑tests. Mean CD4+ T cell count and log_10_ HIV viral load over time will be plotted by arm.

- - 1. Step 1, Secondary Objective #5 (change in immune activation)

Within-subject changes in percentage of CD8+/CD38+/HLA-DR+ T lymphocytes, hsCRP, LDL and HDL cholesterol from baseline to **week 28** will be compared between the two study arms using 2-sample t-tests. Means over time will be plotted by arm.

- - 1. Step 1, Secondary Objective #6 (adverse events)

The number and percent of subjects with grade 3 or higher adverse events will be compared by study arm using Fisher’s exact test. Times to first grade 3 or higher event in the two study arms will be compared using log-rank tests.

- - 1. Step 1, Secondary Objective #7 (safety)

The number and percent of subjects with adverse clinical outcomes will be compared by study arm using Fisher’s exact test.

- - 1. Step 1, Secondary Objective #8 (adherence)

Three-day recall of adherence will be categorized. The proportion of subjects at **week 28** that are in the different categories of adherence, as measured by 3-day recall, will be compared between the two study arms using chi-square tests. Rates of adherence will be plotted over time by arm.

- - 1. Step 1, Secondary Objective #9 (change in phenotypic HIV drug resistance and HIV replication capacity)

Within-subject change in phenotypic HIV drug resistance and HIV replication capacity from baseline to **week 28** will be compared between the two arms using 2-sample t‑tests.

- - 1. Step 2, Secondary Objective #1 (immunologic failure)

Probabilities of immunologic deterioration and corresponding 95% CIs will be calculated for subjects on each study arm using Kaplan-Meier estimates, as well as a CI on the difference in probabilities between the two arms at **40** and **52** weeks. The log-rank test will be used to test the null hypothesis of equivalent distributions of deterioration-free time on the two originally assigned treatment arms. The primary assessment of this objective will be conducted as an intent-to-treat analysis.

- - 1. Step 2, Secondary Objective #2 (dynamics of immunologic deterioration)

The slopes for CD4+ T cell count decline in the two originally assigned treatment arms will be compared over **52** weeks using mixed effects models allowing subject-specific departures from cohort-averaged trajectories in CD4+ T cell count and differential follow-up time in case of censoring due to drop out or termination of study treatment owing to observation of immunologic deterioration.

- - 1. Step 2, Secondary Objective #3 (drug resistance)

The changes in genotypic HIV drug resistance will be compared between the two originally assigned treatment arms by comparing the within-subject change in the pattern of reduced drug susceptibility that is predicted by genotypic testing or measured by phenotypic testing from baseline to week **52** in the two study arms. The statistical tests used for analysis of resistance will vary depending on the variable(s) compared (e.g., number of drugs with reduced susceptibility, type and number of mutations, level of susceptibility to 3TC).

- - 1. Step 2, Secondary Objective #4 (adherence)

Three-day recall of adherence will be categorized. The proportion of subjects at weeks **40** and **52** that are in the different categories of adherence, as measured by 3-day recall, will be compared between the two originally assigned treatment arms using chi-square tests. Rates of adherence will be plotted over time by arm.

- - 1. Step 2, Secondary Objective #5 (change in immune activation)

Within-subject changes in percentage of CD8+/CD38+/HLA-DR+ T lymphocytes, hsCRP, LDL and HDL cholesterol from baseline to week **52** will be compared between the two originally assigned treatment arms using 2-sample t-tests. Means over time will be plotted by arm.

- - 1. Step 2, Secondary Objective #6 (change in viral load/CD4+ T cell count)

Within-subject changes in log_10_ HIV viral load and CD4+ T cell count from baseline to **week 52** will be compared between the two originally assigned treatment arms **using** 2‑sample t‑tests. Mean CD4+ T cell count and log_10_ HIV viral load over time will be plotted by arm.

**To supplement the primary intent to treat analysis, we will include enumeration of regimen changes occurring between Step 1 and Step 2 and will include indicators of Step 1-Step2 regimen change in models for change from baseline in the key immunologic and virologic parameters.**

- - 1. Step 2, Secondary Objective #7 (adherence)

Three-day recall of adherence will be categorized. The proportion of subjects at week **52** that are in the different categories of adherence, as measured by 3-day recall, will be compared between the two originally assigned treatment arms using chi-square tests. Rates of adherence will be plotted over time by arm.

1. HUMAN SUBJECTS
   1. Institutional Review Board and Informed Consent

This protocol, the informed consent document (Appendix V), and any subsequent modifications must be reviewed and approved by the IRB or EC responsible for oversight of the study. Written informed consent must be obtained from the subject (or parents or legal guardians of subjects who cannot consent for themselves, such as those below the legal age). The subject's assent must also be obtained if he or she is able to understand the nature, significance, and risks of the study. The informed consent will describe the purpose of the study, the procedures to be followed, and the risks and benefits of participation. A copy of the consent form will be given to the subject (or parent or legal guardian).

Each site which receives US HHS funding and follows the United States Code of Federal Regulations Title 45-Public Welfare, Part 46-Protection of Human Subjects (also known as the Common Rule) should have on record at the site a plan that detects and addresses any change in guardianship occurring in pediatric subjects and determines when a study subject must have a consent process which involves a legally authorized representative (LAR) other than a family member with guardianship. The plan will include how the site determines when a LAR is initially or no longer needed and how frequently the LAR re-signs the consent. The plan should follow all IRB/EC, local, state, national and/or host country guidelines. Confirmation of such a plan at a site should be submitted with protocol registration materials.

- 1. Subject Confidentiality

All laboratory specimens, evaluation forms, reports, and other records will be identified only by a coded number to maintain subject confidentiality. All records will be kept in a secured area. All computer entry and networking programs will be done with coded numbers only. Clinical information will not be released without written permission of the subject or the subject’s parent or legal guardian, except as necessary for monitoring by the Office for Human Research Protections (OHRP), the NIH, the local IRB or Ethics Committee, local regulatory authorities.

- - 1. Confidential Testing Results for Adolescents

This protocol is focused on youth who will span the ages when a parent/guardian will usually be granting permission for their participation (8-17 years old) through older ages when the youth will usually consent for themselves (18-24 years old).  Questions and testing related to pregnancy and sexual activity are included in the protocol. These elements are important aspects of maximizing subject safety (contraception, pregnancy). Adolescents are entitled to confidential testing and care for reproductive health in many jurisdictions, and, in general, access to confidential care is thought to improve the ability for adolescents to access this care. As a result, information collected in this study (including for screening for this study) related to pregnancy and sexual activity will not be shared with parents (or other adults consenting for youth’s participation) without permission of the youth, and study staff will ensure that the subject is referred to his or her medical provider for appropriate counseling and management if problems in these areas are identified. However, since local guidelines, practice and regulations may vary, instructions to sites appear in the sample informed consent to emphasize that sites may have to adapt this approach, e.g., “[*Sites should modify the preceding language about confidentiality of sexual activity and pregnancy test results to conform to their local practice, regulations and IRB/EC requirements.*]”

- 1. Study Discontinuation

The study may be discontinued at any time by the NIAID, the IRB or EC, the Office for Human Research Protections (OHRP), or other governmental agencies as part of their duties to ensure that research subjects are protected.

1. PUBLICATION OF RESEARCH FINDINGS

Publication of the results of this trial will be governed by IMPAACT policies. Any presentation, abstract, or manuscript will be made available for review by the pharmaceutical sponsors prior to submission.

1. BIOHAZARD CONTAINMENT

As the transmission of HIV and other bloodborne pathogens can occur through contact with contaminated needles, blood, and blood products, appropriate blood and secretion precautions will be employed by all personnel in the drawing of blood and shipping and handling of all specimens for this study, as currently recommended by the Centers for Disease Control and Prevention.

All infectious specimens will be sent using the ISS-1 SAF-T-PAK mandated by the International Air Transport Association Dangerous Goods Regulations-Packing Instruction 602. Refer to individual carrier guidelines (e.g., Federal Express or Airborne) for specific instructions.

1. REFERENCES

APPENDIX I

SCHEDULE OF EVALUATIONS

|  |  | Step 1 | | | | | Step 2 | | | Step 1 or Step 2 | | | |
| --- | --- | --- | --- | --- | --- | --- | --- | --- | --- | --- | --- | --- | --- |
| Event | Screening^1^ | Step 1 Entry  (Week 0) | Week 4 ±**14** days | Week 12 ±**14** days | Week **20**  ±**28** days | **Week 28**  ±**28** days | Step 2 Entry^17^ | Week **40**  ±**28** days  **(12 weeks after**  **step 2 entry)** | Week **52**  ±**28** days  **(24 weeks after**  **step 2 entry)** | Change in treatment regimen occurring **before week 28^18^** | 24 weeks after starting new HAART**^22^** | Early Discontinuation**^23^** |  |
| CLINICAL EVALUATIONS |  |  |  |  |  |  |  |  |  |  |  |  |  |
| Informed Consent | X |  |  |  |  |  |  |  |  |  |  |  |  |
| History^2^ | X | X | X | X | X | X | X | X | X | X | X | X |  |
| Physical exam^3^ | X | X | X | X | X | X | X | X | X | X |  | X |  |
| Adherence Questionnaire | X | X | X | X | X | X | X | X | X | X |  | X |  |
| Offer co-enrollment to P1074 (domestic sites only) | X | X | X | X | X | X | X | X | X | X |  |  |  |
| LABORATORY EVALUATIONS |  |  |  |  |  |  |  |  |  |  |  |  |  |
| Hematology^4^ | 1 mL | 1 mL | 1 mL | 1 mL | 1 mL | 1 mL |  | 1 mL | 1 mL |  | 1 mL**^21^** | 1 mL |  |
| Chemistry^5^ | 2 mL | 2 mL |  | 2 mL |  | 2 mL |  | 2 mL | 2 mL |  | 2 mL**^21^** | 2 mL |  |
| Lipid profile (fasting)^6^ |  | 1 mL |  |  |  | 1 mL |  |  | 1 mL |  |  | 1 mL |  |
| Pregnancy test (serum or urine)^7^ | X | X |  | X |  | X | X | X | X |  |  |  |  |
| *Virology* |  |  |  |  |  |  |  |  |  |  |  |  |  |
| Real-time plasma HIV-1 RNA^8^ | 5 mL | 5 mL | 5 mL | 5 mL |  | 5 mL | 5 mL | 5 mL | 5 mL | 5 mL | 5 mL | 5 mL |  |
| HIV genotyping to document presence of the M184V mutation, if prior documentation is not available^9^ | 6 mL |  |  |  |  |  |  |  |  |  |  |  |  |
| HIV genotyping  (ViroSeq, real-time) ^10^ |  | 6 mL |  | 6 mL |  | 6 mL |  |  | 6 mL | 6 mL**^19^** |  | 6 mL**^19^** |  |
| HIV phenotyping with replication capacity (retrospective/batched)^11^ |  | 6 mL |  |  |  | 6 mL |  |  | 6 mL | **[6 mL] ^20^** |  |  |  |
| Stored plasma for quality control testing, resistance testing and viral characterization^12^ |  | 8 mL |  | 8 mL | 6 mL | 8 mL | 6 mL | 6 mL | 8 mL | 6 mL |  | 6 mL |  |
| Stored cell pellet for host genetic testing^13^ |  | X |  |  |  |  |  |  |  |  |  |  |  |
| *Immunology* |  |  |  |  |  |  |  |  |  |  |  |  |  |
| Lymphocyte subsets^14^ | 1 mL | 1 mL | 1 mL | 1 mL | 1 mL | 1 mL | 1 mL | 1 mL | 1 mL | 1 mL | 1 mL | 1 mL |  |
| Inflammatory/activation markers [plasma and PBMC in EDTA (purple top)]^15^ |  | 10 mL |  | 10 mL |  | 10 mL |  |  | 10 mL | 10 mL |  | 10 mL |  |
| Inflammatory/activation markers [plasma in NaCitrate (blue top)]^15^ |  | 2.7 mL |  | 2.7 mL |  | 2.7 mL |  |  | 2.7 mL | 2.7 mL |  | 2.7 mL |  |
| Stored plasma and PBMCs^16^ |  | X |  | X |  | X |  |  | X |  |  | X |  |
| TOTAL BLOOD VOLUMES | 15-16 mL | 42.7-43.7 mL | 7 mL | 35.7-36.7 mL | 8 mL | 42.7-43.7 mL | 12-13 mL | 15-16 mL | 42.7-43.7 mL | 30.7-**36.7** mL | 6-9 mL | 34.7 mL |  |

APPENDIX I (Cont.)

1. Screening evaluations must be performed within 30 days of Entry.
2. Antiretroviral history [list of all prior ARV medications, start/stop dates for all prior regimens, and start date for the current HAART regimen ONLY at screening, and for ARV medications during the study], concomitant medications, all prior/documented genotypic resistance mutations; nadir CD4+ T cell count and demographics (ONLY at screening).
3. Physical exam includes height and weight, signs and symptoms, interim diagnoses and hospitalizations.
4. Hematology must include complete blood count (CBC) with differential and platelet count.
5. Blood chemistries must include creatinine, AST, and ALT. Lipase is required at screening, **week 28** and **52**.
6. Must be collected in a fasting state (no food or drink except water for 8 hours prior to specimen collection) and must include triglycerides, total cholesterol, LDL, and HDL.
7. Pregnancy test (serum or urine): Required at screening, Step 1 entry, week 12, **week 28**, Step 2 entry, week **40**, week **52** and any time pregnancy is suspected for females who are of child-bearing potential. If blood test is performed collect 1.0 mL in a red top tube.
8. To be tested at a local CLIA-certified laboratory (U.S. sites), or a laboratory approved by the network’s Central Laboratory (non-U.S. sites) using the same FDA-approved HIV-1 real-time assay throughout the study. Approval from the Protocol Chair and the Protocol Virologist is required to change from one assay to another during the study. **(Note: If a subject has any viral load result that would mandate stopping randomized treatment (Section 6.3), randomized treatment is only stopped if both the original and confirmatory values meet the stopping criterion. Once viral load is confirmed and the subject is deemed to have met criteria for stopping randomized treatment, subjects on Step 1 will be asked to proceed to Step 2. All subjects will continue to be followed according to the scheduled Step 2 study visits and undergo all study procedures and tests listed for those visits.)**
9. At screening, confirmation of M184V is performed for international sites only if HIV genotyping has not been performed previously. HIV genotyping at screening will be performed at a local/regional laboratory approved by the Protocol Virologist**.** Testing may be performed using a validated home-brew assay. **At screening for U.S. sites, genotyping should be performed to document presence of the M184V mutation if not done previously. (Note:  Domestic sites will have to pay for the screening genotype.  The protocol will only be paying for international sites where M184 has not been previously documented.)**
10. At enrollment and the indicated post-enrollment visits, HIV genotyping will be performed in real-time at a local/regional laboratory (e.g. Specialty Lab in the US, WITS lab in SA). The laboratory performing the testing must have a record of successful performance for HIV genotyping in the VQA External Quality Assurance program. The Protocol Virologist may move testing to another laboratory if needed.
11. HIV phenotyping with replication capacity will be performed at Monogram Biosciences, Inc. (South San Francisco, CA, a subsidiary of LabCorp, Inc.). This testing will be performed retrospectively for a subset of participants. Testing will only be performed at **52** weeks for participants who remain on their original assigned treatment regimen.
12. Samples are stored for future, retrospective substudies and for quality control testing (e.g., confirmation of results obtained locally, resolution of testing problems).
13. This evaluation is optional and is only performed in subjects who have provided consent for this testing. Cell pellets will be prepared from whole blood samples after removal of plasma for plasma storage (sample for resistance testing and viral characterization). Cell pellets will be stored without cryopreservation (non-viable cells) for host genetic (DNA) testing. If this sample is not collected at the entry visit, it can be collected at any other visit.
14. Lymphocyte subset blood samples should be collected in EDTA tubes. These samples will be analyzed for CD4/CD8 T-lymphocyte counts and percentages. These evaluations must be performed in laboratories that are certified by the DAIDS-sponsored IQA and CLIA (or equivalent for international sites), and must be performed at the same laboratory throughout the study where possible. If a subject’s CD4+ T lymphocytes count drops 30% or more below the respective Step 1 or Step 2 entry value during the first **28** weeks, the CD4+ T lymphocyte count should be repeated within one to six weeks. **(Note: If a subject has any CD4+ T cell count that would mandate stopping randomized treatment (Section 6.3), randomized treatment is only stopped if both the original and confirmatory values meet the stopping criterion. Once CD4+ T cell value is confirmed and the subject is deemed to have met criteria for stopping randomized treatment, subjects on Step 1 will be asked to proceed to Step 2. All subjects will continue to be followed according to the scheduled Step 2 study visits and undergo all study procedures and tests listed for those visits.)**
15. Whole blood collected in EDTA and sodium citrate (10 mL in purple top and 2.7 mL in blue top) at the site will be processed for plasma storage and **from the EDTA tube ONLY** isolation and cryopreservation of viable PBMC to be used for markers of inflammation and activation (plasma levels of IL-6, TNF-alpha, IFN-alpha, LPS, sCD14, D-Dimer, hsCRP, MCP-1, sVCAM-1, sICAM-1, E selectin, P-selectin, fibrinogen; flow cytometric measurement of CD38, HLA-DR, Ki67, CD95, apoptosis) and repository storage. See Laboratory Processing Chart (LPC) for processing information. Sites will ship cryopreserved specimens at request of protocol team at **the end of the study**. PBMC cryopreservation is required for sites in the US and sites must be certified by IQA for viable PBMC cryopreservation. PBMC cryopreservation is optional for non-US sites and may be done if they are certified by the IQA or equivalent for viable PBMC cryopreservation.
16. Remaining plasma**,** **from EDTA and Sodium Citrate tubes,** and PBMC specimens, **EDTA tubes ONLY,** from the inflammatory/activation markers specimen draw will be stored at the **sites until shipping according to LPC instructions**.
17. For Step 2 subjects may: (a) continue randomized treatment, (b) begin a new HAART regimen, if the clinical team including the subject, and or caregiver (as appropriate) determine that it is clinically necessary or adherence is likely, or (c) discontinue study treatment, but remain on study follow-up. This decision will be made by the subject and their clinical team and not the study team. Entry into Step 2 **can** occur **any time after entry up to week 34**.
18. **All subjects who discontinue the study-assigned regimen prior to week 28 will be asked to enroll into Step 2. The “Change in Treatment Regimen Occurring Before Week 28 visit” and the “Step 2 entry visit” can be combined. The window for this visit will be +/- 2 weeks around the discontinuation date, thus any evaluations done in the 2 weeks preceding the discontinuation date can be used for this visit. Subjects will have two follow-up visits, one at 12 weeks following entry into Step 2 (Week 40 in the Schedule of Evaluations) and 24 weeks following entry into Step 2 (Week 52 in the Schedule of Evaluations). These subjects will undergo all study procedures and tests listed for those visits.**
19. **For subjects changing treatment regimen or discontinuing study treatment prior to week 28, provided that the last study visit occurs more than 8 weeks after the last real-time HIV genotyping test was performed.**
20. **This evaluation should not be repeated if it was collected within the past 14 days.**
21. **These evaluations are only collected if not performed within the past 28 days.**
22. **Subjects whose 24^th^ week after initiating a new HAART regimen is after study week 52 will have medication history, plasma HIV RNA, Lymphocyte subset data obtained by data sharing with P1074 for the subjects who have co-enrolled. No blood draw will be done for these subjects.**
23. **These evaluations will be performed at the time the subject is discontinued from study if occurring at times other than 28 or 52 weeks.**

For insufficient blood draws, priorities are as follows (see Appendix IV for guidance regarding blood volume collection in small children):

1. Lymphocyte subsets
2. Hematology
3. Chemistry
4. Real-time plasma HIV-1 RNA
5. Resistance Testing/Special Virology
6. Inflammatory/activation markers

APPENDIX II
CDC CATEGORY C HIV CLASSIFICATION SYSTEM (FOR CHILDREN <13 YEARS OF AGE)*

| Children who have any condition listed in the 1987 surveillance case definition for acquired immunodeficiency syndrome (below), with the exception of LIP (which is a category B condition)   - Serious bacterial infections, multiple or recurrent (i.e., any combination of at least two culture-confirmed infections within a 2-year period), of the following types: septicemia, pneumonia, meningitis, bone or joint infection, or abscess of an internal organ or body cavity (excluding otitis media, superficial skin or mucosal abscesses, and indwelling catheter-related infections) - Candidiasis, esophageal or pulmonary (bronchi, trachea, lungs) - Coccidioidomycosis, disseminated (at site other than or in addition to lungs or cervical or hilar lymph nodes) - Cryptococcosis, extrapulmonary - Cryptosporidiosis or isosporiasis with diarrhea persisting >1 month - Cytomegalovirus disease with onset of symptoms at age >1 month (at a site other than liver, spleen, or lymph nodes) - Encephalopathy (at least one of the following progressive findings present for at least 2 months in the absence of a concurrent illness other than HIV infection that could explain the findings): a) failure to attain or loss of developmental milestones or loss of intellectual ability, verified by standard developmental scale or neuropsychological tests; b) impaired brain growth or acquired microcephaly demonstrated by head circumference measurements or brain atrophy demonstrated by computerized tomography or magnetic resonance imaging (serial imaging is required for children <2 years of age); c) acquired symmetric motor deficit manifested by two or more of the following: paresis, pathologic reflexes, ataxia, or gait disturbance - Herpes simplex virus infection causing a mucocutaneous ulcer that persists for >1 month; or bronchitis, pneumonitis, or esophagitis for any duration affecting a child >1 month of age - Histoplasmosis, disseminated (at a site other than or in addition to lungs or cervical or hilar lymph nodes) - Kaposi's sarcoma - Lymphoma, primary, in brain - Lymphoma, small, noncleaved cell (Burkitt's), or immunoblastic or large cell lymphoma of B-cell or unknown immunologic phenotype - Mycobacterium tuberculosis, disseminated or extrapulmonary - Mycobacterium, other species or unidentified species, disseminated (at a site other than or in addition to lungs, skin, or cervical or hilar lymph nodes) - Mycobacterium avium complex or Mycobacterium kansasii, disseminated (at site other than or in addition to lungs, skin, or cervical or hilar lymph nodes) - Pneumocystis jiroveci pneumonia - Progressive multifocal leukoencephalopathy - Salmonella (nontyphoid) septicemia, recurrent - Toxoplasmosis of the brain with onset at >1 month of age - Wasting syndrome in the absence of a concurrent illness other than HIV infection that could explain the following findings: a) persistent weight loss >10% of baseline; OR b) downward crossing of at least two of the following percentile lines on the weight-for-age chart (e.g., 95th, 75th, 50th, 25th, 5th) in a child ≥1 year of age; OR c) <5th percentile on weight-for-height chart on two consecutive measurements, ≥30 days apart PLUS 1) chronic diarrhea (i.e., ≥ two loose stools per day for >30 days), OR 2) documented fever (for ≥30 days, intermittent or constant) |
| --- |

* Source: Guidelines for the Use of Antiretroviral Agents in Pediatric HIV Infection, February 23, 2009, <http://www.aidsinfo.nih.gov/ContentFiles/PediatricGuidelines.pdf>

APPENDIX III

CDC CATEGORY C HIV CLASSIFICATION SYSTEM (FOR ADOLESCENTS AND YOUNG ADULTS ≥13 YEARS OF AGE)*

- Bacterial pneumonia, recurrent (≥2 episodes in 12 months)
- Candidiasis of the bronchi, trachea, or lungs
- Candidiasis, esophageal
- Cervical carcinoma, invasive, confirmed by biopsy
- Coccidioidomycosis, disseminated or extrapulmonary
- Cryptococcosis, extrapulmonary
- Cryptosporidiosis, chronic intestinal (>1-month duration)
- Cytomegalovirus disease (other than liver, spleen, or nodes)
- Cytomegalovirus retinitis (with loss of vision)
- Encephalopathy, HIV-related
- Herpes simplex: chronic ulcers (>1-month duration), or bronchitis, pneumonitis, or esophagitis
- Histoplasmosis, disseminated or extrapulmonary
- Isosporiasis, chronic intestinal (>1-month duration)
- Kaposi sarcoma
- Lymphoma, Burkitt, immunoblastic, or primary central nervous system
- Mycobacterium avium complex (MAC) or M kansasii , disseminated or extrapulmonary
- Mycobacterium tuberculosis, any site, pulmonary or extrapulmonary
- Mycobacterium , other species or unidentified species, disseminated or extrapulmonary
- Pneumocystis jiroveci (formerly carinii ) pneumonia (PCP)
- Progressive multifocal leukoencephalopathy (PML)
- Salmonella septicemia, recurrent (nontyphoid)
- Toxoplasmosis of brain
- Wasting syndrome due to HIV (involuntary weight loss >10% of baseline body weight) associated with either chronic diarrhea (≥2 loose stools per day ≥1 month) or chronic weakness and documented fever ≥1 month

* Source: 1993 Revised Classification System for HIV Infection and Expanded Surveillance Case Definition for AIDS Among Adolescents and Adults, MMWR, December 18, 1992/41(RR-17), <http://www.cdc.gov/mmwr/preview/mmwrhtml/00018871.htm>

APPENDIX IV

BLOOD VOLUME COLLECTION IN SMALL CHILDREN

Recommendations as to the acceptable amount of blood to be drawn from subjects specifically for research purposes are variable and largely depend on individual institutions. The National Institutes of Health (NIH) guidelines state that no more than

**5** mL/kg are to be drawn **on** a single **day** and no more than 9.5 mL/kg are to be drawn over any eight**-**week period for the purposes of research in children.

Example: An 8 year old girl weighing 22kg (15% weight-for-age) has an approximate blood volume of 1540mL (70mL/kg total blood volume x 12kg). A 21 mL blood draw represents approximately 1.4% of total blood volume for this subject. Alternatively, this represents 0.95mL/kg volume of blood drawn in a 24 hour period, well below the **5** mL/kg recommended by the NIH.

In the case where a seemingly eligible subject has had blood drawn, or will have blood drawn for any reason, such that the total volume blood being drawn over any 8 week period will exceed 9.5 mL/kg, the subject will be considered ineligible for this study.

APPENDIX V

DIVISION OF AIDS

INTERNATIONAL MATERNAL PEDIATRIC ADOLESCENT AIDS CLINICAL TRIALS GROUP (IMPAACT)

### SAMPLE INFORMED CONSENT

For protocol:

P1094, “Evaluation of 3TC or FTC Monotherapy Compared to Continuing HAART as a Bridging antiretroviral Strategy in persistently non-adherent Children, Adolescents, and young adults who are failing haart and have the M184V Resistance Mutation”

**VERSION 2.0, DATED MARCH 01, 2012**

SHORT TITLE FOR THE STUDY:

P1094, Evaluation of 3TC or FTC Monotherapy Compared to Continuing HAART as a Bridging Strategy

INTRODUCTION

You are/your child is being asked to take part in this research study because you are/your child is infected with human immunodeficiency virus (HIV), the virus that causes Acquired Immune Deficiency Syndrome (AIDS), and because you have/your child has not taken the medications in a way that controls the virus. This study is sponsored by the National Institutes of Health (NIH). The doctor in charge of this study at this site is: (insert name of Principal Investigator). Before you decide if you want to be/want your child to be a part of this study, we want you to know about the study.

This is a consent form. It gives you information about this study. The study staff will talk with you about this information. You are free to ask questions about this study at any time. If you agree to allow your child to take part in this study, you will be asked to sign this consent form. You will get a copy to keep.

WHY IS THIS STUDY BEING DONE?

Living with a chronic disease such as HIV and having to take/having your child take medications as prescribed by your/your child's doctor can be challenging. It is common that patients will not take these medications properly. But when the medications are not taken properly, they do not work as well. They may stop controlling your/your child’s HIV, which can allow the disease to get worse.

You are/your child is being asked to participate in this study because you are/your child is on a highly active antiretroviral therapy (HAART) regimen (group of HIV medications) that is not controlling your/your child’s HIV.  This is sometimes called a “failing” regimen.  One of the most common reasons for having treatment failure is not taking all of the HIV medications correctly. We know that the HIV can become worse if you/your child stops taking medication altogether. Staying on the same failing HAART regimen or switching to a new HAART regimen and continuing to not take it correctly may increase the risk of developing HIV resistance leading to fewer HIV treatment choices, decreasing immune function, and/or becoming ill. You/your child could switch to a new HAART regimen, but if you/your child do/does not take these medications properly, the same problems can arise again.  There are only a limited number of choices for HAART regimens, so your doctor will be very careful about when to change regimens. This study aims to help figure out a treatment strategy while you/your child work on improving your ability to take your/your child’s medicines correctly. If you/your child participate(s) in this study, you/your child may be assigned to stop taking HAART and begin taking the HIV drugs lamivudine (3TC) or emtricitabine (FTC) alone.  There are risks to taking 3TC/FTC alone (decreasing immune function, increasing viral load, and/or becoming ill).  These risks may occur even if 3TC/FTC are taken properly.  As 3TC/ FTC can be given once a day, it might be easier to keep up with a 3TC/FTC regimen because it involves fewer pills and may have fewer side effects for some people.  However, no studies have been done to compare taking 3TC/FTC alone to continuing on a failing HAART regimen in children and adolescents who are not ready or able to start a new HAART regimen.

The purpose of this study is to compare the use of 3TC or FTC alone vs. continuing a failing HAART regimen in HIV infected children, adolescents and young adults. The study will see if there are changes in the HIV virus and if there is a difference in immune function, viral load and medication side effects between the two groups over **28** weeks. You/your child will be assigned to either take 3TC/FTC alone or continue on his/her current failing HAART regimen. During the first **28** weeks of this study, if you are/your child is randomized to the continue HAART arm, you/your child will not be switched to a different or new, potentially suppressive HAART regimen, but will continue on your current failing HAART regimen. **However, if continuing HAART, you/your child may be switched to a new regimen if you and your/your child's provider feels that it is clinically needed or you/your child meet(s) certain study endpoints (e.g., drop in CD4, increase in viral load).**

At the end of **28** weeks, you/your child will have the choice of remaining on your assigned study group medication(s) or starting a new HAART regimen prescribed by your doctor. Then, you/your child will be followed for another 24 weeks to see if there are changes in your/your child’s virus and to compare the difference in immune function, viral load and medication side effects between the different groups.

Your/your child’s provider will continue to provide the best support to improve your/your child’s adherence during the study.

WHAT DO I/DOES MY CHILD HAVE TO DO IF I AM/HE/SHE IS IN THIS STUDY?

If you agree to participate/allow your child to participate in this study, you/your child will be asked to come to the clinic at least **10** times over **52** weeks, as indicated on the P1094 Subject Visit Schedule below. This study has two steps, Step 1 and Step 2.

*(Note to sites: You may choose to use this supplemental tool if you think it will be helpful when obtaining consent,)*

|  |  | Step 1 | | | | | Step 2 | | | | |
| --- | --- | --- | --- | --- | --- | --- | --- | --- | --- | --- | --- |
| Event | Screening | Step 1  Entry  (Week 0) | Week  4 | Week  12 | Week  **20** | Week  **28** | Step 2 Entry | Week  **40** | Week  **52** | Change in treatment regimen occurring **before week 28** | 24 weeks after starting new  HAART |
| Informed Consent | X |  |  |  |  |  |  |  |  |  |  |
| History | X | X | X | X | X | X | X | X | X | X | X |
| Physical exam | X | X | X | X | X | X | X | X | X | X |  |
| Adherence Questionnaire | X | X | X | X | X | X | X | X | X | X |  |
| Pregnancy test* | X | X |  | X |  | X | X | X | X |  |  |
| Blood Collected | X | X | X | X | X | X | X | X | X | X | X |
| *Pregnancy test will be performed only on girls who have had their first menstrual period. | | | | | | | | | | | |

After you have read and signed this consent form, you/your child will have the following done at the SCREENING visit to see if you/your child can participate in the study:

- You will be asked questions about your/your child’s medications and medical history.
- You/your child will have a physical exam to assess height, weight, symptoms present, and illnesses you have/your child has had.
- You/your child will be asked questions that will collect information on your/your child’s ability to take medications.
- If you/your child can become pregnant, you/your child will provide a urine or blood sample for a pregnancy test. The test must show that you are/your child is not pregnant in order for you/your child to participate in this study.
- You/your child will have about 3 teaspoons (15-16 mL) of blood drawn:
  - for routine blood tests,
  - to measure your/your child’s HIV viral load and to test how your/your child’s immune system is working, and
  - to see if you have/your child has a specific mutation in your/his/her HIV (M184V), if this information is not available from earlier testing.

This visit will last about 1 hour.

Even if you/your child are/is not eligible to enter the study, the results of the screening tests will be used by the protocol team in their research.

*STEP 1*

ENTRY

Once it is known that you/your child can participate in this study, you/your child will have an entry visit. You/your child will be randomized (assigned by chance, as if by the toss of a coin) to continue on your/his/her current HAART regimen OR to receive 3TC or FTC. You/your child will have an equal chance of being assigned to either treatment group. Depending on which group you are/your child is randomized to, you/your child may be required to stop taking his/her current non-nucleoside reverse transcriptase inhibitor (NNRTI). At this visit the following will be done:

- You will be asked questions about your/your child’s medications and medical history.
- You/your child will be asked questions that will collect information on your/your child’s ability to take medications.
- You/your child will have a physical exam to assess height, weight, symptoms present, and illnesses you have/your child has had.
- If you/your child can become pregnant, you/your child will provide a urine or blood sample for a pregnancy test.
- You/your child will have about 8-9 teaspoons (42.7-43.7 mL) of blood drawn:
  - for routine blood tests,
  - to measure your/your child’s HIV viral load, to test how your/your child’s immune system is working, and to perform other tests to assess your/your child’s immune system,
  - to measure the amount of cholesterol and triglycerides (types of fat) in the blood. [You/your child can not eat or drink anything for 8 hours prior to this blood draw (except for required medications and normal amounts of plain water)],
  - to test your/your child’s HIV for drug resistance,
  - to save some blood to study other features of your/your child’s HIV, and
  - to save some blood to test your/your child’s gene’s.
- You will be informed of the results of routine blood tests, HIV viral load, HIV drug resistance, and how your/your child’s immune system is working.

This visit will last about 1-2 hours.

OPTIONAL GENETICS TESTING- ENTRY

The researchers in this study would like to keep you/your child’s samples from this visit for future research. Future research on these samples will help us understand how HIV causes disease and complications, and how best to treat or prevent HIV infection and its complications. The research will look at how each person’s genetic makeup (your/your child’s DNA) either protects them or puts them at greater risk. This kind of information will be particularly important as scientists work toward a vaccine that could protect people from AIDS. They need specimens from people who have HIV and from those who do not.

The samples for the optional genetics testing will be shipped to the particular NICHD or NIAID repositories located in the United States. The samples will be stored at the repositories until the specimens are requested to be shipped to the testing laboratories. Before being sent to a repository, the researchers will remove the information that would allow the samples or genetic information to be linked to you/your child. During future research, nobody will know that the sample came from you/your child.

There are no plans to give participants the results of the tests performed on their stored samples. You will not receive any information on your/your child’s genetic makeup.

Benefits:  There are no direct benefits to you/your child. You/your child will be helping researchers learn more about how to help people with HIV or at risk of HIV infection.

Risks: The samples would be collected as part of your/your child’s study visit. Allowing the samples to be stored in the repository does not pose a significant risk to you/your child’s privacy because the identifying information will be removed. Your/your child’s name will not be available to the repository or to the scientists who may be doing any future test.

If you refuse permission to store these samples for future research, you can still participate in this study.

By signing this part of the form, you do not give up any rights you would normally have. However, once you/your child’s information has been removed from the samples, it will not be possible for you to withdraw the samples from the repository.

I give permission for the use of my/my child’s stored specimens for the purposes stated in the preceding section (special HIV-related tests).

_________________________                      ___________________________   _________

Participant or Witness Signature                              Date

Participant’s Legal Guardian

Signature

STUDY VISITS (Weeks, 4, 12, **20**, and **28**)

Most of these visits will last about 1-2 hours.

At each visit the following will be done:

- You will be asked questions about your/your child’s medications and medical history.
- You/your child will have a physical exam to assess height, weight, symptoms present, and illnesses you have/your child has had.
- You/your child will be asked questions that will collect information on your/your child’s ability to take medications.

If you/your child can become pregnant, you/your child will provide a urine or blood sample for a pregnancy test at weeks 12 and **28**.

You/your child will have about 1-9 teaspoons (7-43.7 mL) of blood drawn, which will vary at each visit:

- - for routine blood tests (weeks 4, 12, **20**, and **28**),
  - to measure your/your child’s HIV viral load (weeks 4, 12, and **28**) and to test how your/your child’s immune system is working, and to perform other tests to assess your/your child’s immune system (weeks 4, 12, **20**, and **28**),
  - to measure the amount of cholesterol and triglycerides in the blood (**week 28**), [You/your child can not eat or drink anything for 8 hours prior to this blood draw (except for required medications and normal amounts of plain water)],
  - to test your/your child’s HIV for drug resistance (weeks 12 and **28**), and
  - to save some blood to test your/your child’s HIV for drug resistance and to study other features of your/your child’s HIV (weeks 12, **20**, and **28**).

You will be informed of the results of routine blood tests, HIV viral load, HIV drug resistance, and how your/your child’s immune system is working.

*STEP 2*

Once you/your child complete(s) Step 1, you/your child will move to Step 2. In Step 2, you/your child will remain on study for another 24 weeks. During the 24 week follow-up period, you/your child will either:

1. continue on your/their assigned study treatment (continue current HAART regimen OR either 3TC or FTC),
2. change treatment regimens if it is decided by you and/or your/your child’s study doctor that you/your child should change treatment regimens, OR
3. stop study treatment, but remain on study.

Entry

You/your child will enter Step 2 (**any time after entry up to week 34*)*** once blood test results **are available to** determine if your/your child’s HIV has changed. The test results will help you and your/your child’s doctor decide if you/your child should change treatment regimens. At this Step 2 entry visit, the following will be done:

- You will be asked questions about your/your child’s medications and medical history.
- You/your child will be asked questions that will collect information on your/your child’s ability to take medications.
- You/your child will have a physical exam to assess height, weight, symptoms present, and illnesses you have/your child has had.
- If you/your child can become pregnant, you/your child will provide a urine or blood sample for a pregnancy test.
- You/your child will have about 3 teaspoons (12-13 mL) of blood drawn:
  - to measure your/your child’s HIV viral load and to test how your/your child’s immune system is working,
  - to save some blood to test your/your child’s HIV for drug resistance and to study other features of your/your child’s HIV.
- You will be informed of the results of the HIV viral load test, HIV drug resistance, and how your/your child’s immune system is working.

This visit will last about 1½ hours.

STUDY VISITS (Weeks **40** & **52**)

Most of these visits will last about 1-2 hours.

At each visit the following will be done:

- You will be asked questions about your/your child’s medications and medical history.
- You/your child will have a physical exam to assess height, weight, symptoms present, and illnesses you have/your child has had.
- You/your child will be asked questions that will collect information on your/your child’s ability to take medications.
- If you/your child can become pregnant, you/your child will provide a urine or blood sample for a pregnancy test.

You/your child will have about 3-9 teaspoons (15-43.7 mL) of blood drawn, which will vary at each visit:

- - for routine blood tests,
  - to measure your/your child’s HIV viral load, to test how your/your child’s immune system is working, and to perform other tests to assess your/your child’s immune system,
  - to measure the amount of cholesterol and triglycerides in the blood (week **52** only). (You/your child can not eat or drink anything for 8 hours prior to this blood draw (except for required medications and normal amounts of plain water), and
  - to test your/your child’s HIV for drug resistance (week **52** only), and
  - to save some blood to test your/your child’s HIV for drug resistance and to study other features of your/your child’s HIV.

You will be informed of the results of routine blood tests, HIV viral load, HIV drug resistance, and how your/your child’s immune system is working.

Week **52** will be your/your child’s last visit.

CHANGE IN TREATMENT VISIT

If you/your child need(s) to change your/their assigned study treatment (continue current HAART regimen OR either 3TC or FTC) before entering Step 2, you/your child will have a *“change in treatment”* visit. This visit will last about 1 hour.

At this visit the following will be done:

- You will be asked questions about your/your child’s medications and medical history.
- You/your child will be asked questions that will collect information on your/your child’s ability to take medications.
- You/your child will have a physical exam to assess height, weight, symptoms present, and illnesses you have/your child has had.

You/your child may have about **7** teaspoons (30.7 mL **– 36.7 mL**) of blood drawn to:

- - measure your/your child’s HIV viral load,
  - test how your/your child’s immune system is working, to perform other tests to assess your/your child’s immune system, and
  - to test your/your child’s HIV for drug resistance.
  - to save some blood to test your/your child’s HIV for drug resistance and to study other features of your/your child’s HIV.

You will be informed of the results of the HIV viral load test, HIV drug resistance, and how your/your child’s immune system is working.

24 WEEKS AFTER STARTING NEW HAART VISIT

You/your child will have this visit if you/your child’s 24^th^ week after starting a new HAART regimen will occur before study visit week **52**. This visit will last about ½ hour.

At this visit you will be asked questions about your/your child’s medications and medical history. You/your child will have about 1¼-2 teaspoons (6-9 mL) of blood drawn for routine blood tests (if not collected within the past month), to measure your/your child’s HIV viral load and to test how your/your child’s immune system is working.

STOPPING STUDY TREATMENT EARLY

If you/your child stop(s) taking the study treatment before **week 28**, you/your child will be asked to continue the study visit schedule. Depending on when you/your child stops taking the study treatment, you/your child will have the same procedures that were previously discussed in the sections: *STUDY VISITS (Weeks, 4, 12,* ***20****, and* ***28****)* and *STUDY VISITS (Weeks* ***40*** *&* ***52****)*.

STOPPING THE STUDY EARLY VISIT

If you/your child stop(s) the study before you/your child have/has completed all of the study visits, you/your child will be asked to return to the clinic for a final visit. This visit will include most of the procedures described in the *“Step 1- Entry Visit”* section. This visit will last about 1-2 hours.

FOR NICHD Sites:

With your permission, some of your/your child’s blood specimens collected as part of this study will be stored for testing at a later date. There is a separate consent form to explain this and get your/your child's consent.

For NIAID Sites:

Storage of Blood Samples

With your permission, some of your/your child’s blood will be stored (with usual protectors of identity) and used for future IMPAACT-approved, HIV-related research. The samples will be shipped to NIAID repositories located in the United States. About 10 teaspoons (8x6 mL) of blood will be taken for this purpose.

Your/your child’s samples will be stored at a special laboratory facility. Only approved researchers will have access to them. People who work at the facility will also have access to your/your child’s samples to keep track of them. These people won’t have information that directly identifies you/your child. Your/your child’s samples will not be sold or directly used to produce commercial products. All proposed research studies using your/your child’s samples will be reviewed by the National Institutes of Health (NIH). There is no time limit on how long your/your child’s samples will be stored.

The researchers do not plan to contact you or your/your child’s regular doctor with the results of studies done using your/your child’s stored samples. This is because research studies are often done with experimental procedures. The results of such studies should not be used to make decisions about your/your child’s medical care. If the researchers decide that the result of a certain study provides important information for your/your child’s medical care, your/your child’s study doctor will be notified. If you would like to be contacted with this sort of information, you must notify the study staff of any changes in your address or phone number. You may decide that you do not want your/your child’s samples stored for future research studies. You/your child can still participate in this study even if you make this decision.

You may withdraw your consent for the storage and use of your/your child’s samples at any time. If you withdraw your consent, these stored samples will be destroyed.

Please read the following statement carefully and then mark your initials in the appropriate space provided.

I agree to allow my/my child’s blood samples to be stored for use in future IMPAACT-approved, HIV-related research studies.

__________ Yes __________ No __________ Date

OTHER INFORMATION

Information provided throughout this study about sexual activity and pregnancy test results will not be shared with parents or caretakers of adolescent participants. [Sites should modify the preceding language about confidentiality of sexual activity and pregnancy test results to conform to their local practice, regulations and IRB/EC requirements.]

The information collected in this study may be used for other IMPAACT-approved HIV-related research.

HOW MANY PEOPLE WILL TAKE PART IN THIS STUDY?

About 344 children and adolescents will take part in this study.

HOW LONG WILL I/MY CHILD BE IN THIS STUDY?

You/your child will be in this study for about 1 year.

WHY WOULD THE DOCTOR TAKE ME/MY CHILD OFF THIS STUDY EARLY?

The study doctor may need to take you/your child off the study early without your permission if:

- The study is stopped or cancelled.
- A Data Safety Monitoring Board (DSMB) recommends that the study be stopped early. A DSMB is an outside group of experts that monitors the study.
- You are/your child is not able to attend the study visits as required by the study.

The study doctor may also need to take you/your child off the anti-HIV drug(s) without your permission if:

- Continuing the anti-HIV drug(s) may be harmful to you/your child
- You/your child need(s) a treatment that you/your child may not take while on the study

If you/your child must stop taking the anti-HIV drug(s) before the study is over, the study doctor may ask you/your child to continue to be part of the study and return for some study visits and procedures.

WHAT ARE THE RISKS OF THE STUDY?

Continuing on HAART: The risks of continuing HAART include developing HIV resistance leading to fewer HIV treatment choices, decreasing immune function, and/or becoming ill*.*

Monotherapy: The risks of using 3TC or FTC alone, even if taken properly, include decreasing immune function, increasing viral load, and/or becoming ill. **It is also possible that by discontinuing HAART you will increase the risk of transmission of HIV to a non-infected sexual partner.**

If you/your child are assigned to take 3TC/FTC in this study, these drugs may have side effects, some of which are listed below. Switching from HAART to 3TC/FTC might mean that you/your child experience fewer side effects or more side effects.  Please note that these lists do not include all the side effects seen with these drugs. These lists include the more serious or common side effects with a known, or possible relationship. If you have questions concerning the drug side effects please ask the medical staff at your site.

Use of Combination Antiretroviral Drugs

The use of potent antiretroviral drug combinations may be associated with an abnormal placement of body fat and wasting. Some of the body changes include:

- Increase in fat around the waist and stomach area
- Increase in fat on the back of the neck
- Thinning of the face, legs, and arms
- Breast enlargement

*Nucleoside Analogue*

*Lactic acidosis (elevated lactic acid levels in the blood) and severe hepatomegaly (enlarged liver) with steatosis (fatty liver) that may result in liver failure, other complications or death have been reported with the use of antiretroviral nucleoside analogues alone or in combination. The liver complications and death have been seen more often in women on these drug regimens. Some nonspecific symptoms that might indicate lactic acidosis include: unexplained weight loss, stomach discomfort, nausea, vomiting, fatigue, cramps, muscle pain, weakness, dizziness and shortness of breath.*

Lamivudine (3TC, Epivir®)

*GlaxoSmithKline*

The following side effects have also been associated with use of lamivudine:

If you are infected with both Hepatitis B and HIV, you should be aware that your liver function tests may increase, and symptoms associated with hepatitis (an acute inflammation of the liver) may worsen if lamivudine is stopped. Although most of these cases have resolved without treatment, some deaths have been reported.

- Headache
- Feeling tired
- Dizziness
- Numbness, tingling, and pain in the hands or feet
- Depression
- Trouble sleeping
- Rash
- Upset stomach, vomiting, nausea, loose or watery stools
- Pancreatitis (inflammation of the pancreas), which may cause death. If you develop pancreatitis, you may have one or more of the following: stomach pain, nausea, and vomiting.
- Abnormal pancreatic and liver function blood tests

Emtricitabine (FTC, Emtriva™*)*

*Gilead Sciences*

The following side effects have been associated with the use of emtricitabine:

- Headache
- Dizziness
- Tiredness
- Inability to sleep, unusual dreams
- Loose or watery stools
- Upset stomach (nausea) or vomiting
- Abdominal pain
- Rash, itching, which sometimes can be a sign of an allergic reaction
- Skin darkening of the palms and/or soles
- Increased cough
- Runny nose
- Abnormal liver function tests, which could mean liver damage
- Increases in pancreatic enzyme (substances in the blood), which could mean a problem with the pancreas
- Increased triglycerides
- Increased creatine phosphokinase (CPK), which could mean muscle damage

NOTE: If you are infected with both Hepatitis B and HIV, you should be aware that your liver function tests may increase, and symptoms associated with hepatitis (an acute inflammation of the liver) may worsen if emtricitabine is stopped.

Risks of taking other medications with antiretroviral drugs:

There are certain medications that should not be taken with antiretroviral drugs as they can potentially have bad side effects. It will be important for you to discuss what medicines you are/your child is taking with the study doctors.

Risks of Blood Draws:

There is risk of some discomfort, bruising, or bleeding at the site where the blood is drawn. Occasionally, there is swelling in the area where the needle enters the body and a small risk of fainting and/or infection.

ARE THERE RISKS RELATED TO PREGNANCY?

If you are/your child is having sex that could lead to pregnancy, you/your child must agree not to become pregnant. If you/your child can become pregnant, you/your child must use at least two methods of reliable birth control that you discuss with the study staff throughout the whole study.

If you/your child can become pregnant, you/your child must have a pregnancy test before you/your child can begin the study. The test must show that you are/your child is not pregnant in order for you/your child to participate in the study. If you think you /your child thinks she may be pregnant at any time during the study, you/your child must tell your provider and study staff right away. [Sites should modify the preceding language about confidentiality of pregnancy results to conform to their local practice, regulations and Institutional Review Board (IRB) requirements].

If you/your child becomes pregnant during the study, we will ask you/your child to continue to be observed on the study, however, if you/your child are/is on the 3TC or FTC monotherapy arm, your provider will manage you according to the local standards of care for the duration of your pregnancy as per the recommended guidelines for pregnant women to prevent transmission of HIV to the unborn child. Your provider/doctor will make the decision about which medicine to put you on.

If you/your child become(s) pregnant, you/your child may be registered in the Antiretroviral Pregnancy Registry (<http://www.apregistry.com/reg.htm>) according to your site’s local standard practice.

ARE THERE BENEFITS TO TAKING PART IN THIS STUDY?

If you/your child take(s) part in this study, there may be no direct benefit to you/your child. Information learned from this study may help others who have HIV.

WHAT OTHER CHOICES DO I/DOES MY CHILD HAVE BESIDES THIS STUDY?

Instead of being in this study you have the choice of:

- treatment with prescription drugs available to you/your child
- treatment with experimental drugs, if you/your child qualify(ies)
- no treatment

Please talk to your doctor about these and other choices available to you/your child. Your doctor will explain the risks and benefits of these choices.

WHAT ABOUT CONFIDENTIALITY?

Domestic Sites:

To help us protect your privacy, we have obtained a Certificate of Confidentiality from the National Institutes of Health. With this Certificate, the researchers cannot be forced to disclose information that may identify you, even by a court subpoena, in any federal, state, or local civil, criminal, administrative, legislative, or other proceedings. The researchers will use the Certificate to resist any demands for information that would identify you, except as explained below. The Certificate cannot be used to resist a demand for information from personnel of the United States Government that is used for auditing or evaluation of federally funded projects or for information that must be disclosed in order to meet the requirements of the federal Food and Drug Administration (FDA).

**A description of this clinical trial will be available on** [***www.ClinicalTrials.gov***](http://www.clinicaltrials.gov/)**, as required by U.S. law.  This Web site will not include information that can identify you.  At most, the Web site will include a summary of the results.  You can search this Web site at any time.**

People who may review your records include the Office for Human Research Protections (OHRP), the site institutional review board (IRB)/Ethics Committee (EC) (insert name of site IRB/EC), the National Institutes of Health, study staff, study monitors, and local regulatory authorities.

You should understand that a Certificate of Confidentiality does not prevent you or a member of your family from voluntarily releasing information about you or your participation in this research. If an insurer, employer, or other person obtains your written consent to receive research information, then the researchers may not use the Certificate of Confidentiality to withhold that information.

The Certificate of Confidentiality does not prevent the researchers from disclosing voluntarily, without your consent, information that would identify you as a participant in the research project under the following circumstances. **[If applicable, the list of circumstances is to be provided by sites to indicate the conditions under which voluntary disclosure will be made per site standards.]**

International Sites:

Efforts will be made to keep your/your child’s personal information confidential. We cannot guarantee absolute confidentiality. Your/your child’s personal information may be disclosed if required by law. Any publication of this study will not use your/your child’s name or identify you/your child personally.

**A description of this clinical trial will be available on** [***www.ClinicalTrials.gov***](http://www.clinicaltrials.gov/)**, as required by U.S. law.  This Web site will not include information that can identify you.  At most, the Web site will include a summary of the results.  You can search this Web site at any time.**

Your/your child’s records may be reviewed by the Office for Human Research Protections (OHRP), (insert name of site) IRB/EC, National Institutes of Health (NIH), study staff, study monitors, and local regulatory authorities.

WHAT ARE THE COSTS TO ME?

Taking part in this study may lead to added costs to you and your insurance company. In some cases it is possible that your insurance company will not pay for these costs because you are/your child is taking part in a research study.

Anti-HIV medications prescribed by your/your child’s provider will not be covered by the study. You/your child and/or your/your child’s health insurance will be responsible for purchasing these medications.

WHAT HAPPENS IF I AM/MY CHILD IS INJURED?

If you are/your child is injured as a result of being in this study, you/your child will be given immediate treatment for your injuries. The cost for this treatment will be charged to you or your insurance company. There is no program for compensation either through this institution or the National Institutes of Health (NIH). You will not be giving up any of your legal rights by signing this consent form.

WHAT ARE MY/MY CHILD’S RIGHTS AS A RESEARCH SUBJECT?

Taking part in this study is completely voluntary. You may choose not to take part/not to allow your child to take part in this study or leave this study/take your child out of the study at any time. Your decision will not have any impact on your/your child’s participation in other studies conducted by the NIH and will not result in any penalty or loss of benefits to which you are/your child is otherwise entitled.

We will tell you about new information from this or other studies that may affect your/your child’s health, welfare or willingness to stay in this study. If you want the results of the study, let the study staff know.

WHAT DO I DO IF I HAVE QUESTIONS OR PROBLEMS?

For questions about this study or a research-related injury, contact:

- name of the investigator or other study staff
- telephone number of above

For questions about your/your child’s rights as a research subject, contact:

- name or title of person on the IRB/EC or other organization appropriate for the site
- telephone number of above

SIGNATURE PAGE

If you have read this consent form (or had it explained to you), all your questions have been answered and you agree to take part in this study, please sign your name below.

_____________________ ____________________________________

Participant’s Name (print) Participant’s Signature and Date

____________________________ ____________________________________

Participant’s Legal Guardian (print) Legal Guardian’s Signature and Date

(As appropriate)

________________________ ____________________________________

Study Staff Conducting Study Staff Signature and Date

Consent Discussion (print)

________________________ ____________________________________

Witness’ Name (print) Witness’s Signature and Date

(As appropriate)

APPENDIX VI

FACT SHEET and TEMPLATE CONSENT FORM for

Specimen Storage at Repositories funded by the

National Institute of Child Health and Human Development (NICHD)

PARENT FACT SHEET

When your child joins this NICHD sponsored Study, you will be asked to give permission for having some specimens that the doctor or nurse will take from your child’s body saved in a repository. (A repository is a special laboratory with freezers where specimens like blood or tissue cells and body fluids that are taken from you during a study are kept. Your child’s name will not be on these specimens, only a special study number. The people who run the repository laboratory will not know your child’s name.)

Why have a repository?

Researchers can learn a lot from a study but as time goes by the tests that they used get better or new tests appear, and there is a need to learn more. When study volunteers consent to put specimens in the repository and consent to the researchers doing new tests on the specimens later after their time in the study is ended, these questions can be answered and more can be learned. None of these future studies would happen unless the Institutional Review Board overseeing the repository examines the study and makes sure that your child’s rights are being protected.

How will my child’s privacy be protected?

The only record that your child participated in this NICHD sponsored study is at the clinic where it is kept separate from your child’s health records and locked away.

Your child’s specimens in the repository will not have your child’s name on them. The specimens will have a special study code. It will be the same code that is on your child’s information in the NICHD sponsored Study from your child’s interviews and examinations. Again, none of this information will have your child’s name on it.

**A description of this clinical trial will be available on** [**www.ClinicalTrials.gov**](http://www.clinicaltrials.gov/)**, as required by U.S. law.  This Web site will not include information that can identify your child.  At most, the Web site will include a summary of the results.  You can search this Web site at any time.**

How would a researcher get to use the specimens in the repository?

If a researcher wants to do a test on specimens from the NICHD sponsored repository in the future, he or she will write up the idea and it will have to be approved by a committee to make sure the research is worthwhile. If the idea is approved, then coded specimens and coded information will be given to the researcher. The researcher will not know the names, addresses, or phone numbers of the people who gave the specimens to the repository.

Why wouldn’t I find out the results of the research using my child’s specimens?

You will not receive the results of research done with your child’s specimens. This is because research can take a long time and must use specimens from many people before results are known. Results from research using your child’s specimens may not be ready for many years. Often when studies are first done, it is not always clear how to use the information from the study to change the health care that people receive. So none of these study results is likely to affect your child’s care right now, but they may be helpful to people like your child in the future. Your child’s specimens can last in the freezer for many years and there is no time limit to when studies could be done in the future.

Would I ever be contacted in the future about research using my child’s specimens?

All of the studies to be done in the future on your child’s specimens in the repository will be for the particular reasons that you agreed to. Every study that is planned to use specimens from your child and others from this NICHD sponsored Study has to be reviewed by a special committee of people known as an Institutional Review Board, who are not part of the Study. Their goal is to make sure that what is planned is the same kind of study that you had agreed to. If it is, then the research will go ahead since you would have agreed that these particular tests could be done without anyone contacting you to get your permission in the future.

If the study to be done is not like the kind of tests you agreed could be done, then the committee will decide if you need to be contacted to give permission for the new study.

I gave my permission to testing my child’s specimens in the repository, but what if I change my mind?

People always have the right to stop participating in research. So if you decide that you do not want researchers to be able to use the specimens from your child in the repository, you can contact the clinic staff. They will tell the repository that the specimens with the study code number linked to your child’s name in the clinic should not be studied. These specimens can be removed from the repository and destroyed if you tell us to do that.

What type of research will be done with my child’s specimens?

Many different kinds of studies use specimens. Some researchers may develop new tests to find diseases. Others may develop new ways to treat or even cure diseases. In the future, some of the research may help to develop new products, such as tests and drugs. If this would happen and these tests or drugs make money, there are no plans to share that money with the people who gave the specimens.

As part of this study (insert title), your child is being asked to have some (insert specimen source- blood, urine, tissue, genital fluid, saliva, etc.) taken. These specimens will go into the NICHD repository for research to be done at some time in the future so that more information can come from your child’s time in this NICHD sponsored Study.

You do not have to agree to store your child’s specimens for future tests for your child to take part in this study. Your child will not lose any benefits to which your child is entitled if you decide against storing your child’s specimens.

You will also be asked to agree that these particular tests can be done without anyone contacting you to get your permission sometime in the future. No one doing these tests would know that these specimens came from your child and no one would contact you or your doctor or nurse with the results from these tests that might happen in the future.

TEMPLATE CONSENT FORM

What are the general HIV-related studies that can be done with the repository specimens?

Researchers would like to store your child’s specimens to understand how HIV causes disease and complications, and how best to treat or prevent HIV infection and its complications. They need specimens from people who have HIV and from those who do not. Sometimes, too, the specimens can be used to learn something about new problems that people with HIV have like liver disease, diabetes, and heart disease. These general studies would not include any genetic testing (looking at your child’s DNA).

Benefits: There are no direct benefits to your child. Your child will be helping researchers learn more about how to help people with HIV or at risk of HIV infection.

Risks: The specimens would be collected as part of your child’s study visits. (Insert text about collection procedures.) Once in the repository, there are few risks. Your child’s name will not be available to the repository or to the scientists who may be doing any future test.

I give permission for the use of my child’s stored specimens for the purposes stated in the preceding section (general HIV-related tests).

___________________________ ___________________________ ________

Parent or Legal Guardian Signature Witness Signature Date

I give my assent to the use of my stored specimens for the purposes stated in the preceding section (general HIV-related tests).

___________________________ ___________________________ _________

Participant Signature Witness Signature Date

What are the special HIV-related studies that can be done with the repository specimens?

Researchers in this study would also like to store your child’s specimens to understand how HIV causes disease and complications, and how best to treat or prevent HIV infection and its complications through looking at how each person’s genetic makeup (your child’s DNA) either protects them or puts them at greater risk. It may be that researchers use some of your child’s blood to make a “cell line”. That means the blood cells can keep dividing and give an endless supply of your child’s DNA for tests to be done in the future. This kind of information will be particularly important as scientists work toward a vaccine that could protect people from AIDS. They need specimens from people who have HIV and from those who do not.

Benefits: There are no direct benefits to your child. Your child will be helping researchers learn more about how to help people with HIV or at risk of HIV infection.

Risks: The specimens would be collected as part of your child’s study visits. (Insert text about collection procedures.) Once in the repository, there are few risks. Your child’s name will not be available to the repository or to the scientists who may be doing any future test. Since there are no plans to give participants the results of the tests performed on their stored specimens, you will not receive any information on your child’s genetic makeup.

I give permission for the use of my child’s stored specimens for the purposes stated in the preceding section (special HIV-related tests).

___________________________ ___________________________ _________

Parent or Legal Guardian Signature Witness Signature Date

I give my assent to the use of my stored specimens for the purposes stated in the preceding section (special HIV-related tests).

___________________________ ___________________________ _________

Participant Signature Witness Signature Date

What if I have more questions?

If you have any questions about the repository, about storage, or the use of your child’s samples, contact (Study personnel) at (phone).

If you have questions about giving consent or your child’s rights as a research volunteer, contact the (Name of Institution) Institutional Review Board at (phone).

I refuse to have any specimen collected from my child stored in the repository.

___________________________ ___________________________ _________

Parent or Legal Guardian Signature Witness Signature Date

APPENDIX VII

FACT SHEET and TEMPLATE CONSENT FORM for

Specimen Storage at the Repository of the National Institute of Child Health and Human Development (NICHD)

YOUTH FACT SHEET

When you join this NICHD sponsored Study, you will be asked to consent to having some specimens that the doctor or nurse will take from your body saved in a repository. (A repository is a special laboratory with freezers where specimens like blood or tissue cells and body fluids that are taken from you during the study are kept. Your name will not be on these specimens, only a special study number. The people who run the repository laboratory will not know your name.)

Why have a repository?

Researchers can learn a lot from a study but as time goes by the tests that they used get better or new tests appear, and there is a need to learn more. When study volunteers consent to put specimens in the repository and consent to the researchers doing new tests on the specimens later after their time in the study is ended, these questions can be answered and more can be learned. None of these future studies would happen unless the Institutional Review Board overseeing the repository examines the study and makes sure that your rights are being protected.

How will my privacy be protected?

The only record that you participated in this NICHD sponsored Study is at your clinic where it is kept separate from your health records and locked away.

Your specimens in the repository will not have your name on them, only a special study code. It will be the same code that is on your information in the NICHD sponsored Study from your interviews and examinations. Again, none of this information will have your name on it.

**A description of this clinical trial will be available on** [**www.ClinicalTrials.gov**](http://www.clinicaltrials.gov/)**, as required by U.S. law.  This Web site will not include information that can identify you.  At most, the Web site will include a summary of the results.  You can search this Web site at any time.**

How would a researcher get to use the specimens in the repository?

If a researcher wants to do a test on specimens from the NICHD repository in the future, he or she will write up the idea and it will have to be approved by a committee to make sure the research is worthwhile. If the idea is approved, then coded specimens and coded information will be given to the researcher. The researcher will not know the names, addresses, or phone numbers of the people who gave the specimens to the repository.

Why wouldn’t I find out the results of the research using my specimens?

You will not receive the results of research done with your specimens. This is because research can take a long time and must use specimens from many people before results are known. Results from research using your specimens may not be ready for many years. Often when studies are first done, it is not always clear how to use the information from the study to change the health care that people receive. So none of these study results is likely to affect your care right now, but they may be helpful to people like you in the future. Your specimens can last in the freezer for many years and there is no time limit to when studies could be done in the future.

Would I ever be contacted in the future about research using my specimens?

All of the studies to be done in the future on your specimens in the repository will be for the particular reasons that you agreed to. Every study that is planned to use specimens from you and others from this NICHD sponsored Study has to be reviewed by a special committee of people known as an Institutional Review board, who are not part of the Study. Their goal is to make sure that what is planned is the same kind of study that you agreed to. If it is, then the research will go ahead since you would have agreed that these particular tests could be done without anyone contacting you to get your permission in the future.

If the new study to be done is not like the kind of tests you agreed could be done, then the committee will decide if you need to be contacted to give consent for the new study.

I gave my consent to testing my specimens in the repository, but what if I change my mind?

People always have the right to stop participating in research. So if you decide that you do not want researchers to be able to use the specimens from you in the repository, you can contact the clinic staff. They will tell the repository that the specimens with the study code number linked to your name in the clinic should not be studied. These specimens can be removed from the repository and destroyed if you tell us to do that.

What type of research will be done with my specimens?

Many different kinds of studies use specimens. Some researchers may develop new tests to find diseases. Others may develop new ways to treat or even cure diseases. In the future, some of the research may help to develop new products, such as tests or drugs. If this would happen and these tests or drugs make money, there are no plans to share that money with the people who gave the specimens.

As part of this study (insert title), you are being asked to have some (insert specimen source- blood, urine, tissue, genital fluid, saliva, etc.) taken from you. These specimens will go into the NICHD repository for research to be done at some time in the future so that more information can come from your time in this NICHD sponsored Study.

You do not have to agree to store your specimens for future tests to take part in this study. You will not lose any benefits to which you are entitled if you decide against storing your specimens.

TEMPLATE CONSENT/ASSENT FORM

What are the general HIV-related studies that can be done with the repository specimens?

Researchers would like to store your specimens to understand how HIV causes disease and complications, and how best to treat or prevent HIV infection and its complications. They need specimens from people who have HIV and from those who do not. Sometimes, too, the specimens can be used to learn something about new problems that people with HIV have like liver disease, diabetes, and heart disease. These general studies would not include any genetic testing (looking at your DNA).

Benefits: There are no direct benefits to you. You will be helping researchers learn more about how to help people with HIV or at risk of HIV infection.

Risks: The specimens would be collected as part of your study visits. (Insert text about collection procedures.) Once in the repository, there are few risks. Your name will not be available to the repository or to the scientists who may be doing any future test.

I consent to the use of my stored specimens for the purposes stated in the preceding section (general HIV-related tests).

___________________________ ___________________________ _________

Participant Signature Witness Signature Date

What are the special HIV-related studies that can be done with the repository specimens?

Researchers in this study would also like to store your specimens to understand how HIV causes disease and complications, and how best to treat or prevent HIV infection and its complications through looking at how each person’s genetic makeup (your DNA) either protects them or puts them at greater risk. It may be that researchers use some of your blood to make a “cell line”. That means the blood cells can keep dividing and give an endless supply of your DNA for tests to be done in the future. This kind of information will be particularly important as scientists work toward a vaccine that could protect people from AIDS. They need specimens from people who have HIV and from those who do not.

Benefits: There are no direct benefits to you. You will be helping researchers learn more about how to help people with HIV or at risk of HIV infection.

Risks: The specimens would be collected as part of your study visits. (Insert text about collection procedures.) Once in the repository, there are few risks. Your name will not be available to the repository or to the scientists who may be doing any future test. Since there are no plans to give participants the results of the tests performed on their stored specimens, you will not receive any information on your genetic makeup.

I consent to the use of my stored specimens for the purposes stated in the preceding section (special HIV-related tests).

___________________________ ___________________________ _________

Participant Signature Witness Signature Date

What if I have more questions?

If you have any questions about the repository, about storage, or the use of your samples, contact (Study personnel) at (phone).

If you have questions about giving consent or your rights as a research volunteer, contact the (Name of Institution) Institutional Review Board at (phone).

I refuse to have any specimen collected for storage in the repository.

___________________________ ___________________________ _________

Participant Signature Witness Signature Date
