# Supplementary material for: Decline in CD4 T lymphocytes with monotherapy bridging strategy for non-adherent adolescents living with HIV infection: Results of the IMPAACT P1094 randomized trial
Source: PLoS One. 2017 Jun 12;12(6):e0178075. doi: 10.1371/journal.pone.0178075 (PMC5467803; doi:10.1371/journal.pone.0178075)
Supplement: S1 Table — (DOCX) [file pone.0178075.s003.docx]

S1Table: Baseline Immune Characteristics on 3TC/FTC Monotherapy Arm by Immunologic Deterioration

|  | | Experienced Immunologic Deterioration  (Median (IQR)) | | | |  |
| --- | --- | --- | --- | --- | --- | --- |
| Biomarker |  | | No (N=7) | Yes (N=4) | P-Value* | |
| IL-6 (pg/ml) |  | | 2.12 (1.04, 2.55) | 3.34 (1.37, 4.92) | 0.57 | |
|  | | | | | |  |
| C-Reactive Protein (ug/ml) |  | | 1.15 (0.41, 1.79) | 0.79 (0.40, 9.81) | 0.92 | |
|  | | | | | |  |
| sCD14 (ug/ml) |  | | 2.19 (1.55, 2.67) | 2.43 (1.87, 2.76) | 0.51 | |
|  | | | | | |  |
| SVC (ng/ml) |  | | 1,965 (917, 2,549) | 1,917 (1,260, 2,202) | 0.92 | |
|  | | | | | |  |
| D-Dimer (ng/ml) |  | | 309 (163, 389) | 276 (173, 476) | 0.92 | |
|  | | | | | |  |
| *Wilcoxon Test | | | | | |  |
