# Supplementary material for: Decline in CD4 T lymphocytes with monotherapy bridging strategy for non-adherent adolescents living with HIV infection: Results of the IMPAACT P1094 randomized trial
Source: PLoS One. 2017 Jun 12;12(6):e0178075. doi: 10.1371/journal.pone.0178075 (PMC5467803; doi:10.1371/journal.pone.0178075)
Supplement: S2 Table — (DOCX) [file pone.0178075.s004.docx]

S2 Table: Baseline Immune Activation of 3TC/FTC Monotherapy Arm by Immunologic Deterioration

|  | | Experienced Immunologic Deterioration  (Median (IQR)) | | | |  |
| --- | --- | --- | --- | --- | --- | --- |
|  |  | | No (N=4) | Yes (N=4) | P-Value* | |
| (CD3+/CD4+) CD38+ HLA-DR+ [CD4 activated, CD4A] |  | | 4.0 (2.9, 6.1) | 5.9 (3.8, 11.0) | 0.67 | |
|  | | | | | |  |
| (CD3+/CD4+) CD95+ [CD4 Fas receptor, CD4F] |  | | 31.7 (24.7, 48.4) | 35.4 (25.8, 49.3) | 0.89 | |
|  | | | | | |  |
| (CD3+/CD8+) CD38+ HLA-DR+ [CD8 activated, CD8A] |  | | 36.1 (33.2, 36.4) | 31.6 (19.5, 47.3) | >0.99 | |
|  | | | | | |  |
| (CD3+/CD8+) CD95+ [CD8 Fas receptor, CD8F] |  | | 67.1 (14.8, 71.7) | 36.1 (18.8, 45.8) | 0.38 | |
|  | | | | | |  |
| (CD3+/CD4+) CD27+CD45RO- CD95- [CD4 naïve, CD4N] |  | | 56.6 (45.2, 66.6) | 45.3 (29.3, 61.0) | 0.47 | |
|  | | | | | |  |
| (CD3+/CD8+) CD27+CD45RO- CD95- [CD8 naïve, CD8N] |  | | 23.7 (18.7, 76.4) | 42.9 (21.4, 59.1) | >0.99 | |
|  | | | | | |  |
| (CD3+/CD4+) CD31+ CD27+ CD45RO- CD95- [Recent thymic emigrants, RTE] |  | | 39.4 (32.8, 55.0) | 34.2 (23.9, 53.0) | 0.47 | |
|  | | | | | |  |
| *Wilcoxon Test | | | | | |  |
